# Bioactive Steroids Bearing Oxirane Ring

**DOI:** 10.3390/biomedicines11082237

**Published:** 2023-08-09

**Authors:** Valery M. Dembitsky

**Affiliations:** Centre for Applied Research, Innovation and Entrepreneurship, Lethbridge College, 3000 College Drive South, Lethbridge, AB T1K 1L6, Canada; valery.dembitsky@lethbridgecollege.ca or dvmioch@gmail.com

**Keywords:** steroids, triterpenoids, isoprenoid lipids, antineoplastic, anti-inflammatory, antifungal, antibacterial, antiviral, fungal endophytes, plants, marine invertebrates

## Abstract

This review explores the biological activity and structural diversity of steroids and related isoprenoid lipids, with a particular focus on compounds containing an oxirane ring. These natural compounds are derived from fungi, fungal endophytes, as well as extracts of plants, algae, and marine invertebrates. To evaluate their biological activity, an extensive examination of refereed literature sources was conducted, including in vivo and in vitro studies and the utilization of the QSAR method. Notable properties observed among these compounds include strong anti-inflammatory, antineoplastic, antiproliferative, anti-hypercholesterolemic, antiparkinsonian, diuretic, anti-eczematic, anti-psoriatic, and various other activities. Throughout this review, 3D graphs illustrating the activity of individual steroids are presented, accompanied by images of selected terrestrial or marine organisms. Furthermore, this review provides explanations for specific types of biological activity associated with these compounds. The data presented in this review are of scientific interest to the academic community and carry practical implications in the fields of pharmacology and medicine. By analyzing the biological activity and structural diversity of steroids and related isoprenoid lipids, this review offers valuable insights that contribute to both theoretical understanding and applied research. This review draws upon data from various authors to compile information on the biological activity of natural steroids containing an oxirane ring.

## 1. Introduction

The oxirane ring, known as an epoxy group, also named epoxide group, is an important functional group in organic chemistry [1]. It consists of an oxygen atom bonded to two adjacent carbon atoms through single covalent bonds, forming a three-membered epoxide ring [1,2]. The strained three-membered ring of the oxirane makes it highly reactive. The oxygen atom is electron-rich and can undergo various reactions, such as a nucleophilic attack, ring-opening reactions, and rearrangements [3,4]. Oxiranes can undergo ring-opening reactions with nucleophiles, such as amines, alcohols, and thiols. This process leads to the formation of new carbon–oxygen and carbon–nucleophile bonds [4,5]. Epoxidation is a common reaction that introduces an oxirane ring into a molecule. It involves the addition of an oxygen atom to a double bond using oxidizing agents like peracids, peroxyacids, or peroxides [5,6]. Oxirane rings have widespread applications in organic synthesis and industry. They are used as intermediates in the production of various chemicals, pharmaceuticals, polymers, and coatings. The ring-opening reactions of oxiranes also find applications in organic synthesis to introduce functional groups into molecules [6,7,8].

Steroids bearing an oxirane ring or an α,β-epoxy group are natural or synthetic compounds that possess a reactive epoxy (oxirane) ring structure at the α and β positions of the steroid backbone [2,9,10]. This α,β-epoxy group adds chemical reactivity and contributes to the unique properties and potential biological activities of these compounds. Steroids with an α,β-epoxy group can be found in various natural sources, including fungi, plants, animals, and microorganisms [11,12,13,14,15]. They have also been synthesized in the laboratory for pharmaceutical and research purposes. These compounds often exhibit diverse pharmacological activities and are of interest in drug discovery and development. Steroids bearing an α,β-epoxy group exhibit unique chemical reactivity and can interact with various biological targets, including enzymes, receptors, and signaling pathways. Their potential biological activities and therapeutic applications continue to be explored in fields such as medicine, pharmacology, and biochemistry [14,15,16,17,18,19,20].

The presented steroids bearing an α,β-epoxy group can be categorized into six groups based on the position of the epoxy group (ring). These groupings provide a systematic classification of these compounds. The groups and their respective positions are stated in the following: Group 1: 4,5-epoxy steroids, Group 2: 5,6-epoxy steroids, Group 3: 7,8- and 8,9-epoxy steroids, Group 4: 9,11- and 11,12-epoxy steroids, Group 5: 17,20-epoxy steroids and 24,25-epoxy steroids, and Group 6: miscellaneous epoxy steroids.

**Figure 1:**
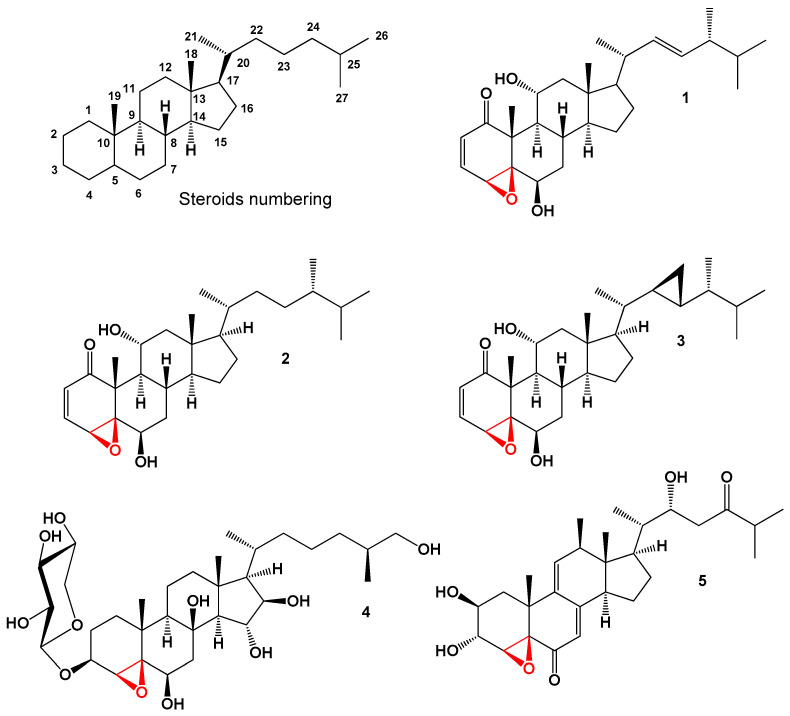
Steroids bearing 4,5-epoxy group.

The positioning of the epoxy group within the steroid molecule plays a significant role in the compound’s structure, reactivity, and potential biological activities. Understanding the precise location of the epoxy group is essential for studying the properties and interactions of these compounds. To facilitate the identification and numbering of steroids, Figure 1 displays the recommended numbering system by the International Union of Pure and Applied Chemistry (IUPAC). This numbering system assists in standardizing the representation and communication of steroid structures, ensuring clarity and consistency in the scientific literature and research.

The data presented in Table 1, Table 2, Table 3, Table 4, Table 5, Table 6, Table 7, Table 8, Table 9 and Table 10 are taken from published data and obtained using the German computer software PASS (http://www.akosgmbh.de/mobile/pass.htm). This program is in the public domain and is used by more than 26,000 scientists from around the world annually. The site of this program provides complete information on the use, as well as the interpretation, of the data obtained.

By categorizing and numbering steroids bearing an α,β-epoxy group, researchers and scientists can effectively study their structure–activity relationships, biological functions, and potential therapeutic applications in various fields, including medicine, pharmacology, and biochemistry.

## 2. Steroids Bearing a 4,5-Epoxy Group

Steroids bearing a 4,5-epoxy group are a specific subset of steroids that possess an epoxy (oxirane) ring structure at the 4th and 5th positions of the steroid backbone. This unique configuration contributes to their distinct chemical and biological properties. Steroids bearing a 4,5-epoxy group may exhibit unique biological activities and have been investigated for their potential therapeutic applications. The presence of the epoxy group can influence the compound’s interactions with receptors, enzymes, and other molecular targets, leading to specific physiological effects. Research on steroids with a 4,5-epoxy group aims to understand their mechanisms of action, structure–activity relationships, and potential pharmacological applications. By exploring the properties and activities of these compounds, scientists strive to uncover new insights into their roles in health, disease, and therapeutic interventions [9,10,11,12,13,16,17,18,19,20].

**Figure 2:**
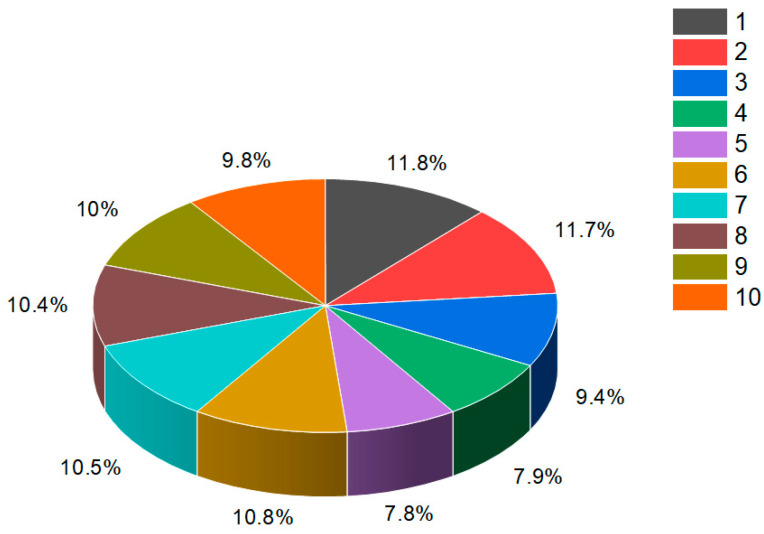
Percentage distribution of biological activities associated with a steroid bearing a 4,5-epoxy group (**1**) derived from the gorgonian extract of *Leptogorgia sarmentosa*. This steroid exhibits a wide range of pharmacological properties, making it a compound of significant interest. The graph provides valuable information regarding the distribution of the biological activities associated with this specific steroid. It highlights the various pharmacological effects and potential therapeutic applications that have been observed or predicted for compound **1**. Dominant and additional activities are indicated under the following numbers: 1. *apoptosis agonist* (11.7%), 2. *antineoplastic* (11.7%), 3. *antineoplastic (liver cancer)* (9.4%), 4. *prostate cancer treatment* (7.9%), 5. *antineoplastic (lymphocytic leukemia)* (7.8%), 6. *anti-hypercholesterolemic* (10.8%), 7. *immunosuppressant* (10.5%), 8. *hepatic disorders treatment* (10.4%), 9. *anti-eczematic* (10%), and 10. *anti-psoriatic* (9.8%). By understanding the percentage distribution of these biological activities, researchers can gain insights into the compound’s multifaceted nature and explore its potential uses in different areas of pharmacology. These activities may include but are not limited to cytotoxicity, anti-inflammatory effects, antimicrobial properties, or interactions with specific receptors or enzymes. The wide range of pharmacological properties exhibited by steroid **176** from the gorgonian extract underscores its potential as a valuable resource for drug discovery and development. Further studies are likely necessary to fully elucidate the mechanisms of action and to assess the compound’s safety and efficacy profiles. The exploration of steroids bearing a 4,5-epoxy group and their biological activities contributes to our understanding of the diverse pharmacological potential of natural compounds. It underscores the importance of investigating marine sources and their unique chemical constituents for the discovery of new therapeutic agents and the advancement of modern medicine.

The gorgonian extract of *Leptogorgia sarmentosa*, a type of coral, has been found to contain three cytotoxic steroids: (20*S*)-20-hydroxy-cholestane-3,16-dione (**1**), (16*S*,20*S*)-16,20-dihydroxycholestan-3-one (**2**), and (20*S*)-20-hydroxycholest-1-ene-3,16-dione (**3**). These compounds are commonly known as yonarasterols D, E, and F, respectively [21]. The biological activities of these steroids have been investigated, and their cytotoxic properties have been demonstrated against four tumor cell lines. The percentage distribution of the biological activity of steroid (**1**) is depicted in Figure 2, providing insights into its potency and efficacy. Studies have shown that these isolated steroids exhibit significant cytotoxicity, with an effective dose (ED_50_) of 1 μg/mL against the tested tumor cell lines. This cytotoxic activity suggests their potential as candidates for further exploration in cancer research and drug development. The identification and characterization of these cytotoxic steroids derived from the gorgonian extract highlight the rich biodiversity of marine sources and their potential as a valuable reservoir of bioactive compounds. The investigation of these compounds contributes to the ongoing efforts to discover new therapeutic agents for the treatment of cancer and other diseases.

**Figure 3:**
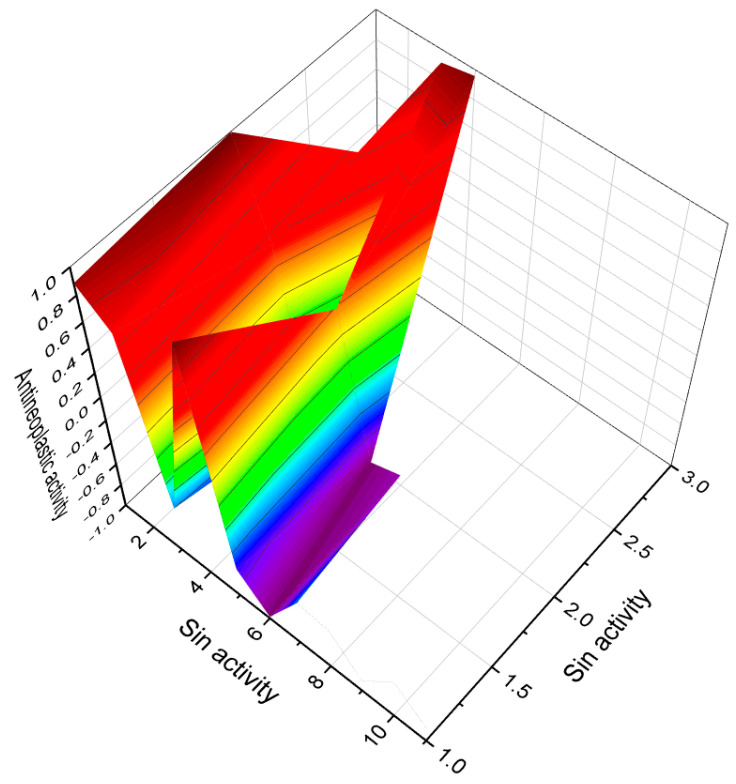
Predicted and calculated antineoplastic activity of steroids bearing a 4,5-epoxy group (**1**, **4**, and **5**) derived from marine sources. The graph provides valuable insights into the potential effectiveness of these compounds as antineoplastic agents, with a confidence level of over 90%. Antineoplastic activity refers to the ability of a substance to inhibit or prevent the growth and proliferation of cancer cells. The evaluation of the antineoplastic activity of these specific steroids is of great interest due to their unique chemical structures derived from marine sources. Steroid **1**, obtained from the gorgonian extract of *Leptogorgia sarmentosa*, exhibits significant cytotoxicity against tumor cell lines. Steroid **4**, known as kurilensoside H, isolated from the Far Eastern starfish *Hippasteria kurilensis*, possesses a 4,5-epoxy functionality and shows potential antineoplastic properties. Steroid **5**, an unprecedented non-sulfated sterol derived from the marine sponge *Xestospongia* sp., exhibits antineoplastic activity along with inhibition of HIV-1 integrase. The 3D graph demonstrates the predicted and calculated activity of these steroids as antineoplastic agents. A confidence level over 90% indicates a high degree of reliability in these predictions. Understanding the potential antineoplastic activity of steroids bearing a 4,5-epoxy group from marine sources is crucial for identifying new compounds for cancer treatment and drug development. Red is strong activity, Blue—poor acvtivity.

**Figure 4:**
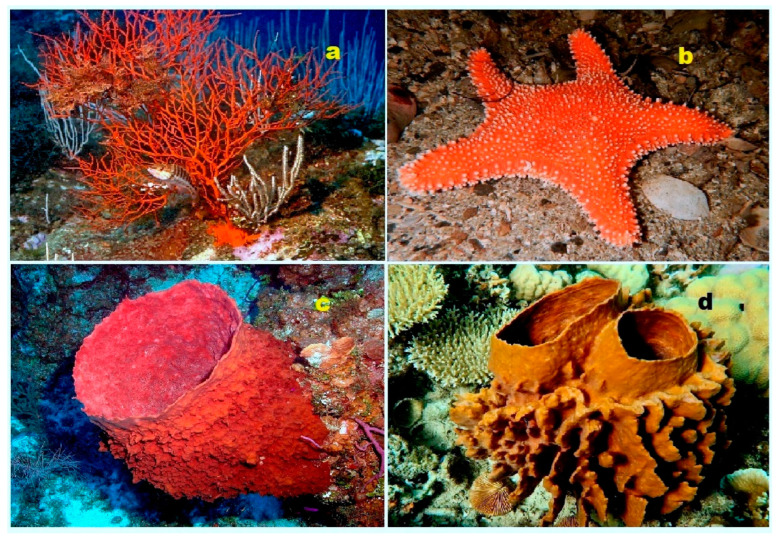
Specimens of marine organisms that have been the source of the discussed steroids. In particular, image (**a**) represents the gorgonian *Leptogorgia sarmentosa*, which is known to contain steroids (**1**–**3**). The image (**b**) corresponds to the Far Eastern starfish *Hippasteria kurilensis*, which is the source of steroid (**4**). The image (**c**) represents the marine sponge *Xestospongia* sp., which has yielded steroid (**5**). The image (**d**) provides another perspective of the marine sponge *Xestospongia* sp., highlighting its significance as a source of the mentioned steroid. These visual representations of the marine organisms help to establish a connection between the natural sources and the steroids under discussion. By observing these organisms, one can appreciate the diverse habitats and ecological niches from which these compounds are derived. The use of such imagery in scientific research aids in the documentation, identification, and understanding of marine organisms and their associated bioactive compounds.

Monoglycoside kurilensoside H (**4**) has been isolated from the alcoholic extract of the Far Eastern starfish *Hippasteria kurilensis*, which was collected near the Kuril Islands. The chemical structure of kurilensoside H is depicted in Figure 3, and a sample of the starfish is shown in Figure 4. Notably, the aglycon moiety of kurilensoside H represents the second known instance of marine polar steroids containing a 4,5-epoxy functionality. This unique feature adds to the compound’s chemical novelty and biological significance [22]. Another remarkable discovery is the identification of an unprecedented non-sulfated sterol, 4β,5β-epoxy-2β,3α,12β,22*S*-tetrahydroxy-14α-methylcholest-7,9(11)-dien-6,24-dione (**5**), derived from a marine sponge species, *Xestospongia* sp., obtained from the Philippines. This sterol exhibits a complex structure with a 4,5-epoxy group and multiple hydroxy groups. Importantly, it has been found to act as an inhibitor of HIV-1 integrase, making it a potential candidate for anti-HIV therapeutic research [23]. The discovery of compounds like kurilensoside H and sterol **5** further underscores the vast chemical diversity and biological potential of marine organisms. These findings contribute to our understanding of the unique natural products derived from the marine environment and their potential applications in medicine and drug development.

## 3. Steroids Bearing a 5,6-Epoxy Group

Steroids bearing a 5,6-epoxy group are a specific class of compounds that possess a cyclic ether functional group at the 5th and 6th carbon positions of the steroid backbone [2,9,10]. These steroids exhibit unique chemical structures and often display interesting biological activities. The specific biological activities and applications of steroids bearing a 5,6-epoxy group may vary depending on the compound and its chemical structure. Further research is needed to fully understand the pharmacological potential and therapeutic applications of these compounds. As an example, there are three known types of 5,6-epoxy steroids: *Epoxycholesterol*, this steroid is characterized by a 5,6-epoxy functionality and is found naturally in certain marine organisms and plants. It has been investigated for its potential effects on cholesterol metabolism and as a precursor for the synthesis of bioactive compounds. *Epoxyprogesterone*, this steroid derivative contains a 5,6-epoxy group and is structurally related to progesterone. It has been studied for its hormonal activities and potential applications in reproductive medicine. *Epoxyandrostenedione*, this compound is an androstenedione derivative that possesses a 5,6-epoxy group. It has been explored for its potential as an androgenic or estrogenic agent and its effects on hormone regulation [24,25,26].

**Figure 5:**
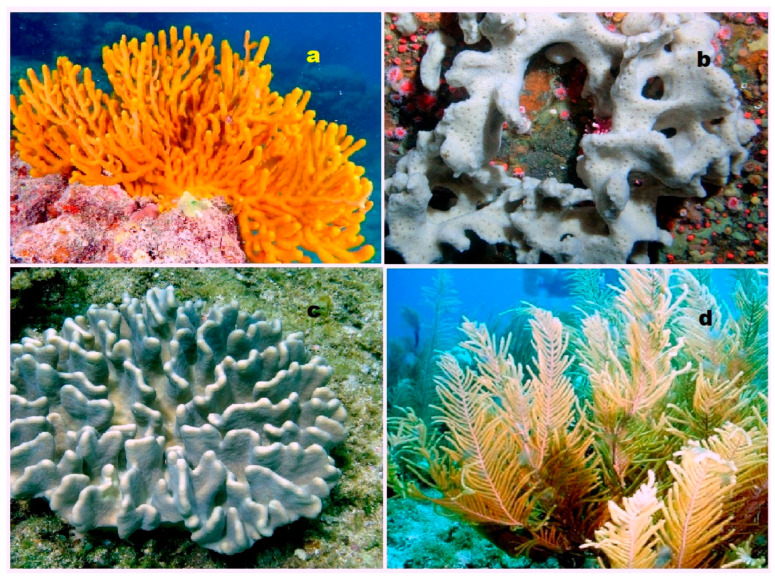
Steroids bearing the 5,6-epoxy group have been found in some marine invertebrates. Thus, steroid (**10**) was found in the gorgonian *Isis hippuris* (**a**); other epoxy steroids (**7–9**) were isolated from the marine sponge of *Ircinia aruensis* (**b**); steroid (**13**) was found in the soft coral *Lobophytum* sp. (**c**), while steroid (**12**) was isolated from the soft coral *Pseudopterogorgia americana* (**d**).

**Figure 6:**
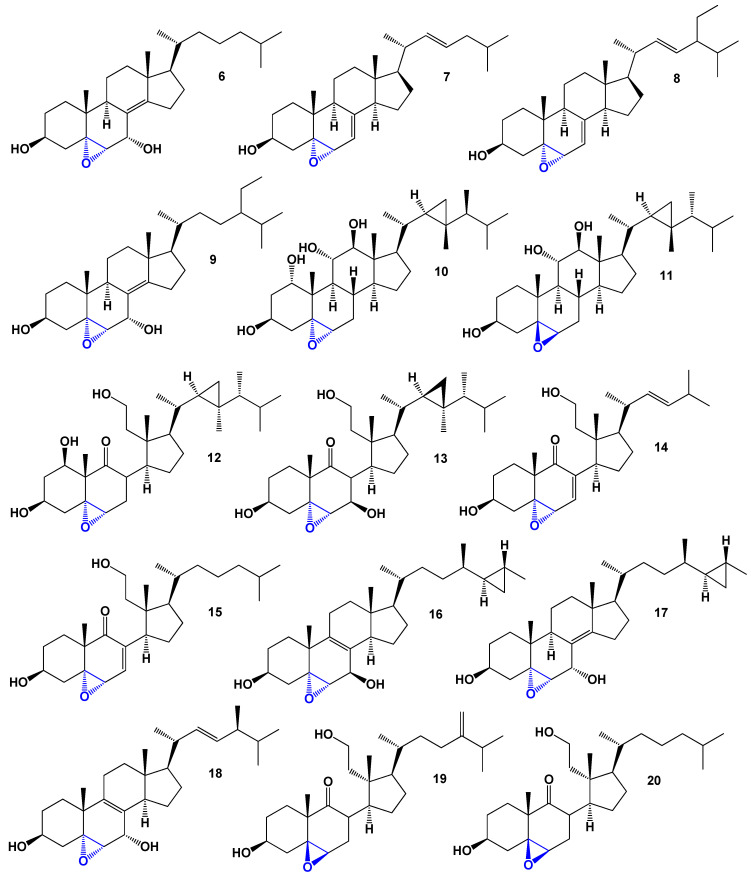
Structures of 5,6-epoxy steroids (**6**–**20**) derived from marine sources. Blue is 5,6-epoxy group.

The ethanolic extract of the marine sponge *Ircinia aruensis* yielded several cytotoxic epoxy steroids, including 5α,6α-epoxystigmasta-7-en-3β-ol (**6**) and three other compounds (**7**–**9**). These compounds have shown significant cytotoxic activity and were isolated from the sponge specimen of *I. aruensis*, as depicted in Figure 5 [27]. Another noteworthy discovery is the polyhydroxysteroid isihippurol B (**10**) obtained from the MeOH extract of the gorgonian *Isis hippuris*. The structure of isihippurol B is depicted in Figure 6, and its biological activity is detailed in Table 2. This polyhydroxysteroid showcases unique chemical characteristics and possesses significant biological activity [28]. Additionally, a rare poly-hydroxysteroid, (1α,3β,5β,6β,11α,15α)-5,6-epoxy-gorgostane-1,3,11,15-tetrol (**11**), was discovered in the extract of the gorgonian *Isis hippuris*. This compound represents a unique example of a polyhydroxysteroid bearing a 5,6-epoxy group [29]. The identification and characterization of these steroidal compounds from marine sources expand our understanding of the chemical diversity present in marine organisms. Their unique structures and demonstrated biological activities provide valuable insights into their potential applications in various fields, including medicine and drug development.

**Figure 7:**
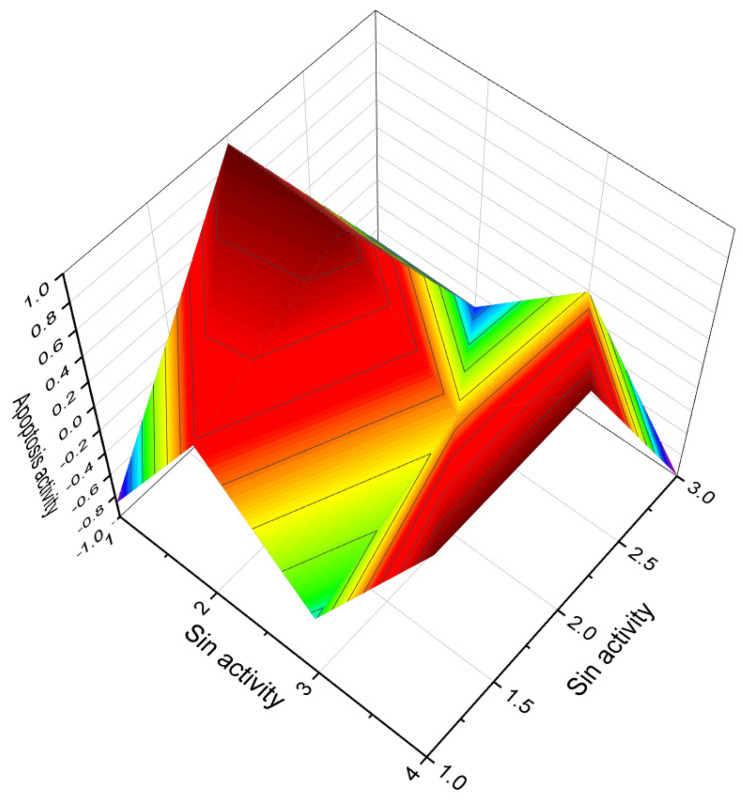
3D graph illustrating the predicted and calculated apoptosis activity of steroids bearing a 5,6-epoxy group. Specifically, compounds **7**, **8**, and **18**, which were isolated from marine sources, are depicted in the graph. The graph demonstrates the correlation between the chemical structure of these compounds and their potential to induce apoptosis. The high confidence level of over 95% suggests a strong likelihood of their apoptotic activity. Apoptosis is a programmed cell death process that plays a crucial role in various biological processes, including development, tissue homeostasis, and elimination of damaged or potentially harmful cells. The induction of apoptosis is an important mechanism for anticancer agents as it can selectively target and eliminate cancer cells. This information highlights the significance of these compounds in the field of cancer research and suggests their potential as promising candidates for the development of anticancer therapies. Further studies and experimental validations are usually conducted to confirm the apoptotic activity of these compounds and explore their mechanisms of action.

The soft coral *Pseudopterogorgia americana* produces an antiproliferative compound called (1β,3β,5α,6α)-5,6-epoxy-1,3,11-trihydroxy-9,11-seco-gorgostan-9-one (**12**) [30], which is an epoxy secosterol. In addition, three 5,6-epoxy secosterols were discovered in the soft coral *Lobophytum* sp. (**13**) [31], and two epoxy secosterols named **14** and **15** were found in the sponge *Aplysilla glacialis* [32]. Notably, both **14** and **15** exhibited anticancer activity. Furthermore, the extract of the Far Eastern sponge *Geodinella robusta* contained topsentisterols B2 (**16**), B3 (**17**), and B4 (**18**) [33]. These compounds are epoxy steroids with either an α- or β-hydroxyl group positioned at position 17. A three-dimensional graph of topsentisterol B4 (**18**) is shown in Figure 7.

**Figure 8:**
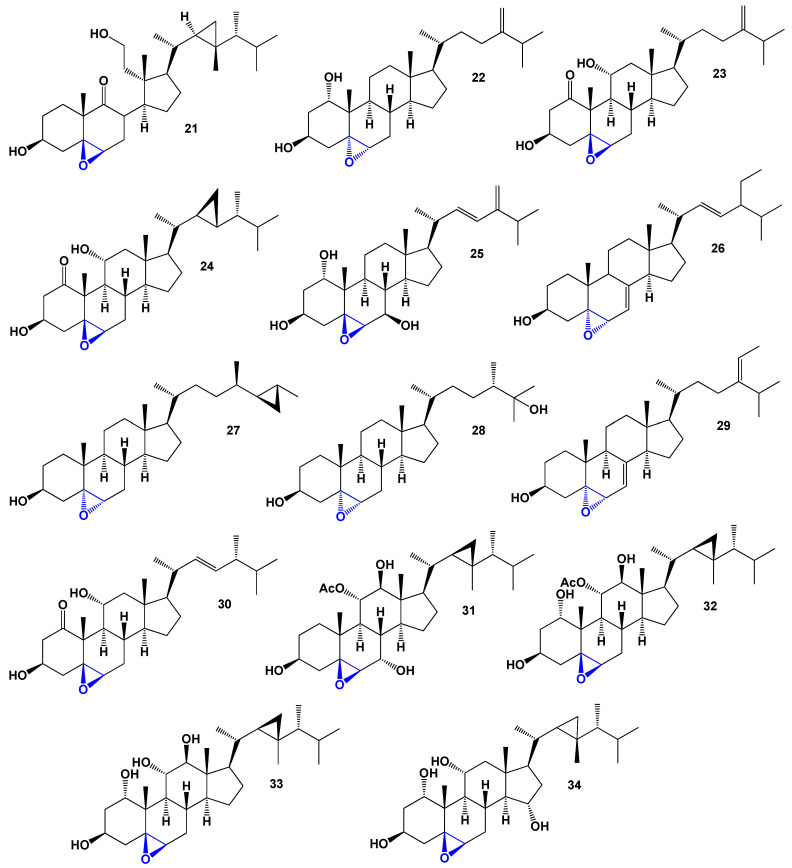
Steroids bearing a 5,6-epoxy group (**21**–**34**). Blue is 5,6-epoxy group.

The octocoral *Sinularia lochmodes*, collected in the waters of Taiwan, produces cytotoxic steroids (**19**–**21**) [34,35]. Their structures can be seen in Figure 8, and the biological activity is summarized in Table 3. Among these steroids, gibberoepoxysterol (**22**) displayed mild activity. In addition, the colonial soft coral *Clavularia viridis*, found on Green Island, Taiwan, yielded two mildly cytotoxic compounds known as stoloniferones I (**23**) and J (**24**) [36]. Another mildly cytotoxic compound, sinugrandisterol D (**25**), a trihydroxylated sterol, was isolated from *Sinularia grandilobata* in Kenting, Taiwan [37].

**Figure 9:**
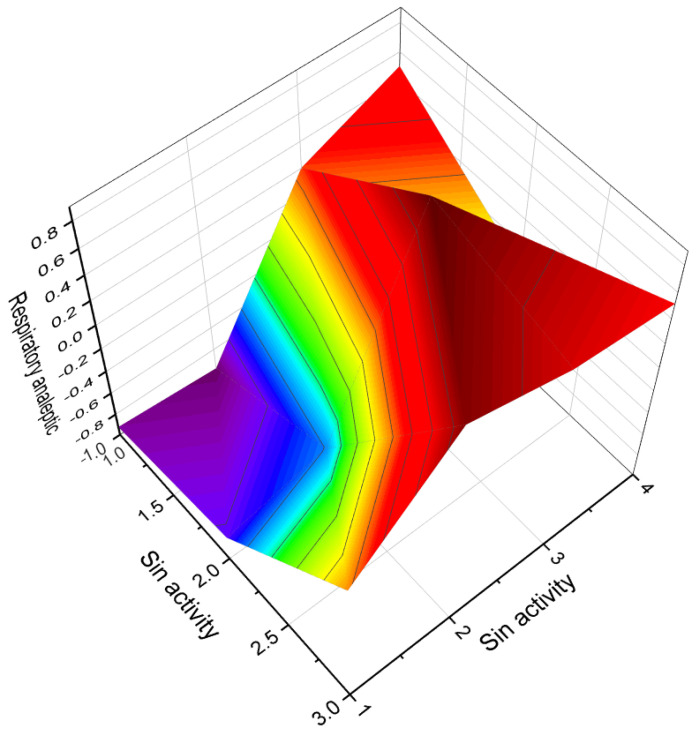
3D graph illustrating the predicted and calculated respiratory analeptic activity of steroids bearing a 5,6-epoxy group. Specifically, compounds **24**, **27**, and **28**, which were isolated from marine sources, are represented in the graph. The graph provides insights into the relationship between the chemical structure of these compounds and their potential to exhibit respiratory analeptic activity. Respiratory analeptic activity refers to the ability of certain compounds to stimulate and enhance respiratory function, particularly in cases of respiratory depression or impairment. The respiratory analeptic activity shown by three 5,6-epoxy steroids, two from the soft coral *Clavularia viridis* (**24**) and the coral *Sarcophyton trocheliophorum* (**27**) and a third steroid (**28**) from the marine sponge *Ianthella* sp., can be called a great success. These compounds can have therapeutic potential in conditions such as respiratory distress or respiratory depression caused by various factors. The high confidence level of over 97% suggests a strong likelihood of their respiratory analeptic activity. This indicates that these compounds have the potential to stimulate and enhance respiratory function. However, it is important to note that further experimental studies and validations are typically required to confirm the respiratory analeptic activity of these compounds and to explore their underlying mechanisms.

The sponge *Ircinia aruensis*, collected from Naozhou, China, produced the epoxysterol (**26**), which exhibited moderate cytotoxic activity [38]. Furthermore, the elephant ear sponge *Ianthella* species, found in Namyet, Vietnam, yielded 5,6α-epoxy-petrosterol (**27**), which displayed cytotoxic properties and induced apoptosis [39]. Additionally, a poly-hydroxy steroid called zahramycin A (**28**, a 3D graph can be seen in Figure 9) was isolated from the polar fraction of the extract obtained from the coral *Sarcophyton trocheliophorum* [40].

The (24*E*)-5α,6α-epoxystigmasta-7,24(28)-dien-3β-ol (**29**) was isolated from the South China Sea sponge *Phyllospongia foliascens*; however, its biological activity has not been studied [41]. *Clavularia viridis* has been found to produce (3α,5β,6β,11α,22*E*,24*R*)-5,6-epoxy-3,11-dihydroxyergost-22-en-1-one (**30**), and although its structure has been determined, its activity has not been investigated [42]. The bamboo coral *Isis hippuris* is a source of polyhydroxylated sterols (**31**–**34**) that exhibit antiviral activity against human cytomegalovirus [43,44]. These sterols possess multiple hydroxyl groups and have shown potential as antiviral agents.

## 4. Steroids Bearing 7,8- and 8,9-Epoxy Groups

Steroids bearing a 7,8-epoxy group are a specific class of steroids that possess an epoxy functional group at the 7th and 8th carbon positions [2,9,10]. This modification alters the chemical structure of the steroid and can potentially impart unique biological activities and properties. The biological activities and functions of steroids bearing a 7,8-epoxy group can vary depending on their specific chemical structure and context. They may exhibit diverse activities such as modulation of lipid metabolism, regulation of nuclear receptors, or involvement in inflammatory processes. Further research is often required to fully understand their biological functions and potential therapeutic applications. Some examples of steroids bearing a 7,8-epoxy group are included in the following. *7α,8α-Epoxycholesterol*: This steroid is a naturally occurring oxysterol found in various biological sources. It has been implicated in cholesterol metabolism and as a precursor in the synthesis of steroid hormones [10]. *7α,8α-Epoxy-24(S)-hydroxycholesterol*: This compound is a metabolite of cholesterol and has been identified as a potent endogenous agonist for the liver X receptor (LXR), a nuclear receptor involved in cholesterol homeostasis. *7α,8α-Epoxy-5α-chol-6-en-3β-ol*: This steroid is a derivative of cholesterol and has been investigated for its potential anti-inflammatory and antioxidant properties [10]. *7α,8α-Epoxy-5α-cholane-3β,6α-diol*: This compound is a bile acid derivative and has been studied for its role in regulating cholesterol and bile acid metabolism [10].

Steroids bearing an 8,9-epoxy group are a specific class of steroids that possess an epoxy functional group at the 8th and 9th carbon positions [2,9,10,15]. This modification alters the chemical structure of the steroid and can potentially impart unique biological activities and properties. Some examples of steroids bearing an 8,9-epoxy group are included in the following. *8α,9α-Epoxy-5β,6β-epoxycholestan-3β-ol:* This compound is a steroidal alkaloid found in certain marine sponges. It has been investigated for its cytotoxic and antiproliferative activities against cancer cells. *8α,9α-Epoxy-3α-hydroxycholest-4-en-6-one*: This steroid is a synthetic compound that has been studied for its anti-inflammatory and antitumor properties. It has shown potential as an inhibitor of inflammation and as a suppressor of tumor cell growth. *8α,9α-Epoxy-5α-cholestane-3β,7α-diol*: This compound is a naturally occurring sterol found in certain marine organisms. It has been investigated for its potential antiviral activity against human cytomegalovirus (HCMV). *8α,9α-Epoxy-5α,6β-epoxycholestan-3β-ol*: This steroidal alkaloid is isolated from marine organisms and has exhibited cytotoxic activity against cancer cells. The biological activities and functions of steroids bearing an 8,9-epoxy group can vary depending on their specific chemical structure and context. They may possess cytotoxic, antiproliferative, anti-inflammatory, antitumor, or antiviral properties [9,10,15].

**Figure 10:**
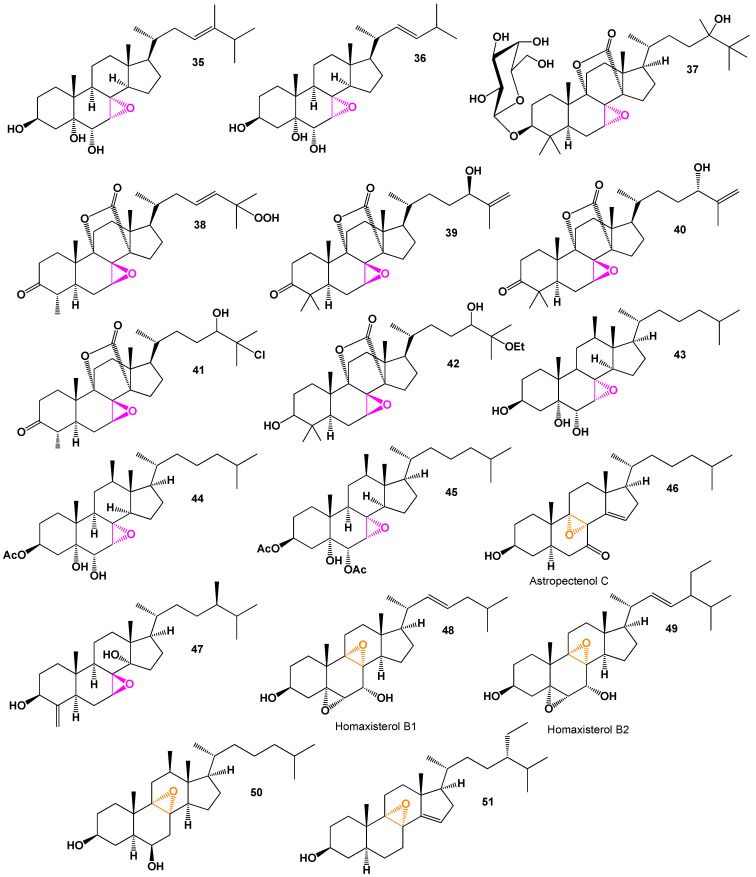
Steroids bearing 7,8- and 8,9-epoxy group(s). Crimson indicates 7,8- and light brown indicates 8,9-epoxy group(s).

**Figure 11:**
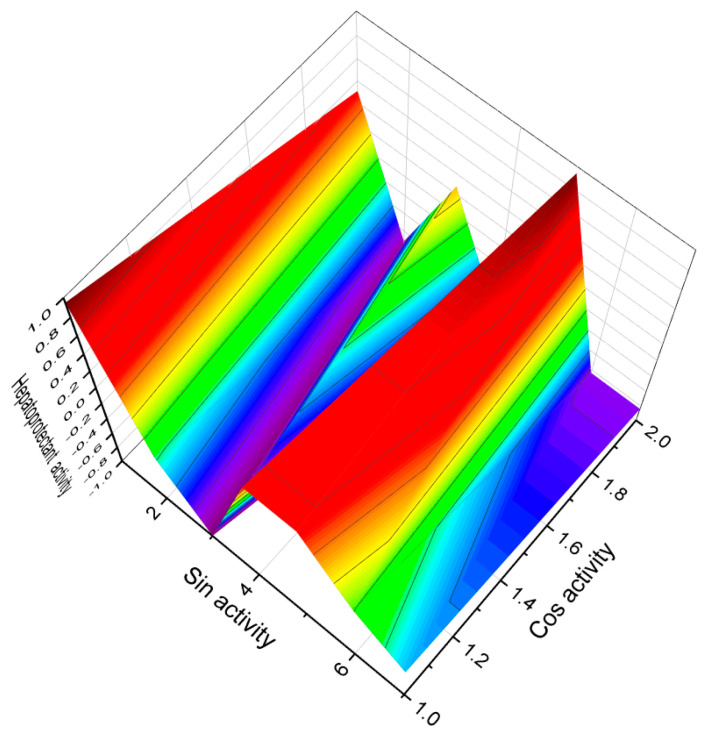
A 3D graph showing the predicted and calculated hepatoprotective activity of a steroid bearing a 7,8-epoxy group from the sponge *Erylus goffrilleri* (**37**) with over 99% confidence. It is known that aqueous or aqueous-alcoholic extracts of the leaves or roots of some plants, such as *Andrographis rissumte, Eclipta alba, Phyllanthus maderaspatensis, Picrorrhiza kurroa, Silibum marianum* or *Trichopus zeylanicus*, have a hepatoprotective effect. However, the fact that the steroid bearing a 7,8-epoxy group from the sponge *Erylus goffrilleri* has hepatoprotective activity with the highest degree of certainty is an extremely rare case.

Two rare 7,8-epoxy polyhydroxysteroids, namely **35** and **36**, were obtained from the extract of the gorgonian *Acabaria undulata* [45]. The structures of these compounds can be seen in Figure 10, and their biological activity is summarized in Table 4. Additionally, a triterpene glycoside called eryloside U (**37**), bearing the 7,8-epoxide group, was isolated from the sponge *Erylus goffrilleri*, which was collected near Arresife-Seko Reef in Cuba [46]. A 3D graph representing eryloside U (**37**) is depicted in Figure 11. Furthermore, several similar oxidized lanostane and nor-lanostane derivatives (**38**–**42**) were isolated from a sponge *Penares* sp., which was collected from the waters of Vietnam [47]. These compounds likely exhibit unique structural features due to the presence of the 7,8-epoxide group. The biological activities and potential therapeutic applications of these compounds are typically studied to explore their pharmacological significance and potential for drug development.

The gorgonian *Acabaria undulata* yielded three steroids (**43**–**45**) that share a common structural feature of a 7α,8α-epoxy-3β,5α,6α-trihydroxyl functionality. These steroids exhibited moderate cytotoxicity and demonstrated inhibitory activity against phospholipase A2 [48].

**Figure 12:**
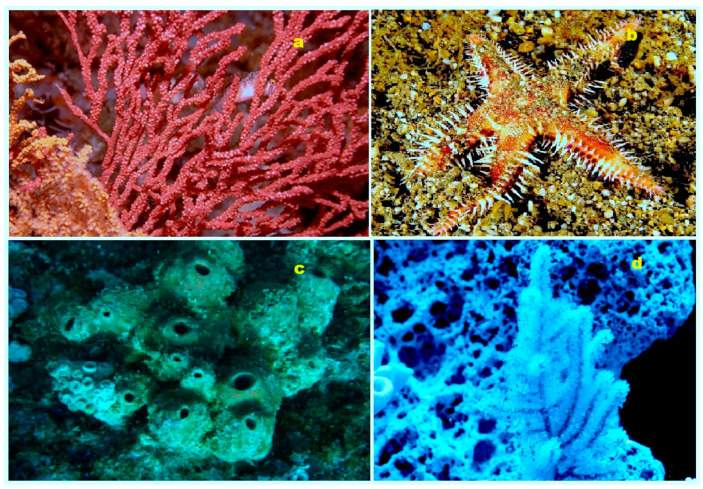
Steroids bearing 7,8- and 8,9-epoxy groups have been discovered in various marine organisms. These include the gorgonian *Acabaria undulata* (**43**–**45**) (**a**), the starfish *Astropecten polyacanthus* (**46**) (**b**), the sponge *Theonella swinhoei* (**47**) (**c**), and the soft coral *Pseudopterogorgia elisabethae* (**50**) (**d**).

**Figure 13:**
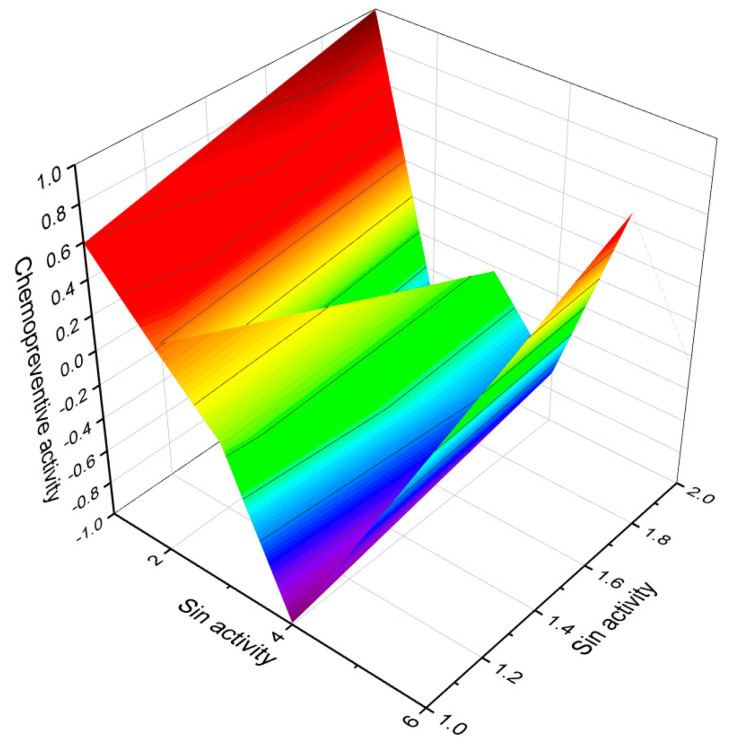
3D graph depicting the predicted and calculated chemopreventive activity of steroids bearing an 8,9-epoxy group derived from the sponges *Homaxinella* sp. (**48**) and *Microscleroderma spirophora* (**51**). The graph provides insights into the relationship between the chemical structures of these compounds and their potential to exhibit chemopreventive activity. Chemopreventive activity refers to the ability of certain compounds to prevent, inhibit, or slow down the development or progression of cancer. These compounds can potentially interfere with carcinogenesis, suppress tumor growth, or enhance the body’s natural defense mechanisms against cancer cells. The high confidence level of over 90% indicates a strong likelihood of their chemopreventive activity. This suggests that these compounds may possess properties that could help prevent or inhibit the development of cancer. However, it is important to note that further experimental studies and validations are usually required to confirm the chemopreventive activity of these compounds and to investigate their underlying mechanisms. Overall, this information highlights the potential significance of these compounds in the field of cancer research and suggests their potential as candidates for the development of chemopreventive strategies.

Astropectenol C (**46**), a rare steroid bearing an 8,9-epoxy group, was obtained from a methanol extract of the starfish *Astropecten polyacanthus* [49]. It possesses cytotoxic properties. Another compound, (3β,5ξ,7β,8β,14α,24*R*)-7,8-Epoxy-14-methoxy-4-methyleneergostan-3-ol (**47**), was isolated from the sponge *Theonella swinhoei* [50]. A sample of this sponge is shown in Figure 12. The cytotoxic polyoxygenated sterols homaxisterols B1 (**48**, a 3D graph shown in Figure 13) and B2 (**49**) were isolated from the MeOH extract of the marine sponge *Homaxinella* sp. These sterols are characterized by their unique 5,6:8,9-diepoxy structure, which was isolated from a marine organism for the first time [51]. These compounds highlight the diverse array of bioactive steroids bearing epoxy groups found in marine organisms. Their cytotoxicity and inhibitory activity against specific enzymes make them potential candidates for further exploration and potential applications in various biomedical fields.

Elistanol (**50**) was isolated from both the aqueous ethanolic and cold hexane extracts of dried soft coral *Pseudopterogorgia elisabethae* collected from Puerto Rico [52]. This compound is obtained from the coral and likely possesses unique biological properties. Furthermore, the Senegalese marine sponge *Microscleroderma spirophora* yielded (3β,8α,9α,24*S*)-8,9-epoxy-3-methoxy-stigmast-14-ene (**51**) [53]. The 3D graph representing the structure of compound **51** is shown in Figure 13. The isolation of this compound from the marine sponge suggests its potential significance in the field of marine natural product research. Both compounds, elistanol (**50**) and (3β,8α,9α,24*S*)-8,9-epoxy-3-methoxy-stigmast-14-ene (**51**), highlight the diversity of bioactive compounds that can be obtained from marine sources [52,53].

## 5. Steroids Bearing 8,14-, 9,11- and 11,12-Epoxy Groups

Steroids bearing different epoxy groups at specific carbon positions exhibit unique structural features and potentially possess distinct biological activities. Following are examples of steroids bearing specific epoxy groups. Steroids bearing an 8,14-epoxy group: One example is 8α,14α-epoxy-5α-cholan-3β-ol (also known as chenodeoxycholic acid epoxide), which is a derivative of chenodeoxycholic acid [10,15]. This compound has been studied for its potential as an inhibitor of cholesterol absorption. Steroids bearing a 9,11-epoxy group: An example is 9α,11α-epoxy-17α-hydroxy-5α-androstan-3-one, which is a synthetic steroid. It has been investigated for its potential anti-inflammatory and immunosuppressive properties. Steroids bearing an 11,12-epoxy group: An example is 11α,12α-epoxy-5α-androstan-3,17-dione (also known as adrenosterone epoxide). This compound is a derivative of adrenosterone and has been studied for its potential as an anti-inflammatory agent and its effect on steroid metabolism. These examples demonstrate the diversity of steroids bearing specific epoxy groups and their potential roles in various physiological processes [9,10,15].

**Figure 14:**
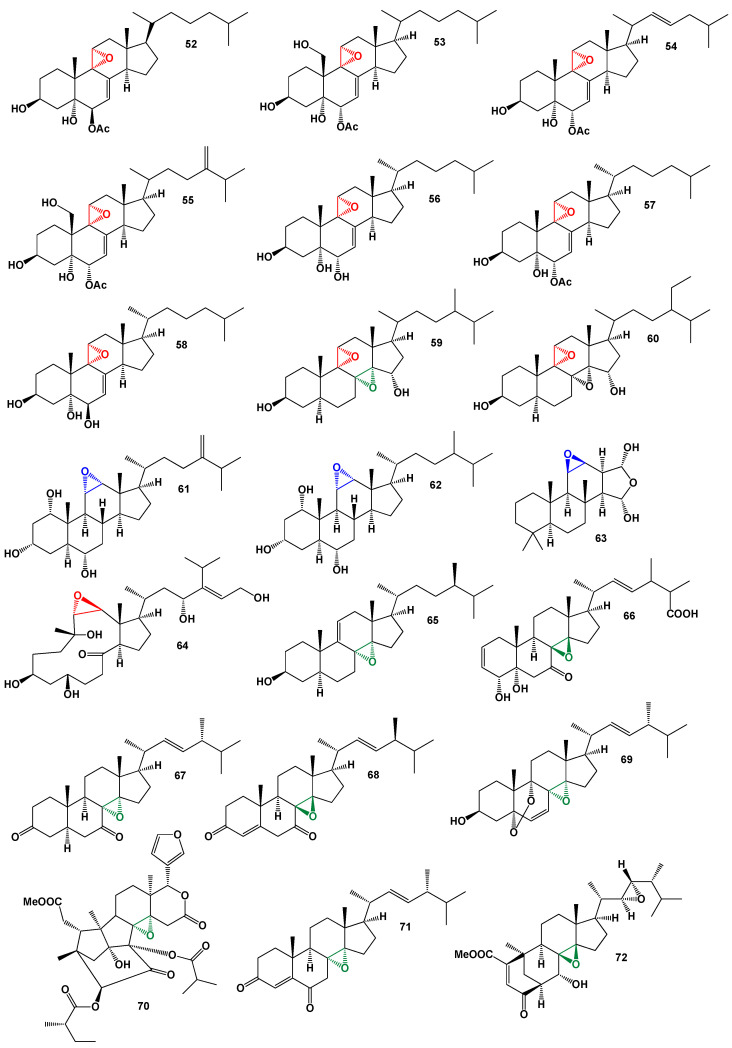
Steroids bearing 8,14-, 9,11- and 11,12-epoxy groups. Red indicates 8,14-, Blue indicates 9,11- and black indicates 11,12-epoxy groups.

Steroids containing 8,14-epoxy groups (**52**–**60**), 9,11-epoxy groups (**61**–**64**), and 11,12-epoxy groups (**65**–**72**, structures are shown in Figure 14, and activity is shown in Table 5) are naturally found in small amounts in various sources. The distribution of these steroids spans fungi, plants, and marine invertebrates, showcasing their wide occurrence in the natural world. For instance, a polyoxygenated steroid with a 9,11-epoxy group (**52**) was isolated from the crude extract of the marine sponge *Dysidea* sp. Collected in Australia. This compound exhibited inhibitory activity against the binding of [I^125^] interleukin-8 [IL-8] to the human recombinant IL-8 receptor type A [54].

**Figure 15:**
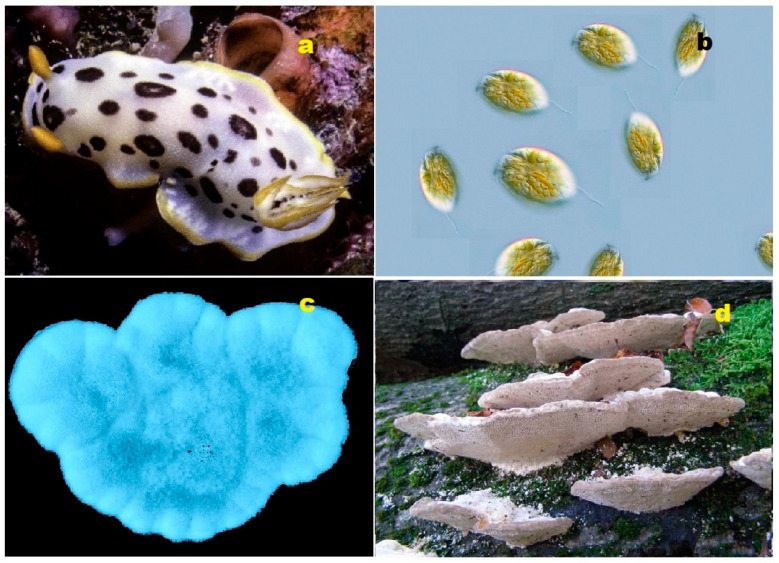
Steroids bearing 8,14-, 9,11-, and 11,12-epoxy groups have been identified in various organisms. These include the nudibranch *Chromodoris obsolete* (**63**) (**a**); the marine dinoflagellate *Amphidinium gibbosum* (**64**) (**b**); the fungus *Aspergillus versicolor* (**68**) (**c**); and the lumpy bracket mushroom *Trametes rossum* (**70**) (**d**). *Trametes rossum*, also known as the tubercle brace, is a tinder fungus that causes white rot and is typically found on beech stumps and dead wood of other hardwoods. Extracts from this fungus have exhibited pharmacological properties such as antioxidant, anti-inflammatory, and anticancer effects.

In the same *Dysidea* genus, Dysideasterol G (**53**), 19-deoxy-dysideasterol A (**54**), dysideasterol C (**55**), and dysideasterol B (**56**) were identified in the active organic extract of an Okinawan marine sponge. These compounds displayed cytotoxic effects against human epidermoid carcinoma A431 cells, with IC_50_ values ranging from 0.15 to 0.3 µM [55]. Additionally, (3β,5α,6α,9α,11α)-9,11-epoxycholest-7-ene-3,5,6-triol (**57**) and (**58**) were isolated from the sponge *Dysidea* sp. [54,56], while (3β,5α,6β,9α,11α)-9,11-epoxycholest-7-ene-3,5,6-triol (**59**) was obtained from the marine gastropod *Planaxis sulcatus* [57]. These 9,11-epoxy steroids exhibit distinct structural variations. Furthermore, the sponge *Theonella swinhoei* from the Solomon Islands (Malaita and Vangunu Is.) yielded conicasterol F (**59**) and theonellasterol I (**60**) [58]. These compounds show potential in modulating bile acid homeostasis in the liver, thereby offering possibilities for the management of metabolic disorders (Figure 15).

The soft corals *Sinularia dissecta* and *Sinularia* sp. From southern India have been a source of bioactive polyhydroxy steroids (**61** and **62**) [59], respectively. These compounds, derived from the soft corals, possess multiple hydroxyl groups and exhibit potential biological activities. In addition, two highly oxygenated steroids, (11β,12β,15α,16α)-11,12-epoxy-15,16-spongianediol (**63**), were isolated from the nudibranchs *Chromodoris obsolete* (Mollusca) [60]. These steroids contain an 11,12-epoxy group and demonstrate unique structural features. Gibbosterol A (**64**), a water-soluble 14-membered carbocyclic steroid with a twisted trans-9,11-epoxy ring, was discovered from the South China Sea dinoflagellate *Amphidinium gibbosum* [61]. This compound exhibits notable agonistic effects against the human pregnane-X-receptor. The discovery of these compounds highlights the diverse sources of steroids bearing 11,12-epoxy and trans-9,11-epoxy groups and their potential as biologically active molecules.

Two steroids bearing the rare 8,14-epoxy group have been identified in marine sponge extracts and marine-derived fungi. These compounds showcase the unique structural variations found in natural sources. One of these unusual steroids, (3β,5α,8α,14α,24*R*)-8,14-epoxy-3-methoxyergost-9(11)-ene (**65**), was detected in the sponge *Jereicopsis graphidiophora* [62]. This compound features an 8,14-epoxy group, along with additional functional groups, and is sourced from the marine environment. Another steroid, (22*E*)-25-carboxy-8β,14β-epoxy-4α,5α-dihydroxyergosta-2,22-dien-7-one (**66**), was found in two marine-derived fungi species: *Aspergillus flavus* [63] and *Acremonium fusidioides* RZ01 [64]. This compound exhibits the rare 8,14-epoxy functionality along with other substituents.

The Red Sea marine sponge *Biemna ehrenbergi* has been found to contain ehrenasterol, identified as (22*E*)-ergosta-22-ene-8,14-epoxy-3,7-dione (**67**) in an organic extract [65]. This unique compound, with its 8,14-epoxy group, is derived from the marine sponge. A similar epoxy ergostane sterol, named versisterol (**68**), was isolated from *Aspergillus versicolor*, an endophytic fungus found in *Avicennia marina* [66]. Versisterol shares the characteristic 8,14-epoxy group and was identified in the fungal extract.

**Figure 16:**
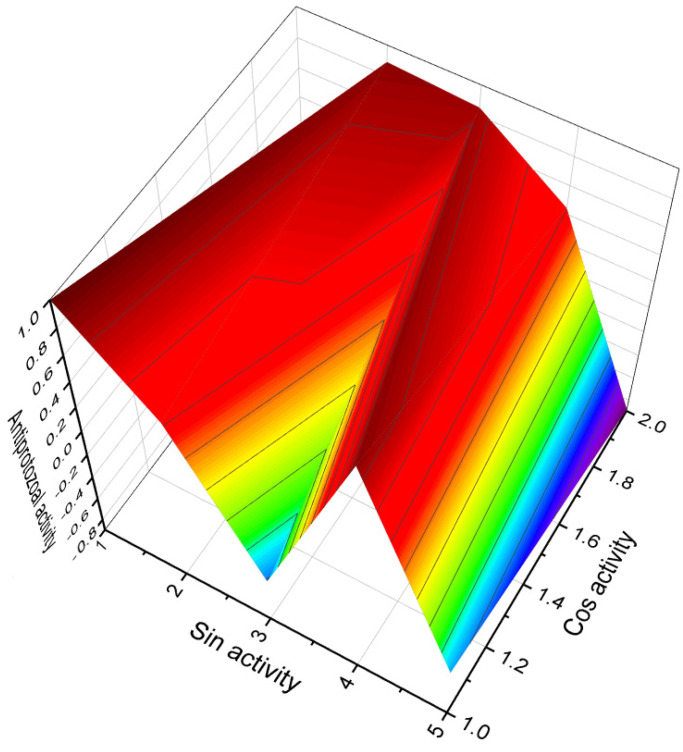
A 3D graph showing the predicted and calculated antiprotozoal activity of a steroid bearing an 8,14-epoxy group from fungus *Papulaspora immersa* (**70**) with over 94% confidence. The 3D graph mentioned would typically represent the relationship between the chemical structure of the steroid and its predicted and calculated antiprotozoal activity. With an accuracy level of over 94% confidence, the graph suggests a strong likelihood of the compound’s antiprotozoal activity. Antiprotozoal activity refers to the ability of a compound to combat or inhibit the growth and survival of protozoan parasites. These parasites can cause diseases such as malaria, leishmaniasis, and toxoplasmosis. The presence of the 8,14-epoxy group in the steroid from *Papulaspora immersa* may contribute to its potential antiprotozoal properties.

Edible mushrooms, *Pleurotus eryngii* and *Panellus serotinus*, produce a sterol known as 5α,9α-epidioxy-8α,14α-epoxy-(22*E*)-ergosta-6,22-dien-3β-ol (**69**). This compound, which exhibits the three-dimensional structure shown in Figure 16, has also been found in extracts from the lumpy bracket mushroom, *Trametes rissum* [67,68]. These mushrooms are recognized as sources of the sterol compound with its unique 8,14-epoxy group. Furthermore, a khayanolide-type limonoid with a 2-carbonyl group, named krishnolide A (**70**), was isolated from the seeds of the Indian mangrove *Xylocarpus moluccensis*. The collection site for the seeds was the mangrove swamp of Krishna estuary in Andhra Pradesh. Krishnolide A, which contains an 8,14-epoxy group, exhibited moderate anti-human immunodeficiency virus (HIV) activity [69]. The discovery of these compounds highlights the diverse sources and potential biological activities associated with the 8,14-epoxy group.

A rapidly growing fungus called *Papulaspora immersa* was isolated from the roots and leaves of *Smallanthus sonchifolius*, a plant belonging to the Asteraceae family, which is commonly known as Yacon. The fungus was cultivated using rice as a growth medium. During the isolation process, an ergostane-type steroid with an 8,14-epoxy group, identified as (22*E*,24*R*)-8,14-epoxyergosta-4,22-diene-3,6-dione (**71**), was discovered in the ethyl acetate fraction of the fungus [70]. This unusual compound possesses a unique structure and is derived from the fungal culture. In addition, a distinct ergostane-type steroid named phomopsterone A (**72**) was isolated from the plant-derived fungus *Phomopsis* sp. TJ507A [71]. Phomopsterone A exhibits an unusual structure and is synthesized by the fungus obtained from plants. The discovery of this compound highlights the diverse range of bioactive compounds that can be derived from plant-associated fungi.

## 6. Steroids Bearing a 17,20-Epoxy Group

Steroids bearing a 17,20-epoxy group are a specific class of steroids that possess an epoxy functional group at the 17th and 20th carbon positions [2,9,10]. This modification alters the chemical structure of the steroid and can potentially impart unique biological activities and properties. However, it is important to note that steroids with a 17,20-epoxy group are relatively rare compared to other types of epoxy steroids, and their biological activities are not as extensively studied. One example of a steroid bearing a 17,20-epoxy group is 17α,20α-epoxyprogesterone, also known as pregnenolone hemisuccinate. This compound is a synthetic derivative of progesterone and has been used in medical research and as a pharmaceutical intermediate [9,10]. The biological activities and functions of steroids bearing a 17,20-epoxy group can vary depending on their specific chemical structure and context.

**Figure 17:**
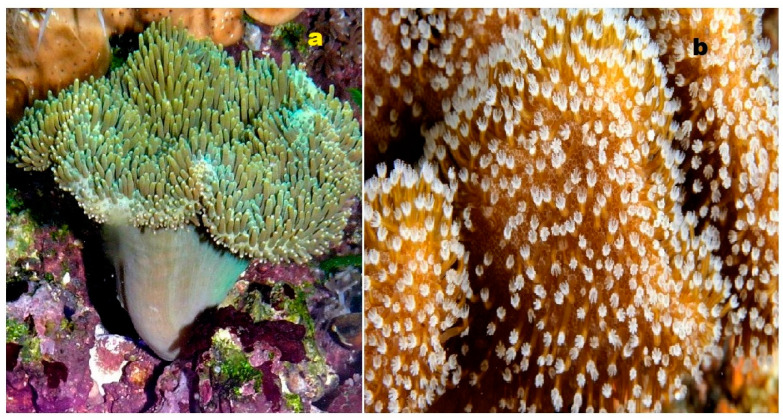
Steroids bearing the 17,20-epoxy group have been identified in the soft coral *Sarcophyton crassocaule* (**73**, **74**, and **85**) (**a**,**b**). This Formosan soft coral species has been discovered in the Western Pacific, New Caledonia, Taiwan, and Ryukyu Island waters. It serves as a rich source of biologically active metabolites, exhibiting various pharmacological properties. These include anti-inflammatory effects, cytotoxicity against human gastric adenocarcinoma cells, antiproliferative activity, and the ability to induce apoptosis in gastric carcinoma cells.

**Figure 18:**
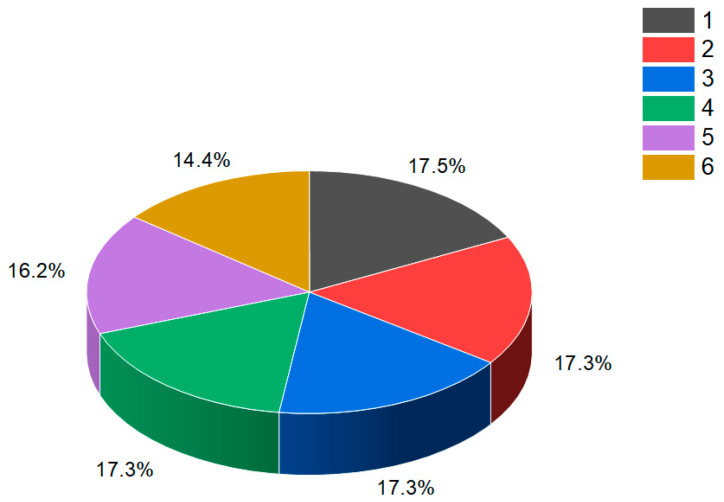
The compound bearing a 17,20-epoxy group (**73**) found in the soft coral *Sarcophyton crassocaule* exemplifies a diverse range of pharmacological properties. Its percentage distribution of biological activities highlights its multifunctional nature. Dominant and additional activities are listed under the following numbers: 1. *Anesthetic general* (17.5%), 2. *Angiogenesis inhibitor* (17.3%), 3. *Respiratory analeptic* (17.3%), 4. *Antineoplastic* (17.3%), 5. *Anti-hypercholesterolemic* (16.2%), and 6. Immunosuppressant (14.4%).

**Figure 19:**
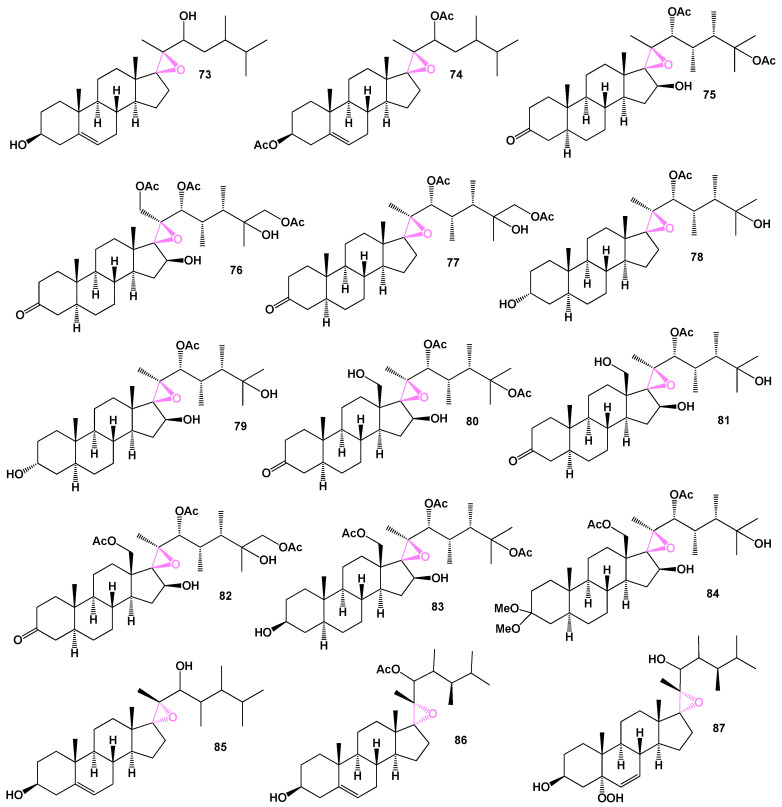
Steroids bearing a 17,20-epoxy group. Magenta indicates the 17,20-epoxy group.

Two unusual steroids, 17β,20β-epoxy-23,24-dimethylcholest-5-ene-3β,22-diol (**73**) and its 3β,22-diacetate (**74**), were discovered in the Indian Ocean soft coral *Sarcophyton crassocaule* (example see in Figure 17) [72]. These compounds exhibit a unique 17β,20β-epoxy group and a specific chemical structure. Figure 18 presents the percentage distribution of the biological activity associated with steroid **73**. In addition, a (22*R*,23*S*,24*S*)-polyoxygenated steroid named hippuristerone A (**75**) was isolated from the Taiwanese gorgonian *Isis hippuris* [73]. This compound possesses an unusual 17β,20β-epoxy group along with multiple oxygenated functional groups. Further investigations in the same line of research led to the isolation of 17β,20β-epoxy (22*R*,23*S*,24*S*)-steroids, known as hippuristerones E−I (**76**–**79**), from the gorgonian coral *Isis hippuris* [74]. The structures of these compounds are depicted in Figure 19, and their biological activities are summarized in Table 6. These discoveries highlight the presence of unique steroids bearing a 17β,20β-epoxy group in soft corals and gorgonian corals. The investigation of their biological activities provides insights into their potential roles and applications in various fields of research.

**Figure 20:**
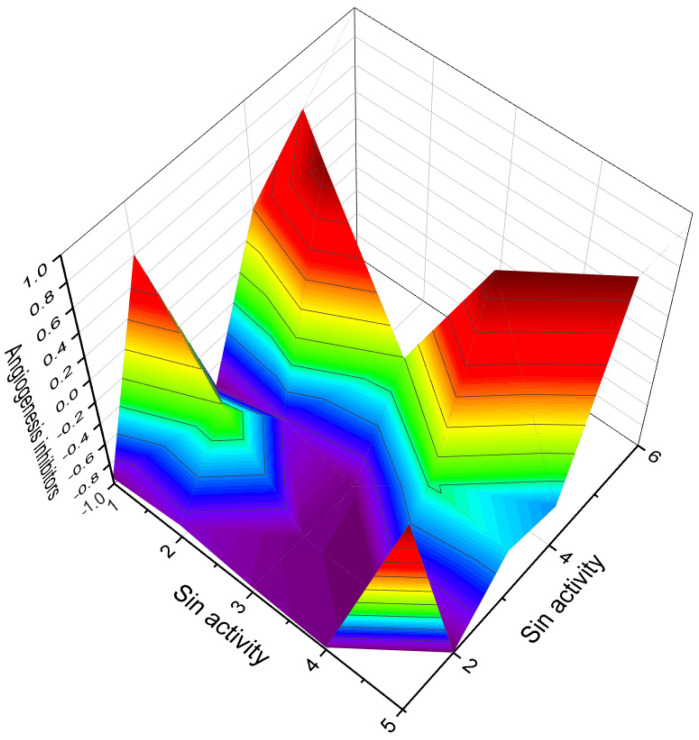
3D graph illustrating the predicted and calculated activity of steroids bearing the 17,20-epoxy group, derived from soft corals *Sarcophyton crassocaule* (**73**, **74**, **75**, and **85**) and *Isis hippuris* (**77**), as potent angiogenesis inhibitors. This graph demonstrates the relationship between their chemical structures and their inhibitory effects on angiogenesis. The predicted and calculated activity values have been determined with a high confidence level of over 90%. Red is strong activity, blue is poor activity.

The polyoxygenated steroids, hipposterone M–O (**80**–**82**), hipposterol G (**83**), and hippuristeroketal A (**84**), isolated from the Taiwanese octocoral *Isis hippuris*, were investigated [75]. These pure compounds demonstrated inhibitory activity against human cytomegalovirus, with an EC_50_ value of 6 μg/mL. Additionally, the soft coral *Sarcophyton crassocaule* produced a compound known as (3β,17β,20R,22ζ,23ζ,24ζ)-17,20-epoxy-23-methylergost-5-ene-3,22-diol (**85**) [76]. The three-dimensional graph of compound **85** is depicted in Figure 20.

Two steroids, namely (22*R*,23*S*)-3β-hydroxy-23-methyl-17,20-epoxyergost-5-en-22-yl acetate (**86**) and (22*R*,23*S*)-5-hydroperoxy-23-methyl-5α-17,20-epoxyergost-6-ene-3β,22-diol (**87**), have recently been discovered from the soft coral *Lobophytum* sp. Found in the South China Sea [77].

## 7. Steroids Bearing a 22,23-Epoxy Group

Steroids bearing a 22,23-epoxy group are a specific type of steroid compound characterized by the presence of an epoxy (oxygen bridge) moiety at the 22nd and 23rd positions of the steroid nucleus [2,9,10]. This modification adds structural complexity and functional diversity to the steroid molecule. These are just a few examples of steroids bearing a 22,23-epoxy group. The presence of this functional group can confer unique biological activities and pharmacological properties to these compounds. *Epoxymexerenone*: This is a synthetic steroidal compound with potential antitumor and anti-inflammatory activities. It has been studied for its inhibitory effects on cancer cell growth. *22,23-Dihydrostigmasterol*: This plant sterol is a precursor for the synthesis of various steroidal compounds. It is found in many plant species and is often used as a marker for plant-based foods. *Lobophytumol A*: It is a diterpenoid steroid isolated from the soft coral *Lobophytum rissum*. It possesses anti-inflammatory and cytotoxic activities [15].

**Figure 21:**
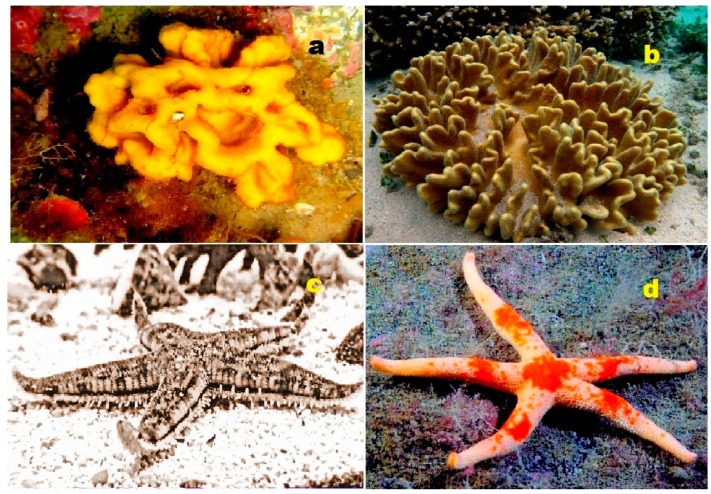
Steroids bearing the 22,23-epoxy group have been discovered in various marine organisms. These include the marine sponge *Axinella* cf. *bidderi* (**88** and **89**) (**a**), the soft coral *Lobophytum rotundum* (**90**) (**b**), the Vietnamese starfish *Archaster typicus* (**118**) (**c**), and the starfish *Henricia downeyae* (**112**, **113**, and **114**) (**d**). These findings highlight the presence of this specific steroid group in diverse marine species, indicating its potential importance in their biological activities.

Two diol 22,23-epoxy steroids have been discovered from the marine sponge *Axinella* cf. *bidderi*. These compounds are identified as 17α-hydroxy-22,23-epoxycholest-5-en-3β-ol (**88**) and 17α-hydroxy-22,23-epoxy-24-methylcholest-5-en-3β-ol (**89**). In vitro studies have demonstrated that these isolated steroids exhibit activity against cell lines derived from the prostate, ovary, pancreas, colon, and lung [78]. Furthermore, an extract obtained from the soft coral *Lobophytum rotundum*, collected from the Pangea Reef in Zanzibar, has yielded a unique compound known as 3β-acetoxy-20,22-epoxy-24-norcholestane (**90**) [79]. A sample of this coral is illustrated in Figure 21.

**Figure 22:**
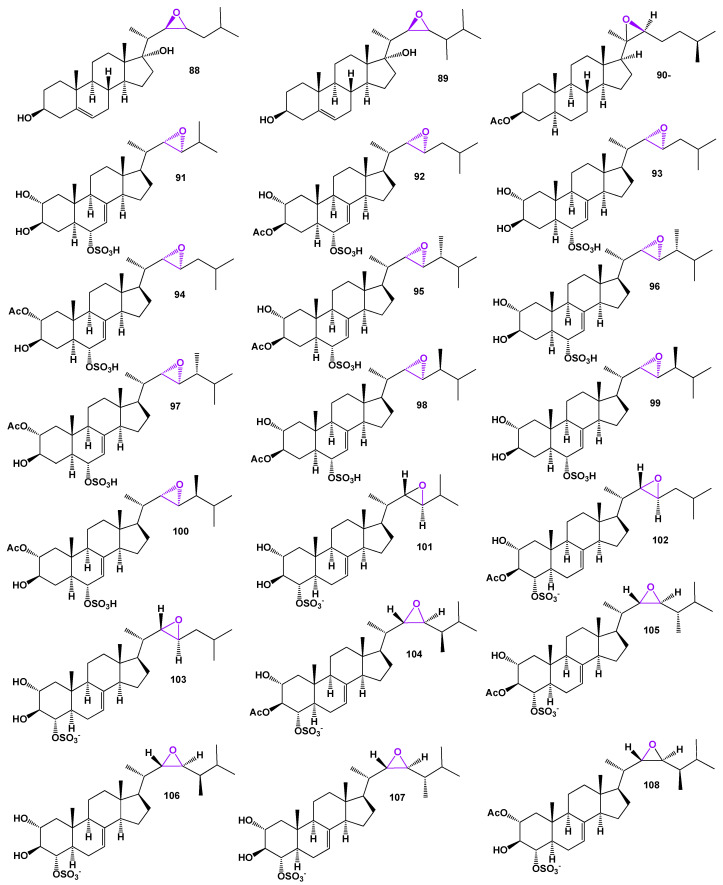
Steroids bearing a 22,23-epoxy group.

**Figure 23:**
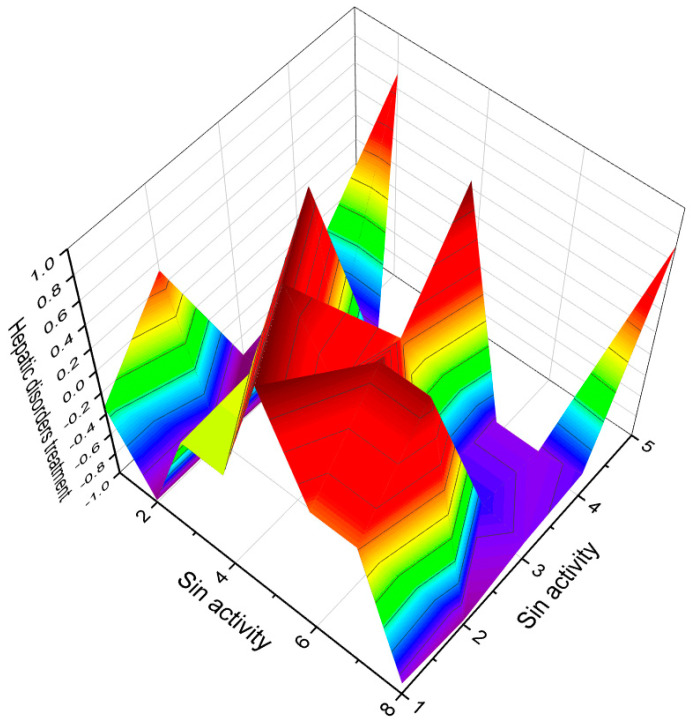
3D graph depicting the predicted and calculated activity as angiogenesis inhibitors of steroids bearing the 17,20-epoxy group derived from the marine sponge *Acanthodendrilla* sp. (**91**, **93**, **96**, **99**, **101**–**103**, **107**, and **108**). The graph provides a visual representation of their inhibitory effects on angiogenesis with a high confidence level of over 90%. Angiogenesis inhibitors hold significant potential as unique natural and/or synthetic agents in the fight against cancer as they target the growth of blood vessels that support tumor growth rather than directly affecting tumor cells themselves.

Steroidal sulfates known as acanthosterol sulfates A−J (**91**−**100**), whose structures can be found in Figure 22, have been extracted from a Japanese marine sponge called *Acanthodendrilla* sp. The antifungal activity of acanthosterol sulfates I and J (**99** and **100**) against the yeast *Saccharomyces cerevisiae* A364A has been demonstrated [80]. In addition, a steroidal sulfate named acanthosterol A (**101**) has been isolated from the same marine sponge, along with nine other acanthosterol sulfates B–J (**102**–**111**), whose structures are presented in Figure 22. The antifungal activity of acanthosterol sulfates I and J (**110** and **111**) against the yeast *S. cerevisiae* A364A has also been observed [80]. A three-dimensional graph depicting the structure of acanthosterol A (**101**) can be found in Figure 23. Further details on the activity of the acanthosterol sulfates are provided in Table 8.

Starfish, fascinating organisms of the marine world, possess a remarkable ability to produce a vast array of biologically active metabolites [15,22]. These metabolites have garnered significant interest in the field of medicine due to their diverse pharmacological properties and potential applications in practical healthcare. Starfish-derived metabolites have been studied extensively, revealing promising therapeutic potential in various areas of medicine [22]. They have exhibited antimicrobial properties, making them potential candidates for the development of novel antibiotics or antimicrobial agents. Additionally, certain starfish metabolites have demonstrated anti-inflammatory effects, suggesting their potential use in treating inflammatory conditions. Furthermore, starfish-derived compounds have shown promise as anticancer agents, with some exhibiting cytotoxic and apoptosis-inducing effects on cancer cells [15,22,49].

These compounds have the potential to contribute to the development of innovative cancer therapies or serve as leads for drug discovery. Moreover, starfish metabolites have displayed activities such as antiviral, antifungal, antioxidant, and immunomodulatory effects. These properties make them attractive candidates for addressing various diseases and conditions, including viral infections, fungal diseases, oxidative stress-related disorders, and immune-related disorders. The exploration of starfish metabolites continues to unveil their diverse and valuable pharmacological activities. Ongoing research aims to further elucidate their mechanisms of action, optimize their therapeutic potential, and explore their applications in practical medicine. The unique bioactive compounds derived from starfish offer promising prospects for the development of novel drugs and therapeutic interventions that can positively impact human health [15,22,81,82,83,84].

**Figure 24:**
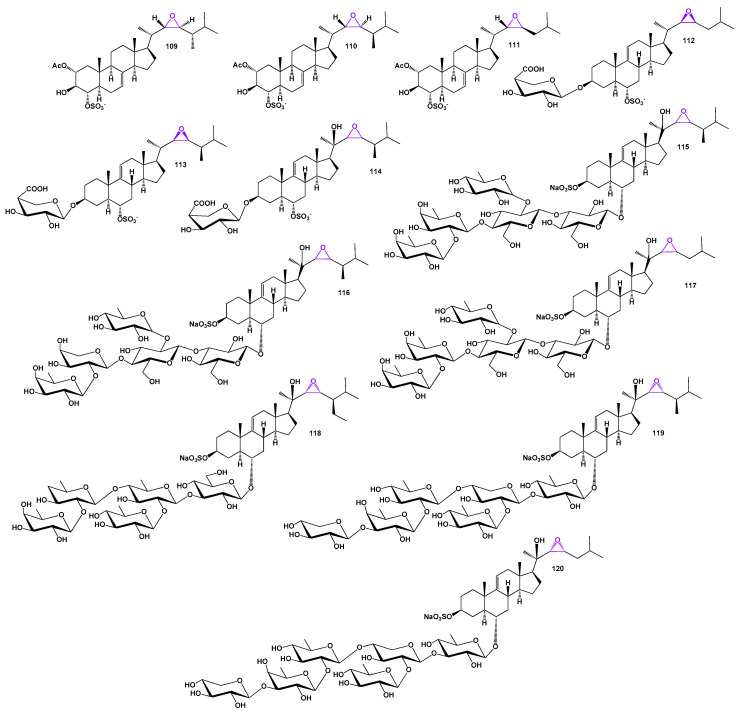
Steroids, and steroid glycosides bearing a 22,23-epoxy group.

22,23-Epoxy steroid glycosides, namely downeyosides C (**112**), D (**113**), and E (**114**), have been obtained from extracts of the starfish *Henricia downeyae* found in the Gulf of Mexico [81]. Mild cytotoxic asterosaponins (**115**), (**116**), and (**117**) have been isolated from the cushion star *Culcita novaeguineae* [82]. Archasteroside A (**118**), isolated from the Vietnamese starfish *Archaster typicus*, exhibited moderate cytotoxic activities against HeLa and mouse JB6 P(+) Cl41 cell lines [83]. Furthermore, the Far East starfish *Hippasteria kurilensis* from the Sea of Okhotsk yielded hexaosides 22,23-epoxy steroid glycosides known as hippasterioside A and B (**119** and **120**; the structures are presented in Figure 24) [84]. These compounds were found to inhibit the colony formation of human HT-29 colon cancer cells.

## 8. Miscellaneous Steroids with α,β-Epoxy Group Derived from Different Sources

After conducting an analysis of published articles on α,β-epoxides, it has been observed that the 1,2-epoxy group is not present in natural sterols and related metabolites [2,9,10]. Additionally, the 2,3-epoxy group is exceptionally rare in natural steroids and isoprenoid lipids. Therefore, the category of miscellaneous steroids has been assigned to the group containing the 2,3-epoxy functionality, along with other epoxy groups that will be discussed.

**Figure 25:**
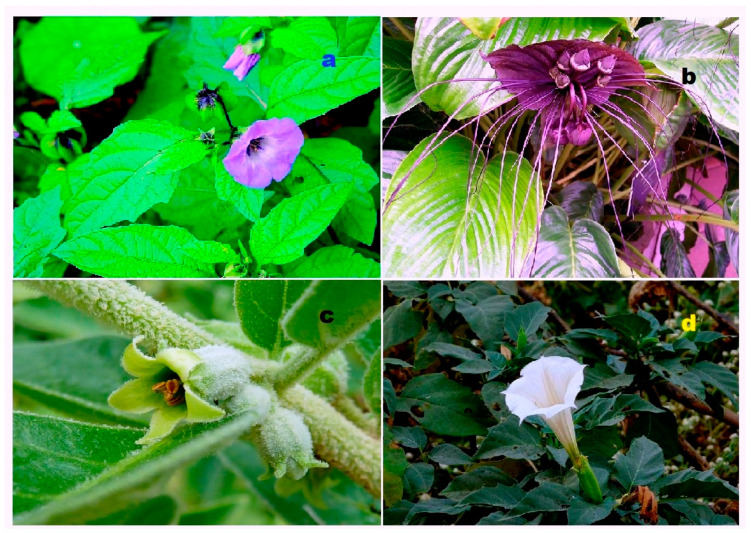
In addition to their presence in marine organisms, rare and unusual steroids have also been discovered in various plant extracts. These unique steroids offer intriguing insights into the diverse chemical profiles found in the plant kingdom. Following are some notable examples: In *Nicandra physaloides* (**a**), steroids (**122**, **123**, and **124**) have been identified. These compounds contribute to the complex chemical composition of this plant species, potentially imparting it with distinct biological activities. *Tacca subflabellata* (**b**) is another plant that harbors intriguing steroids. Steroids (**125** and **126**) have been isolated from this species, further adding to its chemical diversity. The discovery of these steroids in *Tacca subflabellata* highlights the potential pharmacological significance of this plant. *Withania coagulans* (**c**) yields an interesting steroid (**127**) upon isolation. This finding underscores the potential bioactive properties associated with this plant and the unique chemical constituents it possesses. *Datura inoxia* (**d**) is another plant known for its diverse chemical composition. Within its aerial parts, steroids (**139**) and (**140**) have been identified. These compounds contribute to the overall chemical complexity of *Datura inoxia* and may offer valuable pharmacological properties.

**Figure 26:**
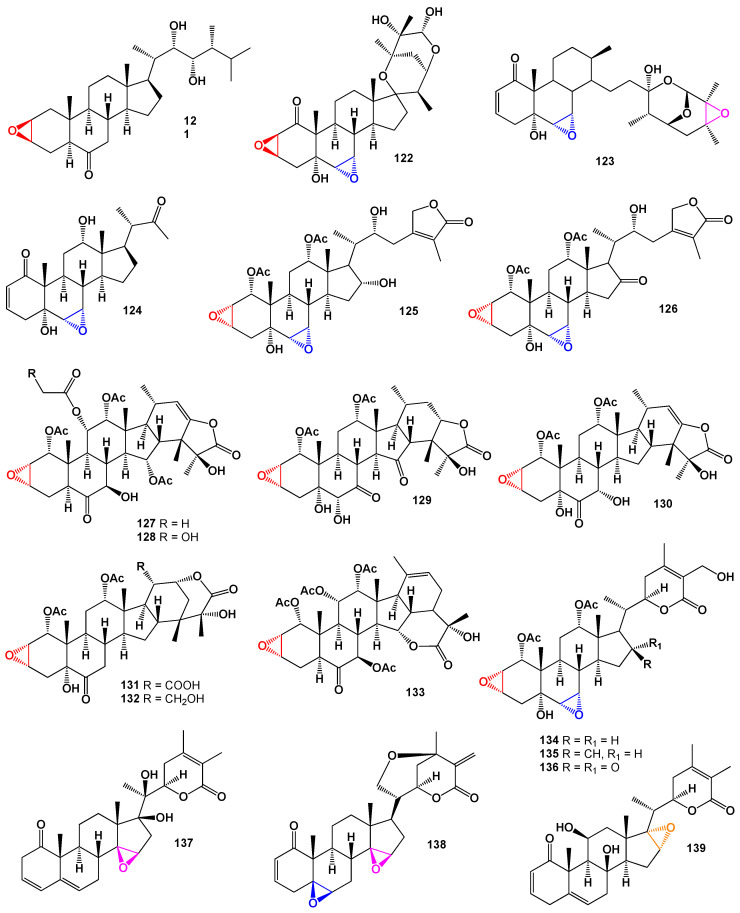
Miscellaneous steroids and isoprenoid lipids. epoxy groups in different positions have different colors.

In 1995, a steroid bearing a 2,3-epoxy group was first discovered in the seeds of *Secale cereale* (rye), and it was identified as the brassinosteroid called secasterone. The compound is known as (22*R*,23*R*,24*S*)-22,23-dihydroxy-2β,3β-epoxy-24-methyl-5α-cholestan-6-one (**121**) [85]. Furthermore, a study involving the examination of an ethyl acetate extract obtained from the calyces of *Nicandra physaloides* resulted in the isolation of three withanolides (a depiction of the plant sample can be seen in Figure 25). These withanolides have been designated as nicphysatones A, B, and C [86]. Through chemical analysis, it has been determined that nicphysatone C (**122**) possesses a 2,3-epoxy group, while both nicphysatones A (**123**) and B (**124**) contain a 6,7-epoxy group. Withanolides, which are structurally related to these compounds, often exhibit variations of the γ-lactone moiety. For instance, taccalonolides O (**125**) and P (**126**; the structure is shown in Figure 26) have been discovered in lipid extracts obtained from the rhizomes and tubers of *Tacca subflabellata* [87,88].

The genus *Tacca* comprises flowering plants that belong to the order Dioscoreales. These plants are primarily found in tropical regions of South America, Africa, Australia, Southeast Asia, and various oceanic islands. Within this genus, there are several species that contain a diverse range of highly oxygenated ixocarpalactone-type withanolides [89,90,91,92,93,94,95]. One notable example of these withanolides is taccalonolide A (**127**) along with its analog taccalonolide L (**128**) [89]. The chemical structure of taccalonolide L can be seen in Figure 26, and its biological activity is detailed in Table 9. Extracts from *Tacca plantaginea T. paxiana T. subflabellata*, and *T. plantaginea* have been found to contain over 20 different withanolides, including compounds such as **128**, **129**, taccalonolide M, **130**, taccalonolide G, **131**, taccalonolide H, **132**, taccalonolide Q, and **133**, taccalonolide Y [90,91,92,93,94].

**Figure 27:**
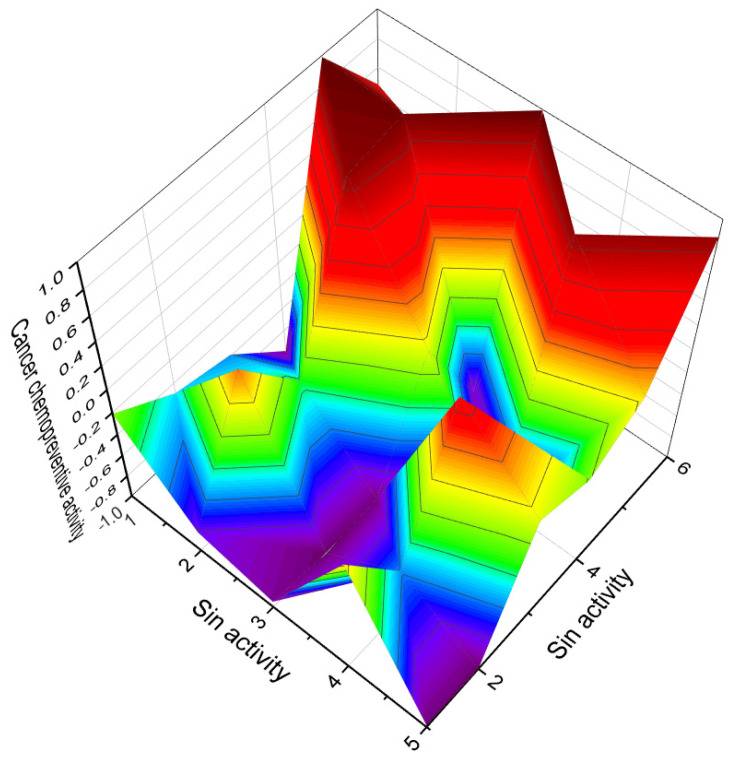
3D graph illustrating the predicted and calculated chemopreventive activity of miscellaneous steroids and isoprenoid lipids derived from plant species (**131**, **134**–**136**, and **138**). This graph provides valuable insights into their potential as chemopreventive agents, demonstrating their inhibitory effects on the development and progression of malignant neoplasms. The predicted and calculated chemopreventive activity values have been determined with a high confidence level of over 90%. Chemopreventive activity, a modern and advanced approach in the treatment of various types of malignant neoplasms, involves the introduction of specialized chemicals or drugs known as antitumor (antineoplastic) chemotherapeutic agents into the human body. These agents play a crucial role in combating cancer by preventing its occurrence, inhibiting its growth, and reducing the risk of tumor progression. The exploration of miscellaneous steroids and isoprenoid lipids derived from plant species offers great potential in the development of novel chemopreventive strategies. These natural compounds exhibit diverse chemical structures and unique properties that can target specific molecular pathways involved in carcinogenesis. By analyzing the predicted and calculated chemopreventive activity of these compounds through the 3D graph, we gain valuable insights into their effectiveness and potential applications in preventing malignant neoplasms. This information contributes to the ongoing efforts in developing advanced chemopreventive approaches and identifying promising candidates for further research and development in the field of oncology. The utilization of chemopreventive agents is a crucial aspect of modern cancer treatment, and this 3D graph serves as a valuable tool for assessing the chemopreventive potential of miscellaneous steroids and isoprenoid lipids derived from plant species.

*Tacca plantaginea* has specifically been found to contain three withanolides named plantagiolides A–D (**134**–**136**) [95]. Additionally, another withanolide called 14,15β-epoxywithanolide I (**137**), has been isolated from *Withania coagulans* [96]. A similar steroidal lactone (**138**), with a 3D graph shown in Figure 27, was originally isolated from *W. adpressa* [97] and subsequently identified as a new compound from *W. coagulans* [98]. Furthermore, the lactones daturalicin (**139**) and physagulin H (**140**) have been discovered in the aerial parts of *Datura inoxia* [99] and *Physalis angulata* [100,101,102], respectively. These compounds share a common feature of having a 14,15-epoxy group. In summary, the *Tacca* genus encompasses flowering plants found in tropical regions worldwide. These plants contain a variety of highly oxygenated ixocarpalactone-type withanolides, including taccalonolide A and its analog taccalonolide L. Other related withanolides have been identified in different species of *Tacca*, along with additional withanolides found in *Withania coagulans*, *W. adpressa*,* Datura inoxia*, and *Physalis ngulate*.

**Figure 28:**
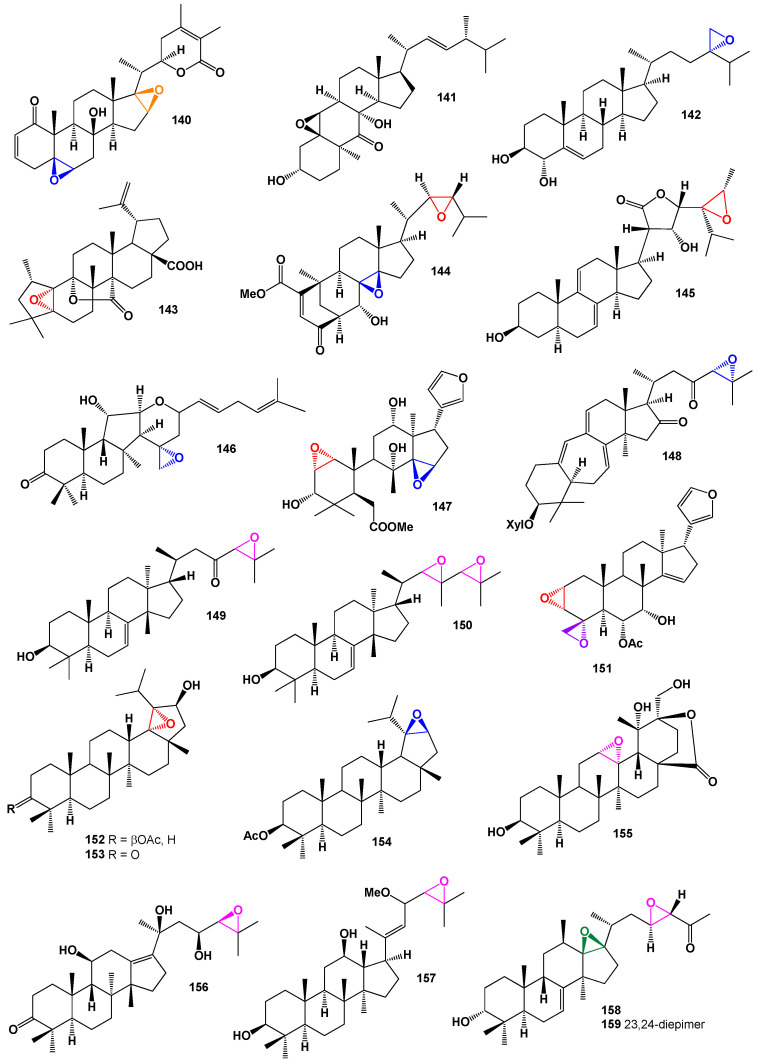
Miscellaneous steroids and isoprenoid lipids. epoxy groups in different positions have different colors.

Pleurocin A (**141**), an abeo-ergostane-type steroid, was isolated from the fruiting bodies of *Pleurotus eryngii* (Pleurotaceae). Its chemical structure can be seen in Figure 28, and its biological activity is detailed in Table 10. Pleurocin A exhibited inhibitory activities against NO production without significant cytotoxicity at concentrations lower than 30 μM [103]. Another compound, 24(*S*),28-epoxyergost-5-ene-3β,4α-diol (**142**), displayed cytotoxicity against the acute leukemia (HL60) cell line, with an IC_50_ value of 33.5 µM. It also showed activity against the hepatoma cancer (HepG2) and colon adenocarcinoma (SW480) cell lines, with IC_50_ values of 64.3 and 71 µM, respectively [104]. Breynceanothanolic acid (**143**), an unusual triterpenoid derivative of 25-nor-ceanothic acid, was discovered in grated roots of *Breynia fruticose* [105].

**Figure 29:**
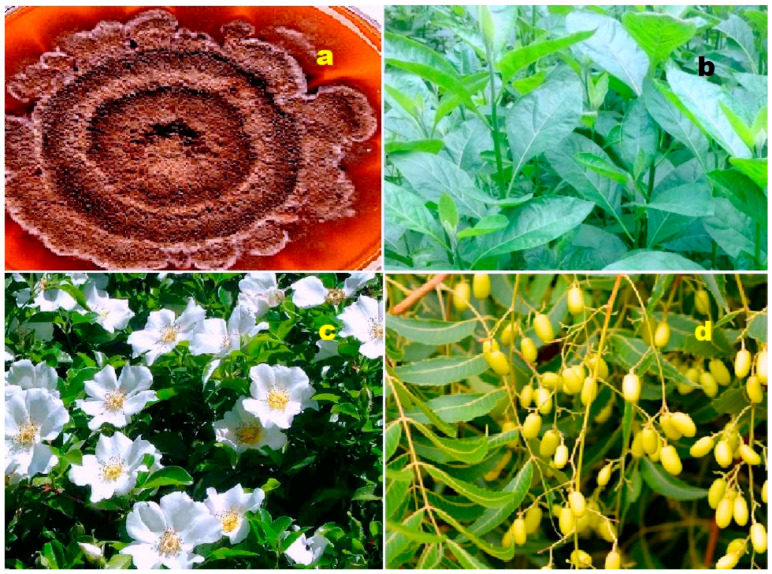
Fungal and plant species produce unusual steroids. Steroid (**72**) is produced by fungus *Phomopsis* sp. (**a**); steroid (**145**) was isolated from the leaves of *Vernonia amygdalina* (**b**). *V. amygdalina* is a perennial herb and extracts have been used in various folk medicines as remedies for protozoal and bacterial infections. In addition, extracts are used to treat diseases such as colds, bronchitis, and liver diseases. Steroid (**147**) was found in the leaves of *Rosa laevigata* (**c**). *R. laevigata* or the Cherokee rose, is widely known in ethnic medicine as it has strong biological activity, and it is used in the traditional medicine system to treat diabetes, nephropathy, myocardial damage, oxidative damage, and liver damage. Plant extracts have antioxidant, anti-inflammatory, antiviral and antitumor activity, as well as kidney-protective, immunomodulatory, lipid-lowering, cardiovascular, bacteriostatic, and other pharmacological effects. Two steroids (**149** and **150**) were isolated from the branches and leaves of *Azadirachta indica* (**d**). The plant *A. indica* has long been used in Ayurvedic and folk medicine and is used in cosmetics and in organic farming applications. The leaves have long been used as a traditional remedy for diabetes and can help control blood sugar levels. Extracts from all parts of the tree are commonly used in shampoos to treat dandruff, as well as in soaps or creams for skin conditions such as acne, psoriasis, and athlete’s foot.

Phomopsterone A (**144**), an ergostane-type steroid, was isolated from the plant-derived fungus *Phomopsis* sp. TJ507A. This compound is an unprecedented ergosteroid that features a rearranged bicyclo[3.3.1]nonane motif resulting from B-ring scission and a subsequent 180° rotation of the ring A during biosynthesis [106]. *Vernonia amygdalina*, a plant species, yielded an unusual epoxide called (23*S*,24*R*,28*S*)-3β,22α-dihydroxy-7,8,9,11-tetra-dehydro-24,28-epoxy-5α-stigmastane-21,23-carbolactone (**145**) [107]. The plant sample can be seen in Figure 29. Rhabdaprovidine G (**146**), a rare epoxide 6,6,5-tricyclic terpenoid, was isolated from the Vietnamese sponge *Rhabdastrella providentiae*. This compound exhibits a novel structure with five rings and nine chiral carbon centers in the iso-malabaricane triterpene backbone [108].

*Rosa laevigata*, also known as Cherokee Rose, contains a rare 1,2-epoxy group oleanane derivative named 2α,3α,19α,23-tetrahydroxyolean-12-en-28-oic acid (**147**) in its leaves. This tree species is native to southern China and Taiwan, and it is invasive in the United States [109]. *Actaea racemosa*, commonly known as black cohosh, black bugbane, black snakeroot, or rattle-top, is a flowering plant in the buttercup family. Its leaves contain the unusual triterpene xyloside cimipodocarpaside (**148**) [110].

**Figure 30:**
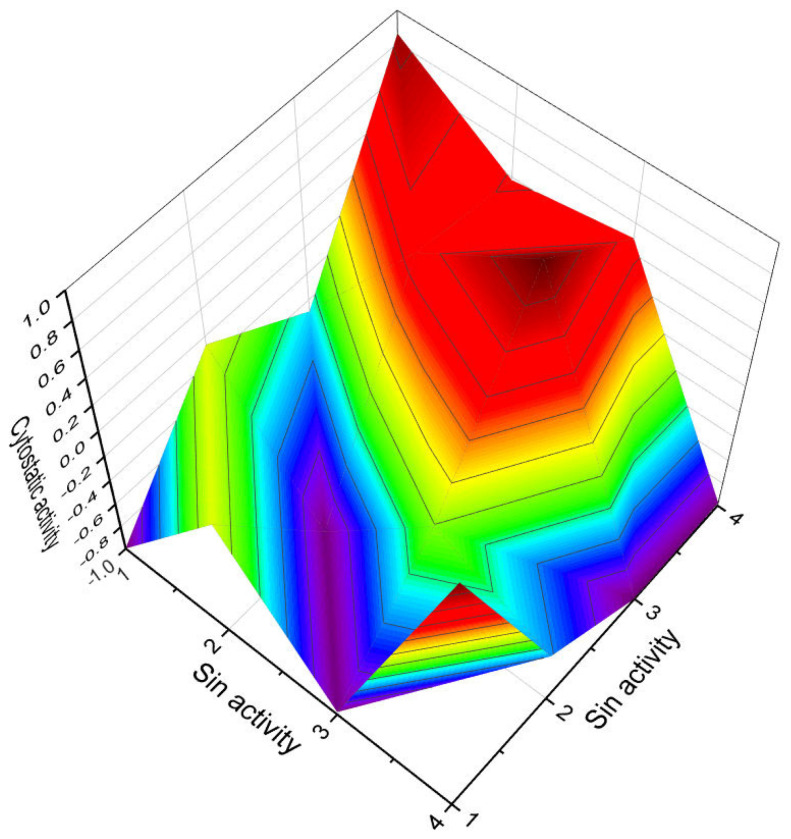
3D graph showcasing the predicted and calculated cytostatic activity of miscellaneous steroids and isoprenoid lipids (**152**–**155**). This graph provides valuable insights into the potential cytostatic effects of these compounds with a high confidence level of over 91%. Cytostatic activity plays a vital role in cancer treatment as it refers to the ability of a substance to inhibit or slow down the growth and division of cells, particularly cancer cells. Cytostatic agents act by disrupting key cellular processes involved in cell cycle progression, effectively arresting cell growth and proliferation. Unlike cytotoxic agents that induce cell death, cytostatic agents offer the advantage of controlling the growth and spread of cancer cells without causing immediate cell death. The miscellaneous steroids and isoprenoid lipids under investigation hold promise as potential cytostatic agents.

From the branches and leaves of *Azadirachta indica*, two tirucallane triterpenoids were isolated: 24,25-epoxy-3β-hydroxy-20-oxo-7-tirucallene (**149**) and 22,23;24,25-diepoxy-3β-hydroxy-7-tirucallene (**150**) [111]. The bark of *Chisocheton ceramics* yielded a rare limonoid called ceramicine E (**151**). This compound exhibits inhibition of cell growth on various cell lines, including HL-60, A549, MCF7, and HCT116 [112]. Extracts from *Taraxacum officinale* contained several lupane and ursane triterpenoids (**152**–**155**). The 3D graph of these compounds can be seen in Figure 30. Additionally, the leaves of *Rehmannia glutinosa* also yielded lupane and ursane triterpenoids [113,114].

**Figure 31:**
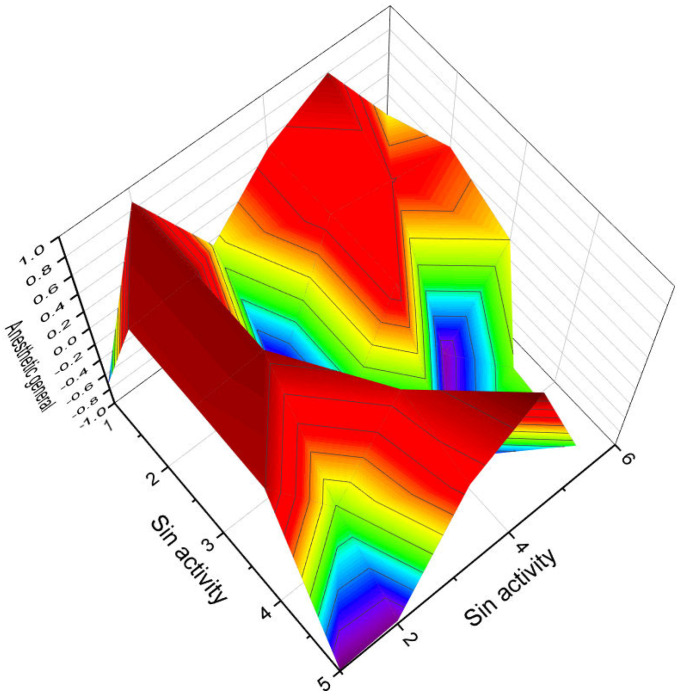
3D graph showcasing the predicted and calculated sedative-hypnotic properties, specifically related to general anesthesia, of miscellaneous steroids and isoprenoid lipids (**145**, **150**, **156**–**159**). This graph provides a comprehensive overview of their potential sedative-hypnotic effects with a high confidence level of over 90%. General anesthesia refers to the artificially induced reversible state resembling deep sleep where pain sensations are suppressed, consciousness is suspended, and muscle relaxation is achieved. In this context, the sedative-hypnotic properties of certain steroids and isoprenoid lipids have been observed with a significant degree of certainty. It is interesting to note that these compounds exhibit distinct characteristics from well-known drugs such as ketamine, propofol, and etomidate, offering potential alternative options in the field of general anesthesia. Red—strong, Blue—poor activity.

**Figure 32:**
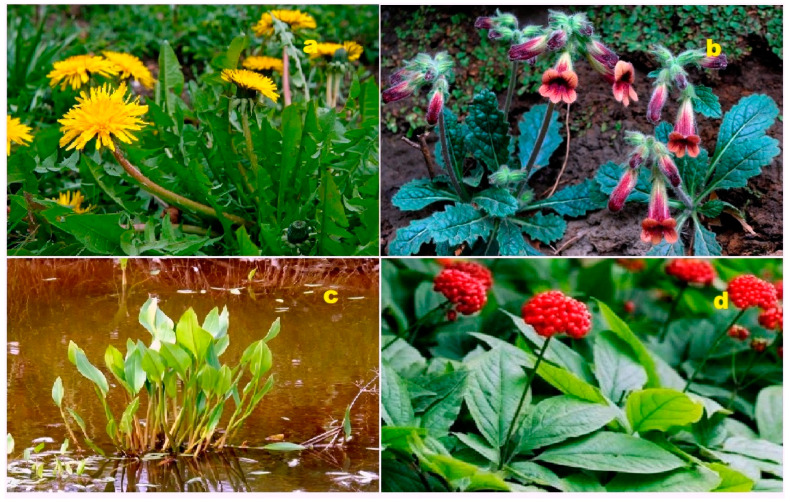
Steroids and isoprenoid lipids (**152**–**155**) were found in *Taraxacum officinale* (**a**) and isolated from the leaves of *Rehmannia glutinosa* (**b**). The young leaves of *T. officinale*, or common dandelion, are used as food in salads, drinks, and vegetable dishes for their nutritional value. This plant is a promising source for the prevention and treatment of diseases and extracts show hepatoprotective, antioxidant, and anticancer activities. Dandelion’s anti-diabetic properties are attributed to bioactive chemical constituents such as high levels of fiber, minerals, vitamins, essential fatty acids, and bioactive steroids. Rehmannia (or *Rehmannia glutinosa*) is a plant that grows mainly in China. The extracts are commonly used in combination with other herbs in traditional Chinese medicine and are used for strengthening the immune and nervous systems and can also reduce pain and swelling. Rehmannia increases renal blood flow and stimulates the production of the renal hormone erythropoietin. Steroid (**156**) was found in the rhizomes of *Alisma orientale* (**c**), and triterpenoid (**157**) is found in Korean red tea (*Panax ginseng*) (**d**).

A protostane derivative called 20-hydroxyalisol C (**156**) was isolated from the rhizomes of *Alisma orientale*. The 3D graph of this compound can be seen in Figure 31, and its biological activity is detailed in Table 11. 20-Hydroxyalisol C exhibits inhibitory effects on human carboxylesterase 2, making it of great medical interest [115]. The plant sample is depicted in Figure 32. In traditional Korean red tea, a triterpenoid (**157**) has been found. This tea, which is made from steamed ginseng, was evaluated for its protective effects against melanogenesis. The compound has shown potent inhibitory effects on both melanin synthesis and tyrosinase activity [116].

**Figure 33:**
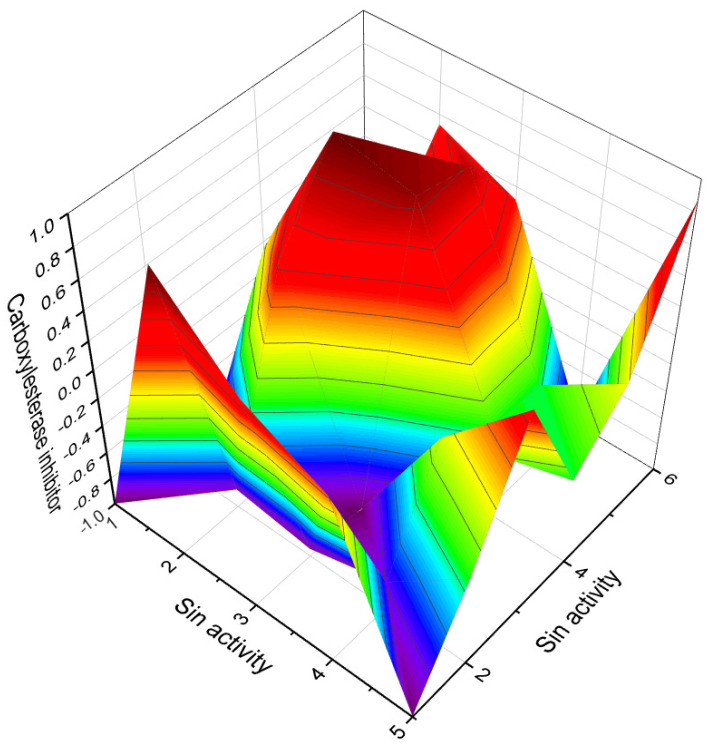
The 3D graph shown the predicted and calculated activity of miscellaneous steroids (**160**, **162**–**165**) as carboxylesterase inhibitors with over 90% confidence. Carboxylesterase inhibitors are substances that inhibit the activity of carboxylesterase enzymes. Carboxylesterases are a group of enzymes involved in the metabolism and breakdown of ester compounds in the body. They play a crucial role in the detoxification and elimination of various drugs, xenobiotics, and endogenous compounds. Carboxylesterase inhibitors can interfere with the function of these enzymes either by binding to the active site and blocking substrate binding or by altering the enzyme’s conformation or activity.

A series of protostane triterpenes (**158**–**167**), which are tetracyclic triterpenes, were isolated from the rhizome of *Alisma orientale*. These compounds exhibited moderate inhibitory activities, particularly towards hCE-2 enzymes. Several metabolites were identified, including 13β,17β-epoxyalisol A (**160**), 13β,17β-epoxyalisol A 24-acetate (**161**), 11-deoxy-13β,17β-epoxyalisol A (**162**), (13β,17β-epoxyalisol B 23-acetate) (**163**), 13β,17β-epoxyalisol B (**164**), and 11-deoxy-13β,17β-epoxyalisol B 23-acetate (**165**). The 3D graph of compound **165** can be seen in Figure 33. Additionally, alisol K 23-acetate (**166**) and 16β,23β-oxidoalisol B (**167**) were also identified. Many of these compounds contained one or two epoxy groups [117,118,119,120,121,122].

The methanol extract of the marine sponge *Theonella swinhoei* yielded a polyhydroxylated steroid known as theonellasterol I (**168**) [123]. The garden fungi *Ganoderma australe* produced a secosterol called australic acid (**169**), which exhibits inhibition of cancer cell growth through the activation of apoptosis [124]. Additionally, elfvingic acid methyl ester (**170**), isolated from the fruit body of the fungus *Elfvingia applanata*, demonstrated strong cytotoxicity against Kato III and Ehlrich cells [125].

*Centaurea chilensis*, a plant belonging to the Asteraceae family, contained 3β-Acetoxy-17β,21β-epoxyhopane (**171**). *Adiantum caudatum* yielded a similar metabolite, 17β,21β-epoxyhopane (**172**). Furthermore, extracts from various plants, including *Adiantum capillus-veneris*, *A. monochlamys*, *A. cuneatum*, *A. pedatum*, and *A. emarginatum*, contained a rare epoxide named isoadiantol B (**173**) [126,127].

**Figure 34:**
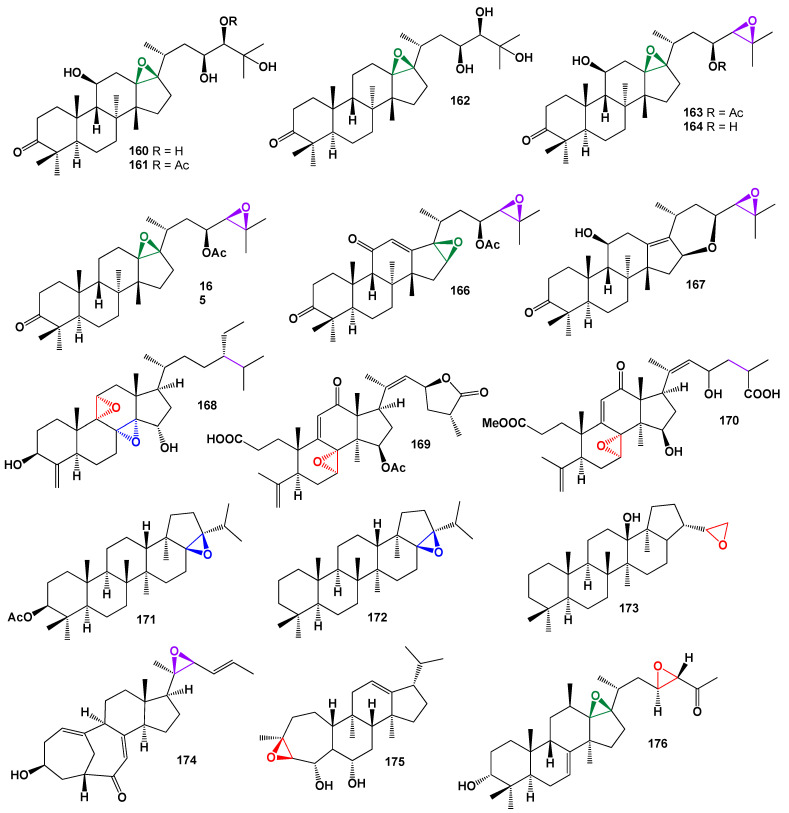
Miscellaneous steroids and isoprenoid lipids.

**Figure 35:**
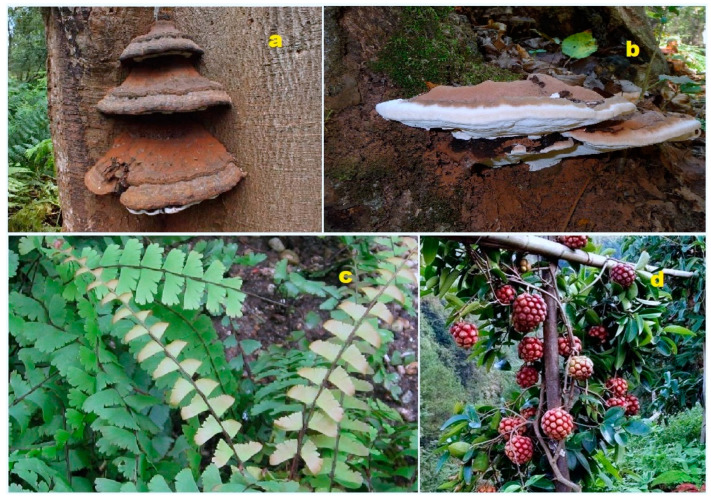
Several specimens of fungi and plants contain bioactive steroids. The fungi *Ganoderma australe* (**a**) and *Elfvingia applanata* (**b**) produced australic acids (**169**) and elfvingic acid methyl ester (**170**), respectively. The inedible fungus *Ganoderma australe* (or *Ganoderma adspersum*) is a common perennial tinder fungus that causes white core rot in trees from the genera Tilia (linden), Quercus (oaks), Fagus (beech or birch), Platanus (Sycamore), and Aesculus (horse chestnut and relatives). *Elfvingia applanata* is a medicinal mushroom that has been used to treat cancer of the esophagus and stomach and is also known to have an inhibitory effect on hepatitis B virus infection. Rare triterpenoids: 17β,21β-epoxyhopane (**172**) was detected in *Adiantum caudatum* (**c**) and kadcoccinone D (**176**) was found in *Kadsura coccinea* (**d**). The fern *Adiantum caudatum*, commonly walking maidenhair, tailed maidenhair, and trailing maidenhair, is commonly found in the southeast countries of Bangladesh, Burma, India, Nepal, Philippines, Thailand, China, and Vietnam. Known as “Hansraj” in the *Ayurvedic System of Medicine*, its extracts have been used for colds, tumors of the spleen, liver, and other internal organs, skin diseases, bronchitis, and inflammatory diseases. It is also considered a tonic and diuretic. *Kadsura coccinea* is an evergreen climbing plant with woody stems that is used as food and medicine. In traditional Chinese medicine, it is used to treat rheumatoid arthritis and gastrointestinal disorders.

The volcanic ash-derived fungus *Penicillium citrinum* HGY1-5 produced an unusual steroid named precyclocitrinol B (**174**) [128]. A cultured marine-derived fungus (strain CNM-713), identified as an undescribed member of the genus *Aspergillus*, yielded a sesterterpene epoxide-diol called aspergilloxide (**175**) [129]. *Kadsura coccinea* provided a lanostane-related triterpenoid named kadcoccinone D (**176**) [130]. The structure of compound **175** can be seen in Figure 34, and its biological activity is detailed in Table 12. The plant sample is depicted in Figure 35.

The fungal kingdom is a unique biological association of living organisms that produces a wide variety of metabolites, many of which exhibit diverse biological activities that are beneficial to human health [131,132,133,134]. Fungi are known to produce steroids and isoprenoid lipids, which can possess both toxic and valuable biological activities [134,135,136,137]. It is worth noting that fungal endophytes, which widely inhabit plants [138,139,140], are likely responsible for the synthesis of these steroids and isoprenoid lipids. Understanding the role of fungal endophytes in their production is crucial. The following data present the structures of steroids and meroterpenoids produced by fungi and fungal endophytes along with their biological activities. For instance, the fungus *Stereum hirsutum*, also known as false turkey tail and hairy curtain crust, was found to parasitize another fungus, *Tremella aurantia*. This fungus produced a steroid featuring a bicyclo[3.3.1]nonane motif (**177**). This steroid exhibited cytotoxic activity against several cancer cell lines, including A549, HL-60, MCF-7, SMMC-7721, and SW480 [141].

Progressive degradation of ergostane steroids through 5,6- and 9,10-oxidative cleavage leads to the formation of highly cleaved sterols, such as steroid residues (**178**). These steroid residues were discovered in the fungus *Hericium alpestre* and exhibited cytotoxic activity against the lung cancer cell line A549 [142]. *Aspergillus flocculosus* PT05-1, a cultivated fungus in a hypersaline medium, produced the epoxide (**179**). This compound displayed moderate antibacterial and antifungal activity, as well as weak cytotoxicity against the cancer cell lines HL-60 and BEL-7402 [143]. A bioactive steroid (**180**), known for its activity against the human immunodeficiency virus and inhibition of nitric oxide production, is produced by the endophytic fungus *Trichoderma* sp. [144]. From the cultures of the basidiomycete *Favolaschia calocera* BCC 36684, two bis-epoxides named favolon (**181**) and favolon C (**182**) were isolated. These compounds demonstrated antifungal activity [145]. The fungus *Aspergillus flocculosus* 16D-1 produces a bioactive meroterpenoid called asperflotone (**183**), which is an 8(14→15)-abeo-steroid. Asperflotone exhibited inhibitory effects on IL-6 secretion [146].

Pleurocin B (**184**) and matsutakone (**185**), two steroids with a rearranged ring B, were isolated from the fruiting bodies of *Pleurotus eryngii*. Both metabolites showed stronger inhibitory activity on nitric oxide production compared to a nitric oxide synthase [147]. Penicillitone (**186**), an unusual 15(14→11)-abeo-ergostane, was isolated from the culture of the fungus *Penicillium purpurogenum* SC0070. This compound displayed strong cytotoxic activity against the cancer cell lines A549, HepG2, and MCF-7 [148]. A series of steroids with an unprecedented steroid skeleton (**187**–**190**), named strophasterols A–D, respectively, were isolated from the mushroom *Stropharia rugosoannulata* [149]. Additionally, strophasterol E (**191**) and strophasterol F (**192**), which have a strophastane skeleton, were isolated from the fruiting bodies of *Pleurotus eryngii* [150].

A steroid containing two epoxy groups, (22*E*)-3β-hydroxy-5α,6α,8α,14α-diepoxyergosta-22-en-7-one (**193**), was isolated from the fungal endophyte *Aspergillus awamori*, which was obtained from the soil around the mangrove plant *Acrostichum speciosum* in Hainan, China. This compound displayed mild cytotoxicity towards the lung cancer cell line A549 [151].

*Penicillium expansum* YJ-15, an endophytic fungus of *Aconitum vilmorinianum*, yielded bioactive isoprenoid epoxycyclohexenones named expanstines A–D (**194**–**197**). Notably, compounds **196** and **197** featured an unusual oxetane ring. These fungal compounds exhibited potent cytotoxic activities against several cancer cell lines, including HL-60, SMMC-7721, A549, MCF-7, and SW-480. Additionally, compounds **194**–**197** demonstrated potent inhibitory effects on nitric oxide production (NO). Furthermore, compounds **196** and **197** displayed potent antibacterial activities against *Bacillus subtilis* [152]. A fungicolous isolate of *Hymenopsis* sp. MYC-1703, collected from the Eucalyptus forest, produced a meroterpenoid named hymenopsin A (**198**) [153].

In an extract from the fungus *Stereum hirsutum*, a cytotoxic ergosteroid named steresterone A (**177**) was discovered. This compound demonstrated cytotoxic activity against several cancer cell lines, including A549, HL-60, MCF-7, SMMC-7721, and SW480 [154]. Furthermore, a steroid fragment (**178**) showed cytotoxicity against the human colon adenocarcinoma cell line HT29 and was detected in the mushroom *Hericium alpestre* [155].

**Figure 36:**
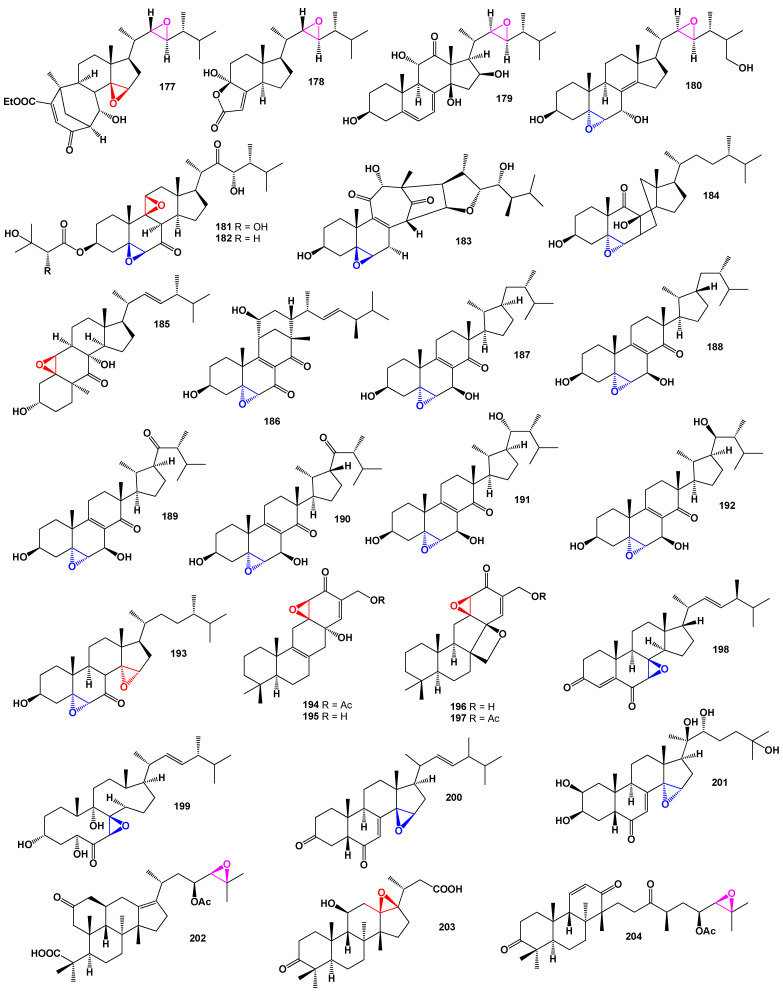
Miscellaneous steroids and isoprenoid lipids derived from fungi.

**Figure 37:**
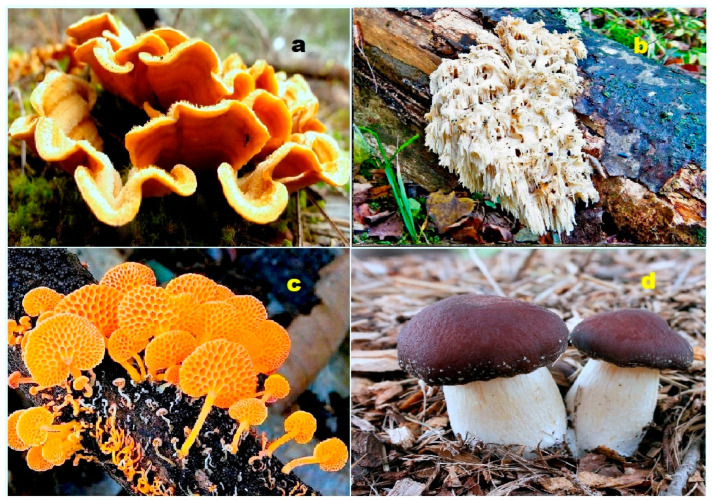
Some mushroom samples contain bioactive steroids. The edible mushroom *Stereum hirsutum* (**a**), also called false turkey tail and hairy curtain crust, contains an uncommon steroid (**187**). The dry extract of this mushroom has significant thrombin inhibitory activity and can be used to make blood-thinning drugs as an alternative to the dangerous drug warfarin. Another edible mushroom called alpine blackberry, *Hericium alpestre* (**b**), contributes to the decomposition of wood and grows on stumps, large fallen trees, and dead spruce and cedar trees. An oxidized fragment of the steroid (**178**) was found in the lipid extract of this mushroom. Its taste is so excellent that French chefs use it to prepare many gourmet dishes. The fungus *Favolaschia calocera* (**c**, or *Orange Poreconch*) is usually found in wood and colonizes along transport routes and may become dominant in other habitats. It has been found on the French islands of Reunion and Maya, which are off the African mainland, as well as in Kenya, Congo, Tanzania, and Zambia. Extracts of this mushroom contain steroids (**181**) and (**182**). Mushroom *Stropharia rugosoannulata* (**d**), commonly known as the Wine Cap Stropharia, “Garden Giant”, burgundy mushroom, or King Stropharia, is found in Europe and North America and was apparently introduced to Australia and New Zealand. In the US, this mushroom is considered edible and is highly valued by connoisseurs. This mushroom contained a series of steroids (**187**–**190**) that are highly biologically active.

A halotolerant fungus, *Aspergillus flocculosus*, produced a 22,23-epoxy steroid (**179**). The structure of compound **179** can be seen in Figure 36, and its biological activity is detailed in Table 12. This compound exhibited moderate antibacterial and antifungal activity and weak cytotoxicity against HL-60 and BEL-7402 cell lines [156]. Additionally, the endophytic fungus *Trichoderma* sp. Yielded a bis-epoxy steroid (**180**) that showed inhibition of nitric oxide production [157]. From the cultures of the basidiomycete *Favolaschia calocera* BCC 36684, two antifungal bis-epoxides named favolon (**181**) and favolon C (**182**) were isolated [158]. A sample of the fungus can be seen in Figure 37.

**Figure 38:**
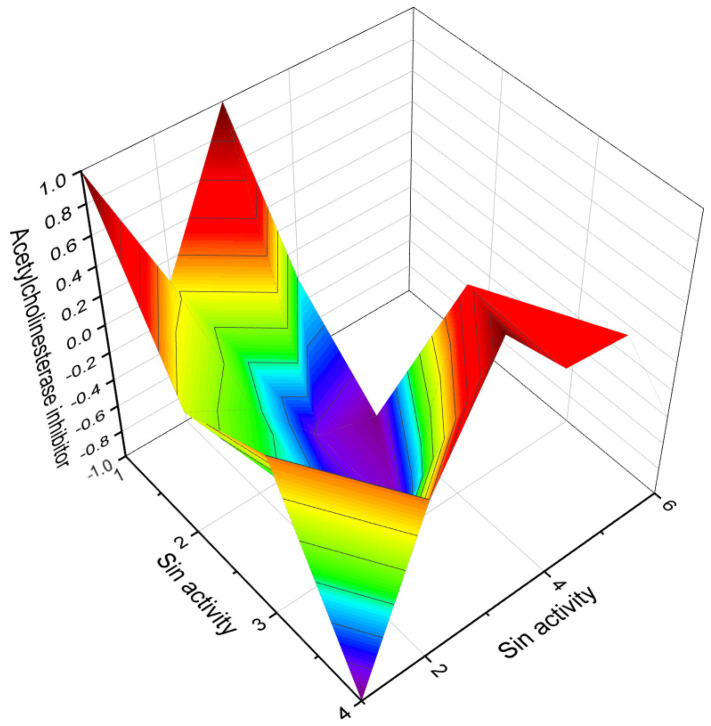
3D graph showcasing the predicted and calculated activity as acetylcholinesterase inhibitors of miscellaneous steroids (**180**, **184**–**186**). This graph provides valuable insights into their potential in inhibiting the activity of acetylcholinesterase, a key enzyme involved in the breakdown of the neurotransmitter acetylcholine. Acetylcholinesterase inhibitors play a crucial role in the management of conditions such as Alzheimer’s disease and other neurodegenerative disorders. By inhibiting the activity of acetylcholinesterase, these compounds help increase the levels of acetylcholine in the brain, leading to enhanced cholinergic neurotransmission and potential improvements in cognitive function. The exploration of miscellaneous steroids as acetylcholinesterase inhibitors offers opportunities for the development of novel therapeutic interventions. By understanding their structure–activity relationship, researchers can potentially optimize their pharmacological properties and enhance their effectiveness as acetylcholinesterase inhibitors.

From the solid culture of *Aspergillus flocculosus* 16D-1, an unusual 8(14→15)-abeo-steroid named asperflotone (**183**) was obtained. This compound exhibited inhibitory effects on IL-6 secretion [159]. Rare steroids with a rearranged ring B, pleurocin B (**184**) and matsutakone (**185**), were isolated from the fruiting bodies of *Pleurotus eryngii*. These compounds showed inhibition of nitric oxide production [160]. Another notable compound, a 15(14→11)-abeo-ergostane named penicillitone (**186**), was found in the culture of the fungus *Penicillium purpurogenum* SC0070 [161]. Penicillitone exhibited anti-inflammatory or antitumor activity. The 3D graph of compound **186** can be seen in Figure 38.

The mushroom *Stropharia rugosoannulata* yielded a series of 15(14→22)-abeo-steroid ergostanes (**187**–**192**) in its extracts [162]. Steroids **187**–**190** were named strophasterols A-D, and two additional compounds, **191** and glaucoposterol A (**192**), were found in the basidiomycete *Cortinarius glaucopus* [163]. Moreover, steroids with a strophastane skeleton, strophasterol E (**191**) and strophasterol F (**192**), were isolated from the fruiting bodies of *Pleurotus eryngii* [164]. A compound named (22E)-3β-hydroxy-5α,6α,8α,14α-diepoxyergosta-22-en-7-one (**193**) was discovered in the fungus *Aspergillus awamori*, which was isolated from the soil around the mangrove plant *Acrostichum speciosum*. This compound exhibited mild cytotoxicity against the lung cancer cell line A549 [165].

The endophytic fungus *Penicillium expansum* YJ-15, which is found in association with the leaves of *Aconite vilmorinianum*, has been found to produce a group of isoprenoid lipids called expanstines A–D (**195**–**197**) [166]. These fungal meroterpenoids have demonstrated potent cytotoxic activities against HL-60, SMMC-7721, A549, MCF-7, and SW-480 cell lines [166]. Another interesting compound, named talarosterone (**198**), was isolated from the fermentation products of the marine sponge-associated fungus *Talaromyces stipitatus* [167]. Talarosterone is a steroid that possesses a 7,8-epoxy group [167]. Additionally, the endophytic fungus *Gibberella zeae* cf-18, which was isolated from the green alga *Codium fragile*, produced a unique steroid known as (22E,24R)-7β,8β-epoxy-3β,5α,9α-trihydroxyergosta-22-en-6-one (**199**) [168]. These discoveries highlight the diverse range of bioactive compounds that can be derived from fungal sources and provide potential avenues for further research and exploration in the field of natural product discovery.

In the methanol extract of the roots of *Serratula wolffii*, two steroids with a 14,15-epoxy group (**200** and **201**) were discovered [169]. Furthermore, within the genus Alisma (Alismataceae), a series of protostane triterpenoids (**195**–**197**) have been identified and reported in various regions worldwide. These triterpenoids exhibit diverse biological activities, including anticancer, lipid-regulating, anti-inflammatory, antibacterial, antiviral, and diuretic effects [170,171]. Over 100 different triterpenoids have been characterized and assigned names such as alisols A-Z, alismanols A-G, and related terpenoids. Figure 36 illustrates the chemical structures of alisol I (**202**), F (**203**), and H (**204**).

**Figure 39:**
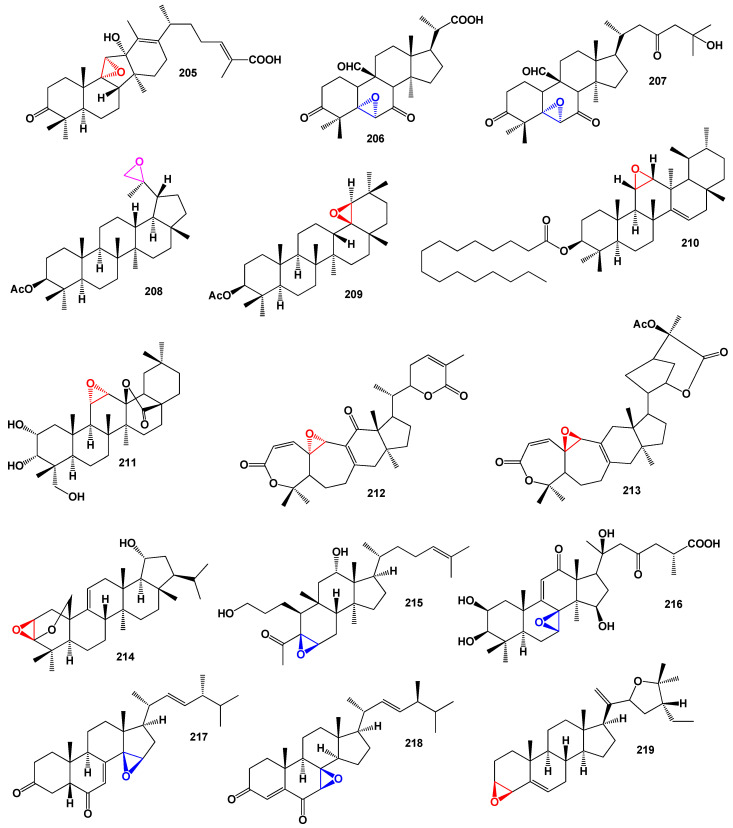
Miscellaneous steroids and isoprenoid lipids derived from fungi and plants.

**Figure 40:**
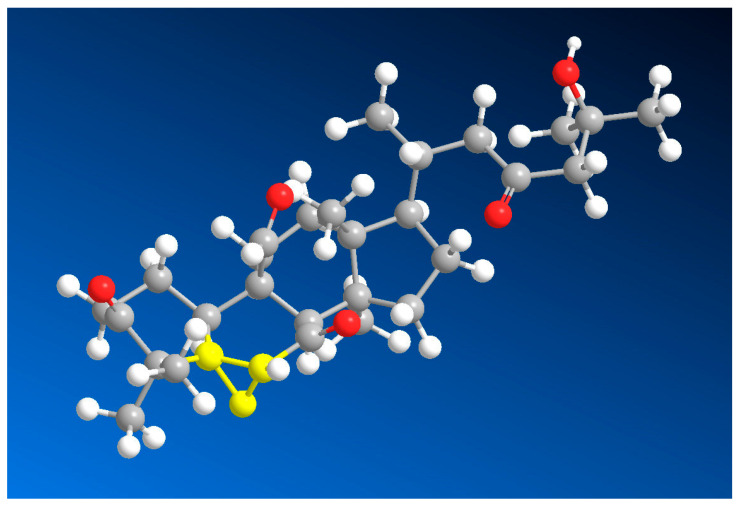
This figure shows a 3D structure of cucurbitane triterpenoid bearing an oxirane ring in the 5,6 position, kuguacin G (**207**), and showing a wide range of antiviral and other biological activities such as *antiviral (HIV)*, 2. *antiviral (influenza A)*, 3. *antiviral (arbovirus)*, 4. *antifungal*, 5. *antibacterial*, and 6, *antiparasitic* activity. The oxirane ring is highlighted in yellow. Gray is carbon, white is hydrogen, and red is oxygen. The percentage of biological activities is shown in Figure 42.

**Figure 41:**
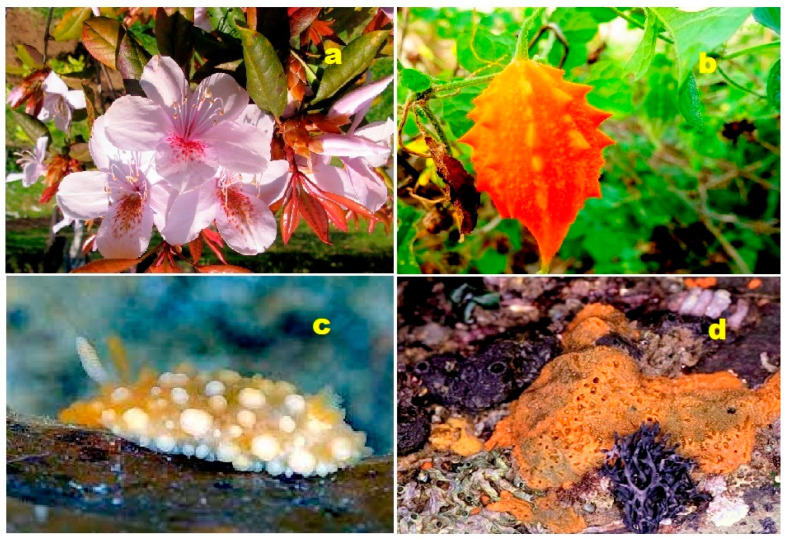
Both terrestrial and marine organisms produce bioactive steroids and triterpenoids. Steroids kuguacins K (**206**) and G (**207**) were found in the vines and leaves of *Momordica charantia* (**a**), and oleanane derivative (**211**) was found in *Rhododendron latoucheae* (**b**). Marine invertebrates are also a source of bioactive metabolites. Thus, lovenone (**215**) was isolated from the nudibranch *Adalaria loveni* (**c**), and the steroid (**217**) is a product of the marine sponge-associated fungus *Gymnasella dankaliensis*, which is a symbiont of the marine sponge *Halichondria japonica* (**d**).

A triterpenoid called kadcoccitone C (**205**) with anti-HIV-1 activity was isolated from *Kadsura coccinea* [172]. The structure of kadcoccitone C can be seen in Figure 39, and its biological activity is detailed in Table 13. From the vines and leaves of *Momordica charantia*, cucurbitane triterpenoids named kuguacins K (**206**) and G (**207**; the 3D model is shown in Figure 40) were discovered to exhibit strong anti-HIV-1 activity, with EC_50_ values of 7.2 and 3.7 µg/mL, respectively [173]. The plant sample is shown in Figure 41, and the percentage distribution of the biological activity of these steroids can be seen in Figure 40. A semi-synthetic lupane triterpenoid (**208**) has demonstrated a wide spectrum of inhibitory activity [174], while an oleanane derivative (**209**) was found to be less active [175].

**Figure 42:**
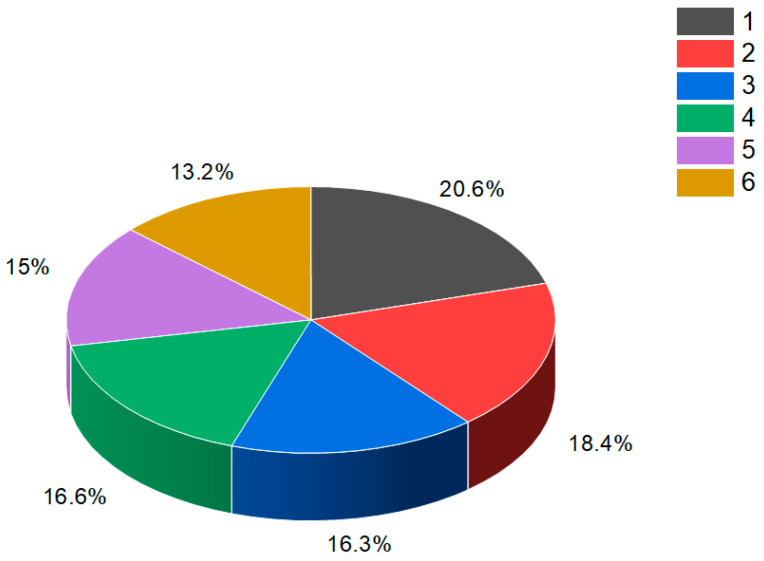
This figure discloses the percentage distribution of biological activities on the example of a cucurbitane triterpenoid, kuguacin G (**207**), from the medicinally important plant *Kadsura coccinea*, which has a wide range of pharmacological properties. Dominant antiviral activities are listed under the following numbers: 1. *antiviral (HIV)* (20.6%), 2. *antiviral (influenza A)* (18.4%), 3. *antiviral (arbovirus)* (16.3%), 4. *antifungal* (16.6%), 5. *antibacterial* (15%), and 6, *antiparasitic* (13.2%). This emphasizes that kuguacin G possesses a wide range of pharmacological properties, indicating its potential as a versatile therapeutic agent.

**Figure 43:**
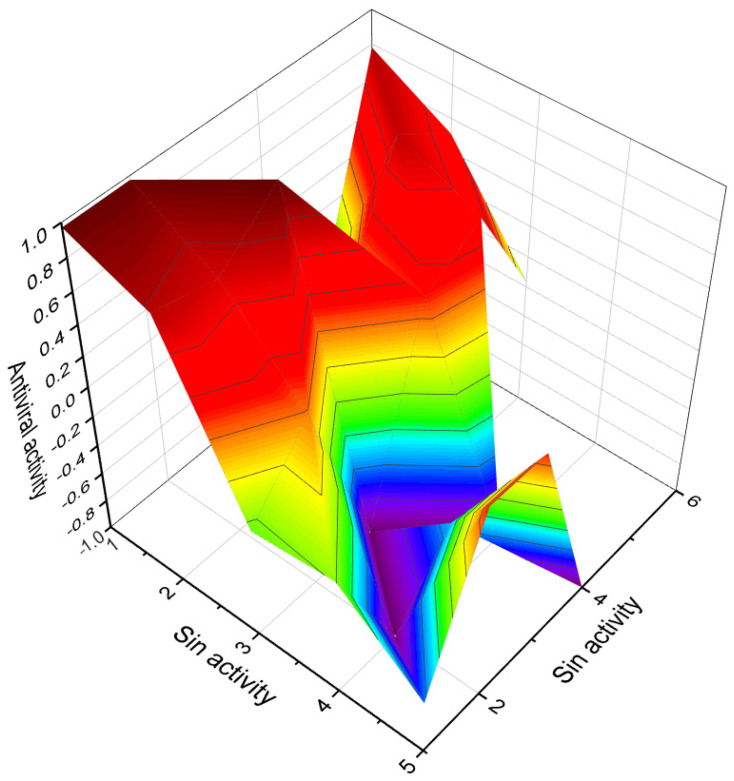
A 3D graph showing the predicted and calculated antiviral activity of miscellaneous steroids and isoprenoid lipids (**206**, **207**, **211**–**213**) with over 94% confidence. Antiviral activity refers to the ability of a substance, such as a drug or natural compound, to inhibit the replication or activity of viruses. It measures the effectiveness of a compound in preventing viral infection or reducing viral replication in infected cells. Antiviral activity can be assessed through various methods, including in vitro assays using cell cultures infected with specific viruses or in vivo studies using animal models. The activity is typically quantified by measuring parameters such as viral load, viral replication, or the inhibition of viral-induced cytopathic effects. Antiviral compounds can target different stages of the viral life cycle, including viral entry into host cells, viral replication, assembly, and release. They may interfere with viral enzymes, inhibit viral attachment to host cells, disrupt viral protein synthesis, or modulate host immune responses to combat viral infections.

Pentacyclic triterpene D-friedours-14-en-11α,12α-epoxy-3β-yl palmitate (**210**) was identified in *Ecdysanthera rosea* [176], and another oleanane derivative (**211**) from *Rhododendron latoucheae* exhibited strong inhibition against HSV-1 virus [177]. A bioactive compound named schisphendilactone B (**212**), obtained from the stems of *Schisandra sphenanthera*, showed promising anti-HIV-1 activity [178]. Additionally, henrischinin B (**213**; the 3D graph is shown in Figure 43) from the leaves and stems of *Schisandra chinensis* displayed activity against HSV-2 virus [179]. A 3D graph representing henrischinin B can be found in Figure 42. Ellarinacin (**214**) is a defense-related arborinane-type triterpenoid that was recently discovered in bread wheat, *Triticum aestivum* [180]. Lovenone (**215**), a cytotoxic degraded triterpenoid, was isolated from skin extracts of the North Sea dorid nudibranch *Adalaria loveni* and exhibited in vitro cytotoxicity against human cancer cell lines [181].

A triterpenoid named applanoid H (**216**) was found in the medicinal fungus *Ganoderma applanatum* and demonstrated PXR (pregnane X receptor) agonistic activity [182]. Two exceptionally uncommon steroids are gymnasterone B (**217**), which was discovered in the marine sponge-associated fungus *Gymnasella dankaliensis* OUPS-N134 isolated from the marine sponge *Halichondria japonica* [183,184,185], and talarosterone (**218**), an ergosterol analog produced by the marine fungus *Talaromyces stipitatus* KUFA 0207 isolated from the marine sponge *Stylissa flabelliformis* (Thailand) [186]. Another steroid, 3,4-epoxy-(22*R*,25)-tetrahydrofuran-stigmast-5-en (**219**), belonging to the stigmastane family, was isolated from the stem bark of *Aglaia eximia*. This compound exhibited cytotoxicity against P-388 murine leukemia cells [187].

## 9. Conclusions

This comprehensive review has explored the diverse range of biological activity and structural variations found within steroids and related isoprenoid lipids. The analysis encompassed various natural compounds, including steroids and isoprenoid lipids featuring α,β-epoxy group(s). These compounds are derived from sources such as fungi, fungal endophytes, plants, algae, and marine invertebrates. Through an examination of refereed literature sources, their biological activity was evaluated through in vivo and in vitro studies, as well as by employing the QSAR method. The findings revealed a multitude of compounds exhibiting remarkable properties, including strong antineoplastic, antiproliferative, anti-eczematic, anti-psoriatic, and various other activities. To enhance comprehension, the review incorporated visual aids such as 3D graphs illustrating the activity of individual steroids and images showcasing selected terrestrial or marine organisms. Furthermore, the review provided explanations elucidating certain types of biological activity associated with these compounds. Overall, the findings presented in this review not only contribute to the academic scientific knowledge in the field but also hold practical relevance for the development of pharmacological interventions and advancements in practical medicine. This review utilized data from various authors regarding the biological activity of natural steroids. To assess the potential activity of these steroids, the PASS program was employed. The PASS program utilizes the structural features of compounds to predict their biological activity profiles.

## Figures and Tables

**Table 1:** Biological activities of steroids bearing 4,5-epoxy group (**1**–**5**).

No.	Dominated Biological Activity (Pa) *	Additional Predicted Activities (Pa) *
1	Apoptosis agonist (0.931) Antineoplastic (0.926) Antineoplastic (liver cancer) (0.743) Prostate cancer treatment (0.626) Antineoplastic (lymphocytic leukemia) (0.614)	Anti-hypercholesterolemic (0.852) Immunosuppressant (0.835) Hepatic disorders treatment (0.822) Anti-eczematic (0.791) Anti-psoriatic (0.777)
2	Antineoplastic (0.873) Apoptosis agonist (0.860) Antimetastatic (0.774) Antineoplastic (lymphocytic leukemia) (0.715)	Respiratory analeptic (0.885) Antifungal (0.812) Immunosuppressant (0.811) Anti-inflammatory (0.737)
3	Antineoplastic (0.855) Angiogenesis inhibitor (0.707) Apoptosis agonist (0.698)	Hepatic disorders treatment (0.851) Antifungal (0.792) Immunosuppressant (0.788)
4	Antineoplastic (0.900) Proliferative diseases treatment (0.784) Apoptosis agonist (0.664)	Respiratory analeptic (0.880) Immunosuppressant (0.866) Antifungal (0.817)
5	Antineoplastic (0.896) Apoptosis agonist (0.886)	Antifungal (0.730) Anti-inflammatory (0.705)

* Only activities with Pa > 0.6 are shown.

**Table 2:** Biological activities of steroids bearing 5,6-epoxy group (**6**–**20**).

No.	Dominated Biological Activity (Pa) *	Additional Predicted Activities (Pa) *
6	Respiratory analeptic (0.911) Hypolipemic (0.779)	Apoptosis agonist (0.867) Antineoplastic (0.851)
7	Apoptosis agonist (0.954) Antineoplastic (0.895)	Anti-osteoporotic (0.789) Anti-psoriatic (0.774)
8	Apoptosis agonist (0.950) Antineoplastic (0.886)	Anti-hypercholesterolemic (0.931) Atherosclerosis treatment (0.712)
9	Apoptosis agonist (0.890) Antineoplastic (0.853)	Anti-hypercholesterolemic (0.872) Atherosclerosis treatment (0.746)
10	Antifungal (0.738)	Anti-inflammatory (0.733)
11	Apoptosis agonist (0.782)	Anti-inflammatory (0.756)
12	Antibacterial (0.704)	Antifungal (0.633)
13	Antibacterial (0.691)	Antifungal (0.577)
14	Apoptosis agonist (0.907) Antineoplastic (0.860)	Anti-eczematic (0.732) Anti-psoriatic (0.659)
15	Apoptosis agonist (0.844) Antineoplastic (0.796)	Anesthetic (0.689) Antipruritic, allergic (0.632)
16	Apoptosis agonist (0.900) Antineoplastic (0.866)	Respiratory analeptic (0.819) Anti-hypercholesterolemic (0.803)
17	Apoptosis agonist (0.866) Antineoplastic (0.863)	Respiratory analeptic (0.835) Hypolipemic (0.820)
18	Apoptosis agonist (0.954) Antineoplastic (0.914)	Anti-hypercholesterolemic (0.906) Atherosclerosis treatment (0.741)
19	Antineoplastic (0.798) Apoptosis agonist (0.751)	Immunosuppressant (0.759) Respiratory analeptic (0.679)
20	Respiratory analeptic (0.953)	Anti-hypercholesterolemic (0.874)

* Only activities with Pa > 0.7 are shown.

**Table 3:** Biological activities of steroids bearing 5,6-epoxy group (**21**–**34**).

No.	Dominated Biological Activity (Pa) *	Additional Predicted Activities (Pa) *
21	Antineoplastic (0.797)	Antibacterial (0.788)
22	Respiratory analeptic (0.934)	Anti-hypercholesterolemic (0.874)
23	Antineoplastic (0.798) Apoptosis agonist (0.751)	Immunosuppressant (0.759) Hypolipemic (0.732)
24	Respiratory analeptic (0.979) Immunosuppressant (0.832)	Antineoplastic (0.872) Apoptosis agonist (0.861)
25	Apoptosis agonist (0.913) Antineoplastic (0.907)	Anti-eczematic (0.844) Anti-psoriatic (0.813)
26	Apoptosis agonist (0.950) Antineoplastic (0.886)	Anti-hypercholesterolemic (0.931) Atherosclerosis treatment (0.712)
27	Respiratory analeptic (0.973) Immunosuppressant (0.795)	Apoptosis agonist (0.896) Antineoplastic (0.880)
28	Respiratory analeptic (0.974) Immunosuppressant (0.788)	Antineoplastic (0.874) Apoptosis agonist (0.868)
29	Anti-hypercholesterolemic (0.934) Hypolipemic (0.864)	Apoptosis agonist (0.929) Antineoplastic (0.861)
30	Respiratory analeptic (0.948) Immunosuppressant (0.828)	Apoptosis agonist (0.941) Antineoplastic (0.924)
31	Antineoplastic (0.791) Apoptosis agonist (0.731)	Immunosuppressant (0.747)
32	Antineoplastic (0.816) Apoptosis agonist (0.732)	Antifungal (0.784) Anti-inflammatory (0.735)
33	Antineoplastic (0.796) Apoptosis agonist (0.729)	Antifungal (0.738) Anti-inflammatory (0.733)
34	Anti-inflammatory (0.817) Antifungal (0.770)	Antineoplastic (0.800) Apoptosis agonist (0.749)

* Only activities with Pa > 0.7 are shown.

**Table 4:** Biological activities of steroids bearing 7,8-epoxy group (**35**–**51**).

No.	Dominated Biological Activity (Pa) *	Additional Predicted Activities (Pa) *
35	Apoptosis agonist (0.906) Antineoplastic (0.879)	Respiratory analeptic (0.796) Immunosuppressant (0.769)
36	Apoptosis agonist (0.945) Antineoplastic (0.918)	Anti-eczematic (0.852) Anti-psoriatic (0.772)
37	Hepatoprotectant (0.994) Anti-hypercholesterolemic (0.897) Immunosuppressant (0.836)	Respiratory analeptic (0.990) Antithrombotic (0.883) Antidiabetic (0.720)
38	Antineoplastic (0.874) Apoptosis agonist (0.753) Prostate disorders treatment (0.549)	Anti-eczematic (0.841) Anti-inflammatory (0.648) Anti-psoriatic (0.629)
39	Respiratory analeptic (0.961) Immunosuppressant (0.780)	Apoptosis agonist (0.856) Antineoplastic (0.812)
40	Respiratory analeptic (0.961) Antiviral (Influenza) (0.667)	Apoptosis agonist (0.856) Antineoplastic (0.812)
41	Respiratory analeptic (0.955) Anti-inflammatory (0.870)	Antineoplastic (0.843) Apoptosis agonist (0.752)
42	Respiratory analeptic (0.937) Anti-inflammatory (0.747) Anesthetic (0.711)	Apoptosis agonist (0.812) Antineoplastic (0.812) Antithrombotic (0.681)
43	Apoptosis agonist (0.872) Antineoplastic (0.848)	Anti-osteoporotic (0.656) Proliferative diseases treatment (0.601)
44	Respiratory analeptic (0.910) Immunosuppressant (0.797) Anti-hypercholesterolemic (0.612)	Apoptosis agonist (0.859) Antineoplastic (0.847) Proliferative diseases treatment (0.650)
45	Respiratory analeptic (0.897) Immunosuppressant (0.792) Cardiotonic (0.626)	Apoptosis agonist (0.859) Antineoplastic (0.806) Proliferative diseases treatment (0.633)
46	Anti-inflammatory (0.880) Respiratory analeptic (0.849) Septic shock treatment (0.681)	Antineoplastic (0.842) Anti-hypercholesterolemic (0.739) Apoptosis agonist (0.712)
47	Respiratory analeptic (0.978) Immunosuppressant (0.792) Proliferative diseases treatment (0.679)	Anti-eczematic (0.952) Antineoplastic (0.912) Apoptosis agonist (0.911)
48	Apoptosis agonist (0.901) Proliferative diseases treatment (0.702)	Anti-eczematic (0.870) Anti-psoriatic (0.753)
49	Apoptosis agonist (0.894) Atherosclerosis treatment (0.745)	Anti-eczematic (0.825) Anti-psoriatic (0.738)
50	Antineoplastic (0.867) Apoptosis agonist (0.754)	Anti-eczematic (0.797) Anti-psoriatic (0.706)
51	Chemopreventive (0.900) Antineoplastic (0.848) Apoptosis agonist (0.811)	Anti-hypercholesterolemic (0.852) Respiratory analeptic (0.827) Hypolipemic (0.760)

* Only activities with Pa > 0.7 are shown.

**Table 5:** Biological activities of steroids bearing 8,14-, 9,11-, and 11,12-epoxy groups (**52**–**72**).

No.	Dominated Biological Activity (Pa) *	Additional Predicted Activities (Pa) *
52	Respiratory analeptic (0.961) Immunosuppressant (0.800)	Apoptosis agonist (0.914) Proliferative diseases treatment (0.693)
53	Respiratory analeptic (0.950) Immunosuppressant (0.795)	Antineoplastic (0.910) Apoptosis agonist (0.908)
54	Apoptosis agonist (0.920) Proliferative diseases treatment (0.708)	Respiratory analeptic (0.843) Hypolipemic (0.679)
55	Antineoplastic (0.908) Apoptosis agonist (0.906) Proliferative diseases treatment (0.656)	Respiratory analeptic (0.872) Anti-hypercholesterolemic (0.786) Cholesterol synthesis inhibitor (0.647)
56	Respiratory analeptic (0.909) Immunosuppressant (0.779)	Antineoplastic (0.882) Apoptosis agonist (0.843)
57	Respiratory analeptic (0.961) Immunosuppressant (0.800)	Antineoplastic (0.916) Apoptosis agonist (0.914)
58	Respiratory analeptic (0.909) Immunosuppressant (0.779)	Antineoplastic (0.882) Apoptosis agonist (0.843)
59	Respiratory analeptic (0.922) Immunosuppressant (0.799)	Antineoplastic (0.908) Apoptosis agonist (0.874)
60	Antineoplastic (0.908) Apoptosis agonist (0.896)	Respiratory analeptic (0.881) Immunosuppressant (0.788)
61	Antineoplastic (0.890) Apoptosis agonist (0.683)	Anti-hypercholesterolemic (0.791) Cholesterol synthesis inhibitor (0.712)
62	Antineoplastic (0.890) Apoptosis agonist (0.743)	Anti-hypercholesterolemic (0.793) Atherosclerosis treatment (0.738)
63	Antineoplastic (0.927) Apoptosis agonist (0.692)	Respiratory analeptic (0.587) Cardiovascular analeptic (0.556)
64	Inhibitor rheumatoid arthritis (0.928) Inhibitor pregnane x receptor (0.729)	Alzheimer’s disease treatment (0.676) Anti-nephrotoxic (0.637)
65	Chemopreventive (0.890) Antineoplastic (0.850) Apoptosis agonist (0.781)	Anti-hypercholesterolemic (0.816) Atherosclerosis treatment (0.722) Biliary tract disorders treatment (0.667)
66	Antifungal (0.887) Antibacterial (0.804)	Antileukemic (0.756) Prostate disorders treatment (0.735)
67	Antifungal (0.845) Antibacterial (0.794)	Antileukemic (0.730) Prostate disorders treatment (0.717)
68	Antifungal (0.845) Antibacterial (0.794)	Antileukemic (0.730) Prostate disorders treatment (0.717)
69	Antiprotozoal (0.944) Genital warts treatment (0.811)	Antineoplastic (0.766) Antimetastatic (0.642)
70	Antifungal (0.911) Antibacterial (0.822)	Antileukemic (0.714) Prostate disorders treatment (0.696)
71	Antiviral (0.912) Antiviral (Influenza) (0.901)	Respiratory analeptic (0.855) Immunosuppressant (0.721)
72	Anti-inflammatory (0.893) Antiviral (Influenza) (0.787)	Antifungal (0.865) Antibacterial (0.814)

* Only activities with Pa > 0.7 are shown.

**Table 6:** Biological activities of steroids bearing 17,20-epoxy group (**73**–**87**).

No.	Dominated Biological Activity (Pa) *	Additional Predicted Activities (Pa) *
73	Anesthetic general (0.928) Angiogenesis inhibitor (0.918) Respiratory analeptic (0.916)	Antineoplastic (0.914) Anti-hypercholesterolemic (0.857) Immunosuppressant (0.762)
74	Angiogenesis inhibitor (0.910) Anesthetic general (0.774)	Respiratory analeptic (0.848) Anti-hypercholesterolemic (0.841)
75	Anesthetic general (0.928) Angiogenesis inhibitor (0.892)	Respiratory analeptic (0.916) Antineoplastic (0.903)
76	Angiogenesis inhibitor (0.890) Analeptic (0.872)	Anti-hypercholesterolemic (0.813)
77	Angiogenesis inhibitor (0.916) Analeptic (0.834)	Anti-hypercholesterolemic (0.785) Anti-inflammatory (0.705)
78	Angiogenesis inhibitor (0.825) Anesthetic general (0.666)	Anti-osteoporotic (0.820) Antiallergic (0.710)
79	Angiogenesis inhibitor (0.869) Immunosuppressant (0.716)	Antineoplastic (0.858) Apoptosis agonist (0.651)
80	Angiogenesis inhibitor (0.920) Anesthetic general (0.783)	Respiratory analeptic (0.855) Anti-hypercholesterolemic (0.823)
81	Angiogenesis inhibitor (0.877) Immunosuppressant (0.744)	Antineoplastic (0.889) Apoptosis agonist (0.687)
82	Angiogenesis inhibitor (0.891) Immunosuppressant (0.733)	Antineoplastic (0.836) Apoptosis agonist (0.677)
83	Angiogenesis inhibitor (0.897) Analeptic (0.837)	Anti-hypercholesterolemic (0.765) Anti-inflammatory (0.765)
84	Angiogenesis inhibitor (0.902) Analeptic (0.866)	Anti-hypercholesterolemic (0.777) Anti-inflammatory (0.754)
85	Anesthetic general (0.931) Angiogenesis inhibitor (0.922) Respiratory analeptic (0.921)	Antineoplastic (0.923) Anti-hypercholesterolemic (0.862) Immunosuppressant (0.774)
86	Angiogenesis inhibitor (0.878) Immunosuppressant (0.736)	Antineoplastic (0.828) Apoptosis agonist (0.633)
87	Antiprotozoal (0.944) Antiprotozoal (Plasmodium) (0.937)	Genital warts treatment (0.767) Antineoplastic (0.722)

* Only activities with Pa > 0.7 are shown.

**Table 7:** Biological activities of steroids bearing 22,23-epoxy group (**88**–**108**).

No.	Dominated Biological Activity (Pa) *	Additional Predicted Activities (Pa) *
88	Anti-hypercholesterolemic (0.901) Lipid metabolism regulator (0.833) Antiallergic (0.723)	Respiratory analeptic (0.889) Ovulation inhibitor (0.884) Anesthetic general (0.843)
89	Anti-hypercholesterolemic (0.924) Lipid metabolism regulator (0.820)	Respiratory analeptic (0.927) Ovulation inhibitor (0.858)
90	Angiogenesis inhibitor (0.943) Antidiabetic symptomatic (0.835) Autoimmune disorders treatment (0.794)	Antineoplastic (0.924) Respiratory analeptic (0.877) Lipid metabolism regulator (0.790)
91	Hepatic disorders treatment (0.917) Immunosuppressant (0.774)	Antineoplastic (0.836) Angiogenesis inhibitor (0.828)
92	Hepatic disorders treatment (0.878) Cholesterol synthesis inhibitor (0.704)	Antineoplastic (0.844) Angiogenesis inhibitor (0.788)
93	Hepatic disorders treatment (0.903) Cholesterol synthesis inhibitor (0.641)	Antineoplastic (0.824) Angiogenesis inhibitor (0.765)
94	Hepatic disorders treatment (0.878) Cholesterol synthesis inhibitor (0.704)	Antineoplastic (0.844) Angiogenesis inhibitor (0.788)
95	Hepatic disorders treatment (0.883) Cholesterol synthesis inhibitor (0.737)	Antineoplastic (0.841) Angiogenesis inhibitor (0.809)
96	Hepatic disorders treatment (0.908) Cholesterol synthesis inhibitor (0.683)	Antineoplastic (0.820) Angiogenesis inhibitor (0.793)
97	Hepatic disorders treatment (0.883) Cholesterol synthesis inhibitor (0.737)	Antineoplastic (0.841) Angiogenesis inhibitor (0.809)
98	Hepatic disorders treatment (0.883) Cholesterol synthesis inhibitor (0.737)	Antineoplastic (0.841) Angiogenesis inhibitor (0.809)
99	Hepatic disorders treatment (0.908) Cholesterol synthesis inhibitor (0.683)	Angiogenesis inhibitor (0.793) Biliary tract disorders treatment (0.621)
100	Hepatic disorders treatment (0.883) Cholesterol synthesis inhibitor (0.737)	Angiogenesis inhibitor (0.809) Biliary tract disorders treatment (0.611)
101	Hepatic disorders treatment (0.919) Immunosuppressant (0.768)	Antineoplastic (0.811) Angiogenesis inhibitor (0.782)
102	Hepatic disorders treatment (0.905) Immunosuppressant (0.772)	Antineoplastic (0.800) Angiogenesis inhibitor (0.709)
103	Hepatic disorders treatment (0.905) Immunosuppressant (0.772)	Antineoplastic (0.800) Angiogenesis inhibitor (0.709)
104	Hepatic disorders treatment (0.880) Immunosuppressant (0.786)	Antifungal (0.829) Antibacterial (0.737)
105	Hepatic disorders treatment (0.880) Immunosuppressant (0.786)	Antifungal (0.829) Antibacterial (0.737)
106	Hepatic disorders treatment (0.880) Immunosuppressant (0.786)	Antifungal (0.829) Antibacterial (0.737)
107	Hepatic disorders treatment (0.910) Immunosuppressant (0.758)	Antifungal (0.803) Antineoplastic (0.797)
108	Hepatic disorders treatment (0.910) Immunosuppressant (0.758)	Antifungal (0.803) Antineoplastic (0.797)

* Only activities with Pa > 0.7 are shown.

**Table 8:** Biological activities of steroids bearing 22,23-epoxy group (**109**–**120**).

No.	Dominated Biological Activity (Pa) *	Additional Predicted Activities (Pa) *
109	Hepatic disorders treatment (0.890) Immunosuppressant (0.785)	Antineoplastic (0.813) Angiogenesis inhibitor (0.751)
110	Hepatic disorders treatment (0.886) Immunosuppressant (0.795)	Antineoplastic (0.816) Angiogenesis inhibitor (0.728)
111	Hepatic disorders treatment (0.886) Immunosuppressant (0.795)	Antineoplastic (0.816) Angiogenesis inhibitor (0.728)
112	Hepatic disorders treatment (0.886) Immunosuppressant (0.795)	Antineoplastic (0.816) Angiogenesis inhibitor (0.728)
113	Hepatic disorders treatment (0.886) Immunosuppressant (0.795)	Antineoplastic (0.816) Angiogenesis inhibitor (0.728)
114	Antifungal (0.860) Antibacterial (0.803)	Acute neurologic disorders treatment (0.819) Biliary tract disorders treatment (0.783)
115	Antifungal (0.890) Antibacterial (0.875)	Antineoplastic (0.857) Acute neurologic disorders treatment (0.681)
116	Antifungal (0.902) Antibacterial (0.886)	Antineoplastic (0.858) Acute neurologic disorders treatment (0.727)
117	Antifungal (0.863) Antibacterial (0.803)	Acute neurologic disorders treatment (0.822) Biliary tract disorders treatment (0.783)
118	Antibacterial (0.906) Antifungal (0.902)	Antineoplastic (0.849) Acute neurologic disorders treatment (0.667)
119	Antifungal (0.885) Antibacterial (0.873)	Antineoplastic (0.849) Acute neurologic disorders treatment (0.692)
120	Antifungal (0.893) Antibacterial (0.895)	Antineoplastic (0.863) Acute neurologic disorders treatment (0.712)

* Only activities with Pa > 0.7 are shown.

**Table 9:** Biological activities of miscellaneous steroids and isoprenoid lipids (**121**–**139**).

No.	Dominated Biological Activity (Pa) *	Additional Predicted Activities (Pa) *
121	Anti-hypercholesterolemic (0.886) Antineoplastic (0.846) Anesthetic general (0.690)	Antifungal (0.727) Angiogenesis inhibitor (0.680) Antibacterial (0.666)
122	Antineoplastic (0.938) Apoptosis agonist (0.923) Antileukemic (0.785)	Anti-eczematic (0.921) Anti-psoriatic (0.771) Hypolipemic (0.669)
123	Antineoplastic (0.918) Apoptosis agonist (0.908)	Anti-eczematic (0.889) Anti-psoriatic (0.732)
124	Anti-hypercholesterolemic (0.891) Antineoplastic (0.833)	Antifungal (0.739) Antibacterial (0.658)
125	Antineoplastic (0.918) Apoptosis agonist (0.793) Prostate cancer treatment (0.679)	Cachexia treatment (0.749) Anti-osteoporotic (0.672) Menopausal disorders treatment (0.582)
126	Antineoplastic (0.919) Apoptosis agonist (0.798) Prostate cancer treatment (0.688)	Cachexia treatment (0.773) Anti-osteoporotic (0.689) Menopausal disorders treatment (0.613)
127	Apoptosis agonist (0.954) Antineoplastic (0.888)	Genital warts treatment (0.914) Anesthetic general (0.730)
128	Apoptosis agonist (0.933) Antineoplastic (0.867)	Genital warts treatment (0.897) Anesthetic general (0.713)
129	Apoptosis agonist (0.924) Antineoplastic (0.839)	Genital warts treatment (0.902) Anesthetic general (0.722)
130	Apoptosis agonist (0.917) Antineoplastic (0.831)	Genital warts treatment (0.899) Anesthetic general (0.741)
131	Chemopreventive (0.892) Apoptosis agonist (0.854) Antineoplastic (0.790)	Hypolipemic (0.852) Anti-hypercholesterolemic (0.718) Cholesterol synthesis inhibitor (0.543)
132	Apoptosis agonist (0.844) Antineoplastic (0.771)	Antifungal (0.813) Antibacterial (0.712)
133	Antineoplastic (0.767) Apoptosis agonist (0.722)	Antifungal (0.723) Antibacterial (0.698)
134	Chemopreventive (0.934) Apoptosis agonist (0.914) Antineoplastic (0.881)	Hypolipemic (0.902) Anti-hypercholesterolemic (0.887) Cholesterol synthesis inhibitor (0.841)
135	Chemopreventive (0.928) Apoptosis agonist (0.922) Antineoplastic (0.916)	Hypolipemic (0.911) Anti-hypercholesterolemic (0.866) Cholesterol synthesis inhibitor (0.812)
136	Chemopreventive (0.933) Antineoplastic (0.923)	Anti-hypercholesterolemic (0.899) Cholesterol synthesis inhibitor (0.878)
137	Antineoplastic (0.865) Apoptosis agonist (0.834)	Anti-hypercholesterolemic (0.812) Cholesterol synthesis inhibitor (0.722)
138	Antineoplastic (0.947) Chemopreventive (0.922)	Anti-hypercholesterolemic (0.911) Cholesterol synthesis inhibitor (0.892)
139	Antineoplastic (0.887) Apoptosis agonist (0.851)	Anti-hypercholesterolemic (0.843) Cholesterol synthesis inhibitor (0.752)

* Only activities with Pa > 0.7 are shown.

**Table 10:** Biological activities of miscellaneous steroids and isoprenoid lipids (**140**–**159**).

No.	Dominated Biological Activity (Pa) *	Additional Predicted Activities (Pa) *
140	Chemopreventive (0.893) Apoptosis agonist (0.841)	Antifungal (0.815) Anti-inflammatory (0.793)
141	Nitric oxide production inhibitor (0.922) Anti-hypercholesterolemic (0.818)	Cholesterol synthesis inhibitor (0.743) Antibacterial (0.652)
142	Antineoplastic (0.980) Chemopreventive (0.942) Apoptosis agonist (0.913)	Antibacterial (0.882) Antifungal (0.819) Anti-inflammatory (0.803)
143	Antineoplastic (0.902) Apoptosis agonist (0.862)	Anti-hypercholesterolemic (0.733) Cholesterol synthesis inhibitor (0.644)
144	Anti-inflammatory (0.903) Antibacterial (0.876) Antifungal (0.833)	Anti-hypercholesterolemic (0.784) Cholesterol synthesis inhibitor (0.721) Hypolipemic (0.652)
145	Anesthetic general (0.937) Angiogenesis inhibitor (0.922)	Antineoplastic (0.910) Anti-hypercholesterolemic (0.844)
146	Antineoplastic (0.912) Cytostatic (0.878) Apoptosis agonist (0.845)	Antibacterial (0.854) Antifungal (0.799) Anti-inflammatory (0.769)
147	Antineoplastic (0.944) Apoptosis agonist (0.921)	Anti-hypercholesterolemic (0.815) Cholesterol synthesis inhibitor (0.766)
148	Anesthetic general (0.883) Angiogenesis inhibitor (0.821)	Antineoplastic (0.856) Anti-hypercholesterolemic (0.811)
149	Anesthetic general (0.897) Angiogenesis inhibitor (0.832)	Antineoplastic (0.871) Anti-hypercholesterolemic (0.835)
150	Anesthetic general (0.959) Angiogenesis inhibitor (0.932)	Antineoplastic (0.911) Anti-hypercholesterolemic (0.832)
151	Antineoplastic (0.989) Apoptosis agonist (0.931)	Anti-hypercholesterolemic (0.792) Cholesterol synthesis inhibitor (0.758)
152	Cytostatic (0.922) Apoptosis agonist (0.911)	Antifungal (0.799) Antibacterial (0.854)
153	Cytostatic (0.911) Apoptosis agonist (0.903)	Antifungal (0.832) Antibacterial (0.807)
154	Cytostatic (0.916) Apoptosis agonist (0.892)	Antifungal (0.856) Antibacterial (0.832)
155	Cytostatic (0.938) Apoptosis agonist (0.923)	Antifungal (0.878) Antibacterial (0.859)
156	Anesthetic general (0.907) Angiogenesis inhibitor (0.883)	Antineoplastic (0.882) Anti-hypercholesterolemic (0.821)
157	Anesthetic general (0.916) Angiogenesis inhibitor (0.891)	Antineoplastic (0.899) Anti-hypercholesterolemic (0.837)
158	Anesthetic general (0.956) Angiogenesis inhibitor (0.932)	Antineoplastic (0.909) Anti-hypercholesterolemic (0.788)
159	Anesthetic general (0.956) Angiogenesis inhibitor (0.932)	Antineoplastic (0.909) Anti-hypercholesterolemic (0.788)

* Only activities with Pa > 0.7 are shown.

**Table 11:** Biological activities of miscellaneous steroids and isoprenoid lipids (**160**–**176**).

No.	Dominated Biological Activity (Pa) *	Additional Predicted Activities (Pa) *
160	Carboxylesterase inhibitor (0.922) Anti-hypercholesterolemic (0.822) Cholesterol synthesis inhibitor (0.741)	Antifungal (0.831) Antibacterial (0.820) Anti-inflammatory (0.734)
161	Carboxylesterase inhibitor (0.829) Anti-hypercholesterolemic (0.812) Cholesterol synthesis inhibitor (0.806)	Antifungal (0.745) Antibacterial (0.743) Anti-inflammatory (0.712)
162	Carboxylesterase inhibitor (0.900) Anti-hypercholesterolemic (0.821) Cholesterol synthesis inhibitor (0.808)	Antifungal (0.811) Antibacterial (0.806) Anti-inflammatory (0.698)
163	Carboxylesterase inhibitor (0.943) Anti-hypercholesterolemic (0.928) Cholesterol synthesis inhibitor (0.843)	Antifungal (0.853) Antibacterial (0.824) Anti-inflammatory (0.792)
164	Carboxylesterase inhibitor (0.949) Anti-hypercholesterolemic (0.929) Cholesterol synthesis inhibitor (0.867)	Antifungal (0.858) Antibacterial (0.829) Anti-inflammatory (0.803)
165	Carboxylesterase inhibitor (0.941) Cholesterol synthesis inhibitor (0.855)	Antifungal (0.858) Antibacterial (0.829)
166	Antineoplastic (0.955) Cytostatic (0.927) Apoptosis agonist (0.918)	Antileukemic (0.910) Antimetastatic (0.901) Antineoplastic (0.899)
167	Antineoplastic (0.897) Apoptosis agonist (0.818)	Antimetastatic (0.821) Antineoplastic (0.802)
168	Antineoplastic (0.948) Apoptosis agonist (0.914)	Respiratory analeptic (0.895) Anti-inflammatory (0.834)
169	Apoptosis agonist (0.939) Antineoplastic (0.922)	Antimetastatic (0.914) Antileukemic (0.898)
170	Apoptosis agonist (0.926) Antineoplastic (0.911)	Antimetastatic (0.900) Antileukemic (0.876)
171	Anti-inflammatory (0.923) Antiviral (0.856)	Antineoplastic (0.845) Apoptosis agonist (0.807)
172	Anti-inflammatory (0.911) Antiviral (0.872)	Antineoplastic (0.866) Apoptosis agonist (0.822)
173	Anti-inflammatory (0.931) Antiviral (0.902)	Antineoplastic (0.852) Apoptosis agonist (0.812)
174	Antineoplastic (0.956) Apoptosis agonist (0.804)	Angiogenesis inhibitor (0.893) Lipid metabolism regulator (0.680)
175	Apoptosis agonist (0.889) Antineoplastic (0.875)	Anti-eczematic (0.717) Anti-psoriatic (0.668)
176	Anti-hypercholesterolemic (0.952) Carboxylesterase inhibitor (0.931)	Lipid metabolism regulator (0.880) Cholesterol synthesis inhibitor (0.823)

* Only activities with Pa > 0.7 are shown.

**Table 12:** Biological activities of miscellaneous steroids and isoprenoid lipids (**177**–**204**).

No.	Dominated Biological Activity (Pa) *	Additional Predicted Activities (Pa) *
177	Antineoplastic (0.933) Apoptosis agonist (0.841)	Antifungal (0.802) Antibacterial (0.754)
178	Antineoplastic (0.924) Apoptosis agonist (0.855)	Antifungal (0.786) Antibacterial (0.733)
179	Antineoplastic (0.924)	Antiviral (arbovirus) (0.772)
180	A nitric oxide production inhibitor (0.944) Acetylcholinesterase inhibitor (0.933)	Antibacterial (0.743) Antifungal (0.697)
181	Antifungal (0.921) Antibacterial (0.718)	Antiparasitic (0.728) Antiviral (0.712)
182	Antifungal (0.917) Antibacterial (0.722)	Antiparasitic (0.728) Antiviral (0.744)
183	Cytotoxic (0.896)	Antineoplastic (0.824)
184	Acetylcholinesterase inhibitor (0.908) A nitric oxide production inhibitor (0.858)	Antineoplastic (0.715) Antiviral (0.753)
185	Acetylcholinesterase inhibitor (0.914) Cytotoxic (0.896)	Antineoplastic (0.821) Antiviral (0.744)
186	Cytotoxic (0.922) Acetylcholinesterase inhibitor (0.914) A nitric oxide production inhibitor (0.881)	Antineoplastic (0.832) Apoptosis agonist (0.811) Antifungal (0.657)
187	Antineoplastic (0.876) Apoptosis agonist (0.812)	Antifungal (0.726) Antibacterial (0.711)
188	Antineoplastic (0.882) Apoptosis agonist (0.833)	Antifungal (0.726) Antibacterial (0.702)
189	Antineoplastic (0.811) Apoptosis agonist (0.718)	Antifungal (0.704) Antibacterial (0.674)
190	Cytotoxic (0.857) Apoptosis agonist (0.778)	Antimutagenic (0.710) Anti-asthmatic (0.587)
191	Cytotoxic (0.865) Apoptosis agonist (0.719)	Antimutagenic (0.722) Antiviral (0.711)
192	Cytotoxic (0.881) Apoptosis agonist (0.734)	Antimutagenic (0.721) Antiviral (0.689)
193	Cytotoxic (0.903)	Antineoplastic (0.823)
194	Angiogenesis stimulant (0.872) Apoptosis agonist (0.713)	Lipid metabolism regulator (0.728) Anti-hypercholesterolemic (0.701)
195	Angiogenesis stimulant (0.872) Apoptosis agonist (0.713)	Lipid metabolism regulator (0.728) Anti-hypercholesterolemic (0.701)
196	Angiogenesis stimulant (0.887) Apoptosis agonist (0.767)	Lipid metabolism regulator (0.713) Anti-hypercholesterolemic (0.698)
197	Angiogenesis stimulant (0.902) Apoptosis agonist (0.775)	Lipid metabolism regulator (0.802) Anti-hypercholesterolemic (0.678)
198	Antineoplastic (0.884) Prostate disorders treatment (0.649)	Anti-eczematic (0.852) Anti-psoriatic (0.678)
199	Antineoplastic (0.855) Prostate disorders treatment (0.688)	Anti-eczematic (0.712) Anti-psoriatic (0.614)
200	Apoptosis agonist (0.881) Proliferative diseases treatment (0.711)	Respiratory analeptic (0.817) Hypolipemic (0.655)
201	Apoptosis agonist (0.880) Proliferative diseases treatment (0.721)	Respiratory analeptic (0.821) Hypolipemic (0.638)
202	Antineoplastic (0.877) Apoptosis agonist (0.766)	Lipid metabolism regulator (0.781) Anti-hypercholesterolemic (0.652)
203	Antineoplastic (0.873)	Proliferative diseases treatment (0.814)
204	Antineoplastic (0.793)	Proliferative diseases treatment (0.785)

* Only activities with Pa > 0.7 are shown.

**Table 13:** Biological activities of miscellaneous steroids and isoprenoid lipids (**205**–**219**).

No.	Dominated Biological Activity (Pa) *	Additional Predicted Activities (Pa) *
205	Antiviral (HIV) 0.876 Antiviral (arbovirus) (0.712)	Antifungal (0.742) Antibacterial (0.656)
206	Antiviral (HIV) 0.938 Antiviral (influenza A) (0.894) Antiviral (arbovirus) (0.783)	Antifungal (0.722) Antibacterial (0.632) Antiparasitic (0.618)
207	Antiviral (HIV) 0.951 Antiviral (influenza A) (0.849) Antiviral (arbovirus) (0.754)	Antifungal (0.768) Antibacterial (0.692) Antiparasitic (0.610)
208	Antiviral (HIV) 0.845 Antiviral (arbovirus) (0.699)	Antifungal (0.731) Antibacterial (0.655)
209	Antiviral (HIV) 0.866	Antibacterial (0.699)
210	Antiviral (arbovirus) (0.823) Antiviral (0.769)	Antifungal (0.788) Antibacterial (0.642)
211	Antiviral (HSV-1) (0.971) Antiviral (HIV) (0.958) Antiviral (influenza A) (0.878)	Antifungal (0.792) Antibacterial (0.654) Antiparasitic (0.647)
212	Antiviral (HSV-1) (0.936) Antiviral (HIV) (0.922)	Antifungal (0.722) Antibacterial (0.633)
213	Antiviral (HSV-2) (0.984) Antiviral (HIV) (0.939)	Antifungal (0.792) Antibacterial (0.677)
214	Cytotoxic (0.912) Antineoplastic (0.886)	Lipid metabolism regulator (0.823) Anti-hypercholesterolemic (0.732)
215	Cytotoxic (0.932) Antineoplastic (0.893)	Lipid metabolism regulator (0.842) Anti-hypercholesterolemic (0.752)
216	PXR agonistic (0.933) Antiviral (arbovirus) (0.719)	Antineoplastic (0.811) Apoptosis agonist (0.729)
217	Antineoplastic (0.879) Apoptosis agonist (0.743)	Antimutagenic (0.755) Antileukemic (0.726)
218	Antineoplastic (0.863) Apoptosis agonist (0.712)	Antimutagenic (0.764) Antileukemic (0.721)
219	Antineoplastic (0.922) Apoptosis agonist (0.705)	Antileukemic (0.766) Antimutagenic (0.733)

* Only activities with Pa > 0.7 are shown.

## Data Availability

Not applicable.

## References

[1] Walsh A.D. (1949). The structures of ethylene oxide, cyclopropane, and related molecules. Trans. Faraday Soc..

[2] Fahy E., Cotter D., Sud M., Subramaniam S. (2011). Lipid classification, structures, and tools. Biochim. Biophys. Acta.

[3] Meng Y., Taddeo F., Aguilera A.F., Cai X., Russo V., Tolvanen P., Leveneur S. (2021). The lord of the chemical rings: Catalytic synthesis of important industrial epoxide compounds. Catalysts.

[4] Huisgen R. (1977). Electrocyclic ring opening reactions of ethylene oxides. Angew. Chem. Int. Ed..

[5] Moser B.R., Cermak S.C., Doll K.M., Kenar J.A., Sharma B.K. (2022). A review of fatty epoxide ring-opening reactions: Chemistry, recent advances, and applications. J. Am. Oil Chem. Soc..

[6] Meninno S., Lattanzi A. (2016). Organocatalytic asymmetric reactions of epoxides: Recent progress. Chem. Eur. J..

[7] Bhosale S.V., Bhosale S.V. (2007). β-Cyclodextrin as a catalyst in organic synthesis. Mini-Rev. Org. Chem..

[8] Singh G.S., Mollet K., D’hooghe M., De Kimpe N. (2013). Epihalohydrins in organic synthesis. Chem. Rev..

[9] Moss G.P. (1989). Nomenclature of steroids. Pure Appl. Chem..

[10] Russel C.A., Russell C.A., Roberts G.K. (2005). Organic chemistry: Natural products, steroids. Chemical History: Reviews of the Recent Literature.

[11] Dembitsky V.M., Kuklev D.V., Ahmad M.U. (2017). Acetylenic epoxy fatty acids: Chemistry, synthesis, and their pharmaceutical applications. Fatty Acids.

[12] Vil V., Gloriozova T.A., Poroikov V.V., Savidov N., Dembitsky V.M. (2019). Naturally occurring of α, β-diepoxy-containing compounds: Origin, structures, and biological activities. Appl. Microbiol. Biotech..

[13] Vil V., Al Quntar A.A.A., Gloriozova T.A., Savidov N., Dembitsky V.M. (2019). Oxetane-containing metabolites: Origin, structures, and biological activities. Appl. Microbiol. Biotechnol..

[14] Kuklev D.V., Dembitsky V.M. (2014). Epoxy acetylenic lipids: Their analogues and derivatives. Prog. Lipid Res..

[15] Dembitsky V.M., Gloriozova T.A., Poroikov V.V. (2018). Naturally occurring marine α,β-epoxy steroids: Origin and biological activities. Vietnam J. Chem..

[16] Saikia S., Kolita B., Dutta P.P., Dutta D.J., Neipihoi S. (2015). Marine steroids as potential anticancer drug candidates: In silico investigation in search of inhibitors of Bcl-2 and CDK-4/Cyclin D1. Steroids.

[17] Zhang H., Zhao Z., Wang H. (2017). Cytotoxic natural products from marine sponge-derived microorganisms. Mar. Drugs.

[18] Mioso R., Marante F.J.T., de Souza Bezerra R., Pereira Borges F.V., de Oliveira Santos B.V. (2017). Cytotoxic compounds derived from marine sponges, A review (2010–2012). Molecules.

[19] Dembitsky V.M., Rezanka T., Srebnik M. (2003). Lipid compounds of freshwater sponges: Family Spongillidae, class Demospongiae. Chem. Phys. Lipids.

[20] Dembitsky V.M. (2006). Anticancer activity of natural and synthetic acetylenic lipids. Lipids.

[21] Garridoa L., Zubíaa E., Ortegaa M.J., Salvá J. (2000). Isolation and structure elucidation of new cytotoxic steroids from the gorgonian *Leptogorgia sarmentosa*. Steroids.

[22] Kicha A.A., Ivanchina N.V., Kalinovsky A.I., Dmitrenok P.S., Stonik V.A. (2000). Steroidal monoglycosides from the Far Eastern starfish *Hippasteria kurilensis* and hypothetic pathways of polyhydroxysteroid biosynthesis in starfish. Steroids.

[23] Lerch M.L., Faulkner D.J. (2001). Unusual polyoxygenated sterols from a Philippines sponge *Xestospongia* sp.. Tetrahedron.

[24] Aiello A., Fattorusso E., Menna M. (1999). Steroids from sponges: Recent reports. Steroids.

[25] D’Auria M.V., Minale L., Riccio R. (1993). Polyoxygenated steroids of marine origin. Chem. Rev..

[26] Gottfried H. (1964). The occurrence and biological significance of steroids in lower vertebrates. A review. Steroids.

[27] Xu S., Liao X., Du B., Zhou X., Huang Q., Wu C. (2008). A series of new 5,6-epoxysterols from a Chinese sponge *Ircinia aruensis*. Steroids.

[28] Shen Y.C., Prakash C.V.S., Chang Y.T. (2001). Two new polyhydroxysteroids from the gorgonian *Isis hippuris*. Steroids.

[29] Tanaka J., Trianto A., Musman M., Issa H.H., Ohtani I.I., Ichiba T., Higa T., Yoshida W.Y., Scheuer P.J. (2002). New polyoxygenated steroids exhibiting reversal of multidrug resistance from the gorgonian Isis hippuris. Tetrahedron.

[30] Naz S., Kerr R.G., Narayanan R. (2000). New antiproliferative epoxysecosterols from *Pseudopterogorgia americana*. Tetahedron Lett..

[31] Morris L.A., Christie E.M., Jaspars M., van Ofwegen L.P. (1998). A bioactive secosterol with an unusual A- and B-ring oxygenation pattern isolated from an Indonesian soft coral *Lobophytum* sp.. J. Nat. Prod..

[32] Pika J., Tischler M., Andersen R.J. (2011). Glaciasterols A and B, 9,11-secosteroids from the marine sponge *Aplysilla glacialis*. Can. J. Chem..

[33] Luo X., Li F., Shinde P.B., Hong J., Lee C.-O., Im K.S., Jung J.H. (2006). 26,27-Cyclosterols and other polyoxygenated sterols from a marine sponge *Topsentia* sp.. J. Nat. Prod..

[34] Su J.-H., Tseng Y.-J., Huang H.-H., Ahmed A.F., Lu C.-K. (2006). 9,11-Secosterols from the soft corals *Sinularia lochmodes* and *Sinularia leptoclados*. J. Nat. Prod..

[35] Ahmed A.F., Hsieh Y.-T., Wen Z.-H., Wu Y.-C., Sheu J.-H. (2006). Polyoxygenated sterols from the Formosan soft coral *Sinularia gibberosa*. J. Nat. Prod..

[36] Duh C.-Y., Lo I.-W., Wang S.-K., Dai C.-F. (2007). New cytotoxic steroids from the soft coral *Clavularia viridis*. Steroids.

[37] Ahmed A.F., Tai S.-H., Wu Y.-C., Sheu J.-H. (2007). Sinugrandisterols A–D, trihydroxysteroids from the soft coral *Sinularia grandilobata*. Steroids.

[38] Dembitsky V.M. (2021). In silico prediction of steroids and triterpenoids as potential regulators of lipid metabolism. Mar. Drugs.

[39] Tung N.H., Minh C.V., Ha T.T., Kiem P.V., Huong H.T., Dat N.T., Nhiem N.X. (2009). C29 sterols with a cyclopropane ring at C-25 and 26 from the Vietnamese marine sponge *Ianthella* sp. and their anticancer properties. Bioorganic Med. Chem. Lett..

[40] Shaaban M., Ghani M.A., Shaaban K.A. (2013). Zahramycins A-B, Two new steroids from the Coral *Sarcophyton trocheliophorum*. Z. Naturforsch..

[41] Zhang H.J., Yi Y.H., Yang F., Chen W.S., Lin H.W. (2010). Sesterterpenes and a new sterol from the marine sponge *Phyllospongia foliascens*. Molecules.

[42] Watanabe K., Iwashim M., Iguchi K. (1996). New bioactive marine steroids from the Okinawan soft coral *Clavularia viridis*. Steroids.

[43] Uddin M.H., Hanif N., Trianto A., Agarie Y., Higa T., Tanaka J. (2011). Four new polyoxygenated gorgosterols from the gorgonian *Isis hippuris*. Nat. Prod. Res..

[44] Chen W.-H., Wang S.-K., Duh C.-Y. (2011). Polyhydroxylated steroids from the octocoral *Isis hippuris*. Tetrahedron.

[45] Rodewald W.J., Bończa-Tomaszewski Z. (1979). Intramolecular cyclization of 3β-acetoxy-5-oxo-7-formyl-7α,8-epoxy-5,6-secocholestane into ketal-acetals. Tetrahedron Lett..

[46] Afiyatullov S.S., Kalinovsky A.I., Antonov A.S., Ponomarenko L.P. (2007). Isolation and structures of erylosides from the Carribean sponge *Erylus goffrilleri*. J. Nat. Prod..

[47] Lyakhova E.G., Kolesnikova S.A., Kalinovsky A.I., Dmitrenok P.S. (2015). Further study on *Penares* sp. from Vietnamese waters: Minor lanostane and nor-lanostane triterpenes. Steroids.

[48] Shin J., Seo Y., Rho J.-R., Cho K.W. (1996). Isolation Polyhydroxysteroids from the Gorgonian *Acabaria undulate*. J. Nat. Prod..

[49] Thao N.P., Cuong N.X., Luyen B.T.T., Nam N.H. (2013). Steroidal constituents from the starfish *Astropecten polyacanthus* and their anticancer effects. Chem. Pharm. Bull..

[50] Sugo Y., Inouye Y., Nakayama N. (1995). Structures of nine oxygenated 4-methylene sterols from Hachijo marine sponge *Theonella swinhoei*. Steroids.

[51] Mansoor T.A., Lee Y.M., Hong J., Lee C.-O., Im K.S., Jung J.H. (2006). 5,6:8,9-Diepoxy and other cytotoxic sterols from the marine sponge *Homaxinella* sp.. J. Nat. Prod..

[52] Campbell D.C. (1974). Elistanol: A Novel Marinetterol, Dissertation.

[53] Costantino V., Fattorusso E., Mangoni A., Aknin M., Gaydou E.M. (1994). Novel 3-β-methoxysteroids from the senegalse sponge *Microscleroderma spirophora*. Steroids.

[54] de Almeida Leone P., Redburn J., Hooper J.N.A., Quinn R.J. (2000). Polyoxygenated *Dysidea* sterols that inhibit the binding of [I125] IL-8 to the human recombinant IL-8 receptor type A. J. Nat. Prod..

[55] Govindam S.V.S., Choi B.-K., Yoshioka Y., Kanamoto A., Fujiwara T., Okamoto T., Ojika M. (2012). Novel cytotoxic polyoxygenated steroids from an Okinawan sponge *Dysidea* sp.. Biosci. Biotechnol. Biochem..

[56] Alam M., Sanduja R., Weinheimer A.J. (1988). Isolation and structure of a cytotoxic epoxy sterol from the marine mollusc *Planaxis Sulcatus*. Steroids.

[57] Migliuolo A., Notaro G., Piccialli V., Sica D. (1991). Synthesis of the marine epoxy sterol 9a,11a-epoxy-5a-cholest-7-ene-3b,5,6b-triol. Steroids.

[58] Chini M.G., Jones C.R., Zampella A., D’Auria M.V., Renga B., Fiorucci S., Butts C.P., Bifulco G. (2012). Quantitative NMR-derived interproton distances combined with quantum mechanical calculations of 13C chemical shifts in the stereochemical determination of conicasterol F, a nuclear receptor ligand from *Theonella swinhoei*. J. Org. Chem..

[59] Ramesha P., Venkateswarlua Y. (1999). Novel steroid constituents of the soft coral *Sinularia dissecta*. Steroids.

[60] Miyamoto T., Sakamoto K., Arao K., Komori T., Higuchi R., Sasaki T. (1996). Dorisenones, cytotoxic spongian diterpenoids, from the Nudibranch *Chromodoris obsolete*. Tetrahedron.

[61] Shen L., Li W.S., Yu Y., Sun S.H., Wu J. (2021). A Water-soluble 5/14-carbobicyclic steroid with a trans-9,11-epoxy ring from the marine dinoflagellate *Amphidinium gibbosum*: Insights into late-stage diversification of steroids. Org. Lett..

[62] D’Auria M.V., Paloma L.G., Minale L., Riccio R., Debitus C., Lévi C. (1992). Unique 3β-O-methylsterols from the Pacific sponge *Jereicopsis graphidiophora*. J. Nat. Prod..

[63] Yang M.Y., Yang J.K., Yang J.K., Hu L.D. (2018). New oxygenated steroid from the marine-derived fungus *Aspergillus flavus*. Nat. Prod. Commun..

[64] An X., Feng B.-M., Chen G., Chen S.-F., Wang H.-F., Pei Y.-H. (2016). Isolation, and identification of two new compounds from marine-derived fungus *Acremonium fusidioides* RZ01. Chin. J. Nat. Med..

[65] Youssef D.T.A., Badr J.M., Shaala L.A., Mohamed G.A. (2015). Ehrenasterol and biemnic acid; new bioactive compounds from the Red Sea sponge *Biemna ehrenbergi*. Phytochem. Lett..

[66] Elsbaey M., Ibrahim M.A.A., Hegazy M.E.F. (2022). Versisterol, a new endophytic steroid with 3CL protease inhibitory activity from *Avicennia marina* (Forssk.) Vierh. RSC Adv..

[67] Yaoita Y., Yoshihara Y., Kakuda R., Machida K., Kikuchi M. (2002). New sterols from two edible mushrooms, *Pleurotus eryngii* and *Panellus serotinus*. Chem. Pharm. Bull..

[68] Bakhshi Jouybari H., Bekhradnia A., Mirzaee F., Hossein Hosseinzadeh M., Habibi E. (2022). Chemical composition of the lumpy bracket mushroom (*Trametes gibbosa)*. Res. J. Pharmacog..

[69] Zhang Q., Satyanandamurty T., Shen L., Wu J. (2017). Krishnolides A–D: New 2-ketokhayanolides from the Krishna mangrove, *Xylocarpus moluccensis*. Mar Drugs.

[70] Borges Coutinho Gallo M., Cavalcanti B.C., Barros F.W.A. (2010). Chemical Constituents of *Papulaspora immersa*, an endophyte from *Smallanthus sonchifolius* (Asteraceae), and their cytotoxic activity. Chem. Biodivers..

[71] Hu Z., Wu Y., Xie S., Sun W., Guo Y., Li X.N., Liu J. (2017). Phomopsterones A and B, two functionalized ergostane-type steroids from the endophytic fungus *Phomopsis* sp. TJ507A. Org. Lett..

[72] Anjaneyulu A.S.R., Krishna Murthy M.V.R., Gowri P.M. (2000). Novel epoxy steroids from the Indian ocean soft coral *Sarcophyton crassocaule*. J. Nat. Prod..

[73] Sheu J.-H., Chen S.P., Sung P.J., Chiang M.Y., Dai C. (2000). Hippuristerone A, a novel polyoxygenated steroid from the gorgonian *Isis hippuris*. Tetrahedron Lett..

[74] Sheu J.-H., Huang L.F., Chen S.P., Yang Y.L., Sung P.J. (2003). Hippuristerones E−I, new polyoxygenated steroids from the gorgonian coral *Isis hippuris*. J. Nat. Prod..

[75] Chen W.-H., Wang S.-K., Duh C.-Y. (2011). Polyhydroxylated steroids from the Bamboo coral *Isis hippuris*. Mar. Drugs.

[76] Zubair M.S., Al-Footy K.O., Ayyad S.E.N., Al-Lihaibi S.S., Alarif W.M. (2016). A review of steroids from Sarcophyton species. Nat. Prod. Res..

[77] Ye F., Zhou Y.B., Li J., Gu Y.C., Guo Y.W., Li X.W. (2020). New steroids from the South China Sea soft coral *Lobophytum* sp.. Chem. Biodivers..

[78] Funel C., Berrué F., Roussakis C., Rodriguez R.F., Amade P. (2004). New cytotoxic steroids from the Indian ocean sponge *Axinella* cf. bidderi. J. Nat. Prod..

[79] Sadri Said A. (2010). An epoxysterol and other constituents of Tanzania soft corals. Int. J. Biol. Chem. Sci..

[80] Tsukamoto S., Matsunaga S., Fusetani N., van Soest R.W.M. (1998). Acanthosterol sulfates A−J: Ten new antifungal steroidal sulfates from a marine sponge *Acanthodendrilla* sp.. J. Nat. Prod..

[81] Palagiano E., Zollo F., Minale L., Iorizzi M., Bryan P., McClintock J., Hopkins T. (1996). Isolation of 20 glycosides from the starfish *Henricia downeyae*, collected in the Gulf of Mexico. J. Nat. Prod..

[82] Tang H.-F., Yi Y.H., Li L., Sun P., Zhang S.Q., Zhao Y.P. (2005). Bioactive asterosaponins from the starfish *Culcita novaeguineae*. J. Nat. Prod..

[83] Kicha A.A., Ivanchina N.V., Huong T.T., Kalinovskiĭ A.I. (2010). Two new asterosaponins, archasterosides A and B, from the Vietnamese starfish *Archaster typicus* and their anticancer properties. Bioorg. Med. Chem. Lett..

[84] Kicha A.A., Ivanchina N.V., Kalinovskiĭ A.I., Dmitrenok P.S., Smirnov A.V. (2009). Two new steroid glycosides from the Far East starfish *Hippasteria kurilensis*. Bioorg. Khim..

[85] Schmidt J., Spengler B., Yokota T., Nakayama M., Takatsuto S., Voigt B., Adam G. (1995). Secasterone, the first naturally occurring 2,3-epoxybrassinosteroid from *Secale cereal*. Phytochemistry.

[86] Xiao Q., Wang C.F., Chen J., Lian C.L., Xu Y., Xiao L., Liu J.Q. (2018). Three new withanolides from the calyces of *Nicandra physaloides*. Steroids.

[87] Huang Y., Muehlbauer A., Henkel T., Liu J.-K. (2003). Two new taccalonolides from tropic plant *Tacca subflaellata*. Chin. Chem. Lett..

[88] Huang Y., Liu J.-K., Muhlbauer A., Henkel T. (2002). Three novel taccalonolides from the tropical plant *Tacca subflaellata*. Helv. Chim. Acta.

[89] Ray A.B., Gupta M. (1994). Withasteroids, a growing group of naturally occurring steroidal lactones. Prog. Chem. Org. Nat. Prod..

[90] Chen X., Winstead A., Yu H., Peng J. (2021). Taccalonolides: A novel class of microtubule-stabilizing anticancer agents. Cancers.

[91] Shen J., Chen Z., Gao Y. (1996). Taccalonolides from *Tacca plantaginea*. Phytochemistry.

[92] Chen Z.-L., Shen J., Gao Y., Wichtl M. (1997). Five Taccalonolides from *Tacca plantaginea*. Planta Med..

[93] Yang J.-Y., Zhao R.-H., Chen C.-X., Ni W., Teng F., Hao X.-J., Liu H.-Y. (2008). Taccalonolides W–Y, three new pentacyclic steroids from *Tacca plantaginea*. Helv. Chim. Acta.

[94] Muhlbauer A., Seip S., Nowak A., Tran V.S. (2003). Five novel taccalonolides from the roots of the Vietnamese plant *Tacca paxiana*. Helv. Chim. Acta.

[95] Liu H.-Y., Ni W., Xie B.-B., Zhou L.-Y., Hao X.-J., Wang X., Chen C.-X. (2006). Five new withanolides from *Tacca plantaginea*. Chem. Pharm. Bull..

[96] Jahan E., Perveen S., Fatima I., Malik A. (2010). Coagulansins A and B, new withanolides from *Withania coagulans Dunal*. Helv. Chim. Acta.

[97] Abdeljebbar L.H., Humam M., Christen P., Jeannerat D., Vitorge B., Amzazi S., Benjouad A., Hostettmann K., Bekkouche K. (2007). Withanolides from *Withania adpressa*. Helv. Chim. Acta.

[98] Maurya R., Jayendra A., Singh A.B., Srivastava A.K. (2008). Coagulanolide, a withanolide from *Withania coagulans* fruits and antihyperglycemic activity. Bioorg. Med. Chem. Lett..

[99] Nagafuji S., Okabe H., Akahane H., Abe F. (2004). Trypanocidal constituents in plants 4. Withanolides from the aerial parts of Physalis angulata. Biol. Pharm. Bull..

[100] Siddiqui B.S., Afreen S., Begum S. (1999). Two new withanolides from the aerial parts of *Datura innoxia*. Aust. J. Chem..

[101] Siddiqui B.S., Hashmi I.A., Begum S. (2002). Two new withanolides from the aerial parts of *Datura innoxia*. Heterocycles.

[102] Siddiqui B.S., Arfeen S., Afshan F., Begum S. (2005). Withanolides from *Datura innoxia*. Heterocycles.

[103] Kikuchi T., Horii Y., Maekawa Y., Masumoto Y., In Y. (2017). Pleurocins A and B: Unusual 11(9 → 7)-abeo-ergostanes and eringiacetal B: A 13,14-seco-13,14-epoxyergostane from fruiting bodies of *Pleurotus eryngii* and their inhibitory effects on nitric oxide production. J. Org. Chem..

[104] Ngoc N.T., Huong P.T., Thanh N.V., Cuong N.X., Nam N.H., Thung D.C., Kiem P.V., Minh C.V. (2016). Steroid constituents from the soft coral *Sinularia nanolobata*. Chem. Pharm. Bull..

[105] Liu Y.P., Cai X.H., Feng T., Li Y., Li X.N., Luo X.D. (2011). Triterpene, and sterol derivatives from the roots of *Breynia fruticose*. J. Nat. Prod..

[106] Ortega H.E., Torres-Mendoza D., Caballero E.Z., Cubilla-Rios L. (2021). Structurally uncommon secondary metabolites derived from endophytic fungi. J. Fungi.

[107] Tuan Anh H.H.L., Lien L.T., Cuong P.V., Arai M., Ha T.P. (2018). Sterols, and flavone from the leaves of *Vernonia amygdalina* growing in Thua Thien Hue. Vietnam J. Sci. Technol..

[108] Dung D.T., Hang D.T.T., Nhiem N.X. (2018). Rhabdaprovidines D–G, four new 6,6,5-tricyclic terpenoids from the Vietnamese sponge *Rhabdastrella providentiae*, *Nat*. Prod. Commun..

[109] Zeng N., Shen Y., Li L.Z., Jiao W.H., Gao P.Y. (2011). Anti-inflammatory triterpenes from the leaves of *Rosa laevigata*. J. Nat. Prod..

[110] Jamroz M.K., Jamroz M.H., Dobrowolski J.C., Glinski J.A. (2011). Novel and unusual triterpene from black cohosh. Determination of structure of 9,10-seco-9,19-cyclolanostane xyloside (cimipodocarpaside) by NMR, IR, and Raman spectroscopy and DFT calculations. Spectrochim. Acta.

[111] Chen J.X., Chen J.C., Sun Y., Yan Y.X., Kong L.M., Li Y., Qiu M.H. (2011). Cytotoxic triterpenoids from *Azadirachta indica*. Planta Med..

[112] Wong C.P., Shimada M., Nagakura Y., Nugroho A.E., Hirasawa Y. (2011). Ceramicines E—I, new limonoids from *Chisocheton ceramicus*. Chem. Pharm. Bull..

[113] Saeki D., Yamada T., In Y., Kajimoto T., Tanaka R., Iizuka Y. (2013). Officinatrione: An unusual (17S)-17, 18-seco-lupane skeleton, and four novel lupane-type triterpenoids from the roots of *Taraxacum officinale*. Tetrahedron.

[114] Zhang Y.-L., Feng W.S., Zheng X.K., Cao Y.G., Lv Y.Y. (2013). Three new ursane-type triterpenes from the leaves of *Rehmannia glutinosa*. Fitoterapia.

[115] Mai Z.P., Zhou K., Ge G.B., Wang C., Huo X.K. (2015). Protostane triterpenoids from the rhizome of *Alisma orientale* exhibit inhibitory effects on human carboxylesterase 2. J. Nat. Prod..

[116] Zhou Q.-L., Yang X.-W. (2015). Four new ginsenosides from red ginseng with inhibitory activity on melanogenesis in melanoma cells. Bioorg. Med. Chem. Lett..

[117] Choi E., Jang E., Lee J.H. (2019). Pharmacological activities of Alisma orientale against nonalcoholic fatty liver disease and metabolic syndrome: Literature Review. Evid. Based Complem. Altern. Med..

[118] Jiang Z.Y., Zhang X.M., Zhang F.X., Liu N., Zhao F., Zhou J., Chen J.J. (2006). A new triterpene and anti-hepatitis B virus active compounds from *Alisma orientalis*. Planta Med..

[119] Yoshikawa M., Hatakeyama S., Tanaka N., Fukuda Y., Yamahara J. (1993). Crude drugs from aquatic plants. I. On the constituents of *Alismatis rhizoma*. (1). Absolute stereostructures of alisols E 23-acetate, F, and G, three new protostane-type triterpenes from Chinese *Alismatis rhizoma*. Chem. Pharm. Bull..

[120] Zhao M., Gödecke T., Gunn J., Duan J.A., Che C.T. (2013). Protostane and fusidane triterpenes: A mini review. Molecules.

[121] Zhang J., Jin Q., Wu W. (2021). Force iteration molecular designing strategy for the systematic characterization and discovery of new protostane triterpenoids from *Alisma rhizoma* by UHPLC/LTQ-Orbitrap-MS. Anal. Bioanal. Chem..

[122] Sun C.P., Zhang J., Zhao W.Y., Yi J., Yan J.K., Wang Y.L. (2020). Protostane-type triterpenoids as natural soluble epoxide hydrolase inhibitors: Inhibition potentials and molecular dynamics. Bioorg Chem..

[123] De Marino S., Ummarino R., D’Auria M., Chini M.G. (2011). Theonellasterols and conicasterols from *Theonella swinhoei*. Novel marine natural ligands for human nuclear receptors. J. Med. Chem..

[124] Leon F., Valencia M., Rivera A., Nieto I., Quintana J., Estevez F., Bermejo J. (2003). Novel cytostatic lanostanoid triterpenes from *Ganoderma australe*. Helv. Chim. Acta.

[125] Yoshikawa K., Nishimura N., Bando S., Arihara S., Matsumura E., Katayama S. (2002). New lanostanoids, elfvingic acids A-H, from the fruit body of *Elfvingia applanata*. J. Nat. Prod..

[126] Pan C., Chen Y.G., Ma X.Y., Jiang J.H., He F., Zhang Y. (2011). Phytochemical constituents and pharmacological activities of plants from the genus *Adiantum*: A review. Trop. J. Pharm. Res..

[127] Hartmann R., Breitmaier E., Camargo Grandón R., Negrete Córdova R., Backhouse Erazo N., Delporte Vergara C., Cassels Niven B. (1992). One- and two-dimensional NMR in the structure determination of 3B-acetoxy-17B,21B-epoxyhopane from *Centaurea chilensis*. J. Praktisch. Chem..

[128] Du L., Zhu T., Fang Y., Gu Q., Zhu W. (2008). Unusual C25 steroid isomers with bicyclo[4.4.1]A/B rings from a volcano ash-derived fungus Penicillium citrinum. J. Nat. Prod..

[129] Cueto M., Jensen P.R., Fenical W. (2002). Aspergilloxide, a novel sesterterpene epoxide from a marine-derived fungus of the genus *Aspergillus*. Org. Lett..

[130] Hu Z.X., Shi Y.M., Wang W.G., Li X.N. (2015). Kadcoccinones A–F, new biogenetically related lanostane-type triterpenoids with diverse skeletons from *Kadsura coccinea*. Org. Lett..

[131] Vil V.A., Gloriozova T.A., Poroikov V.V., Terent’ev A.O., Savidov N., Dembitsky V.M. (2018). Peroxy steroids derived from plant and fungi and their biological activities. Appl. Microbiol. Biotechnol..

[132] Savidov N., Gloriozova T.A., Poroikov V.V., Dembitsky V.M. (2018). Highly oxygenated isoprenoid lipids derived from fungi and fungal endophytes: Origin and biological activities. Steroids.

[133] Vil V.A., Terent’ev A.O., Savidov N., Gloriozova T.A., Poroikov V.V., Pounina T.A., Dembitsky V.M. (2019). Hydroperoxy steroids and triterpenoids derived from plant and fungi: Origin, structures, and biological activities. J. Steroid Biochem. Mol. Biol..

[134] Zhabinskii V.N., Drasar P., Khripach V.A. (2022). Structure and biological activity of ergostane-type steroids from fungi. Molecules.

[135] Zhabinskii V.N., Khripach N.B., Khripach V.A. (2015). Steroid plant hormones: Effects outside plant kingdom. Steroids.

[136] Panibrat O.V., Zhabinskii V.N., Khripach V.A., Hayat S., Yusuf M., Bhardwaj R., Bajguz A. (2019). Anticancer potential of brassinosteroids. Brassinosteroids: Plant Growth and Development.

[137] Khripach V., Zhabinskii V., de Groot A. (2000). Twenty years of brassinosteroids: Steroidal plant hormones warrant better crops for the XXI century. Annal. Bot..

[138] Aly A.H., Debbab A., Proksch P. (2011). Fungal endophytes: Unique plant inhabitants with great promises. Appl. Microbiol. Biotechnol..

[139] Rodriguez R.J., White J.F., Arnold A.E., Redman R.S. (2009). Fungal endophytes: Diversity and functional roles. New Phytol..

[140] Suryanarayanan T.S., Thirunavukkarasu N., Govindarajulu M.B., Sasse F., Jansen R., Murali T.S. (2009). Fungal endophytes and bioprospecting. Fungal Biol. Rev..

[141] Zhao Z.-Z., Han K.-Y., Li Z.-H., Feng T., Chen H.-P., Liu J.-K. (2019). Cytotoxic ergosteroids from the fungus *Stereum hirsutum*. Phytochem. Lett..

[142] Li L.-N., Wang L., Guo X.-L. (2019). Chemical constituents from the culture of the fungus *Hericium alpestre*. J. Asian Nat. Prod. Res..

[143] Zheng J., Wang Y., Wang J., Liu P., Li J., Zhu W. (2013). Antimicrobial ergosteroids and pyrrole derivatives from halotolerant *Aspergillus flocculosus* PT05-1 cultured in a hypersaline medium. Extremophiles.

[144] Zhao J.-L., Zhang M., Liu J.-M., Tan Z., Chen R.-D., Xie K.-B., Dai J.-G. (2017). Bioactive steroids and sorbicillinoids isolated from the endophytic fungus *Trichoderma* sp. Xy24. J. Asian Nat. Prod. Res..

[145] Palasarn S., Intereya K., Boonpratuang T., Thongpanchang C., Isaka M. (2022). Ergostane triterpenoids from the cultures of basidiomycete *Favolaschia calocera* BCC 36684 and stereochemical elucidation of favolon. Phytochem. Lett..

[146] Gu B.B., Wu W., Jiao F.R., Jiao W.H., Li L., Sun F., Wang S.P., Yang F., Lin H.W. (2019). Asperflotone, an 8(14->15)-abeo-ergostane from the sponge-derived fungus *Aspergillus flocculosus* 16D-1. J. Org. Chem..

[147] Duecker F.L., Franziska Reuß F., Heretsch P. (2019). Rearranged ergostane-type natural products: Chemistry, biology, and medicinal aspects. Org. Biomol. Chem..

[148] Xue J., Wu P., Xu L., Wei X. (2014). Penicillitone, a potent in vitro anti-inflammatory and cytotoxic rearranged sterol with an unusual tetracycle core produced by *Penicillium purpurogenum*. Org. Lett..

[149] Wu J., Tokuyama S., Nagai K., Yasuda N., Noguchi K., Matsumoto T., Hirai H., Kawagishi H. (2012). Strophasterols A to D with an unprecedented steroid skeleton: From the mushroom *Stropharia rugosoannulata*. Angew. Chem. Int. Ed. Engl..

[150] Kikuchi T., Isobe M., Uno S., In Y., Zhang J., Yamada T. (2019). Strophasterols E and F: Rearranged ergostane-type sterols from *Pleurotus eryngii*. Bioorg. Chem..

[151] Gao H., Hong K., Chen G.D., Wang C.X., Tang J.S., Yu Y., Jiang M.M., Li M.M., Wang N.L., Yao X.S. (2010). New oxidized sterols from *Aspergillus awamori* and the endo-boat conformation adopted by the cyclohexene oxide system. Magn. Reson. Chem..

[152] Wang J.-P., Shu Y., Liu S.-X., Hu J.-T., Sun C.-T., Zhou H., Gan D., Cai X.-Y., Pu W. (2019). Expanstines A–D: Four unusual isoprenoid epoxycyclohexenones generated by *Penicillium expansum* YJ-15 fermentation and photopromotion. Org. Chem. Front..

[153] Schmidt L.E., Deyrup S.T., Baltrusaitis J., Swenson D.C., Wicklow D.T. (2010). Hymenopsins A and B and a macrophorin analogue from a fungicolous *Hymenopsis* sp.. J. Nat. Prod..

[154] Tian M., Zhao P., Li G., Zhang K. (2020). In depth natural product discovery from the Basidiomycetes Stereum species. Microorganisms.

[155] Berovic M. (2019). Cultivation of medicinal mushroom biomass by solid-state bioprocessing in bioreactors. Adv. Biochem. Eng. Biotechnol..

[156] Carroll A.R., Brent R., Davis R.A., Keyzers R.A., Prinsep M.R. (2020). Marine natural products. Nat. Prod. Rep..

[157] Vaquero M.E., Barriuso J., Martínez M.J. (2016). Properties, structure, and applications of microbial sterol esterases. Appl. Microbiol. Biotechnol..

[158] Moussa A.Y., Xu B. (2023). A narrative review on inhibitory effects of edible mushrooms against malaria and tuberculosis-the world’s deadliest diseases. Food Sci. Human Wellness.

[159] Yurchenko A.N., Girich E.V., Yurchenko E.A. (2021). Metabolites of marine sediment-derived fungi: Actual trends of biological activity studies. Mar. Drugs.

[160] Wang Z., Hui C. (2021). Contemporary advancements in the semi-synthesis of bioactive terpenoids and steroids. Org. Biomol. Chem..

[161] Lindsay C.A., Kinghorn A.D., Rakotondraibe H.L. (2023). Bioactive and unusual steroids from *Penicillium fungi*. Phytochemistry.

[162] Huang L., He C., Si C., Shi H., Duan J. (2023). Nutritional, Bioactive, and Flavor Components of Giant Stropharia (Stropharia rugoso-annulata): A Review. J. Fungi.

[163] Aung H.T., Porta A., Clericuzio M., Takaya Y., Vidari G. (2017). Two new ergosterol derivatives from the basidiomycete *Cortinarius glaucopus*. Chem. Biodivers..

[164] Li W., Zhou W., Song S.B., Shim S.H., Kim Y.H. (2014). Sterol fatty acid esters from the mushroom *Hericium erinaceum* and their ppar transactivational effects. J. Nat. Prod..

[165] Elissawy A.M., El-Shazly M., Ebada S.S., Singa A.N., Proksch P. (2015). Bioactive terpenes from marine-derived fungi. Mar. Drugs.

[166] Lagrouh F., Dakka N., Bakri Y. (2017). The antifungal activity of Moroccan plants and the mechanism of action of secondary metabolites from plants. J. Mycol. Méd..

[167] Noinart J., Buttachon S., Dethoup T., Gales L., Pereira J.A., Urbatzka R. (2017). A new ergosterol analog, a new bis-anthraquinone and anti-obesity activity of anthraquinones from the marine sponge-associated fungus *Talaromyces stipitatus* KUFA 0207. Mar. Drugs.

[168] Liu X.H., Tang X.Z., Miao F.P., Ji N.Y. (2011). A new pyrrolidine derivative and steroids from an algicolous Gibberella *zeae strain*. Nat. Prod. Commun..

[169] Simon A., Tóth G., Liktor-Busa E., Kele Z., Takács M., Gergely A., Báthori M. (2007). Three new steroids from the roots of *Serratula wolffii*. Steroids.

[170] Wang P., Song T., Shi R., He M., Wang R., Lv J., Jiang M. (2020). Triterpenoids from Alisma species: Phytochemistry, structure modification, and bioactivities. Front. Chem..

[171] Bailly C. (2022). Pharmacological properties and molecular targets of alisol triterpenoids from *Alismatis Rhizoma*. Biomedicines.

[172] Liang C.Q., Shi Y.M., Luo R.H., Li X.Y., Gao Z.H., Li X.N. (2012). Kadcoccitones A and B, two new 6/6/5/5-fused tetracyclic triterpenoids from *Kadsura coccinea*. Org Lett..

[173] Chen J.C., Liu W.Q., Lu L., Qiu M.H., Zheng Y.T., Yang L.M., Zhang X.M., Zhou L., Li Z.R. (2009). Kuguacins F-S, cucurbitane triterpenoids from *Momordica charantia*. Phytochem..

[174] Wu H.F., Morris-Natschke S.L., Xu X.D. (2020). Recent advances in natural anti-HIV triterpenoids and analogs. Med. Res. Rev..

[175] Gutiérrez-Nicolás F., Gordillo-Román B., Oberti J.C., Estévez-Braun A., Ravelo Á.G., Joseph-Nathan P. (2012). Synthesis and anti-HIV activity of lupane and olean-18-ene derivatives. Absolute configuration of 19,20-epoxylupanes by VCD. J. Nat. Prod..

[176] Huang K.-F., Sy M.-L., Lai J.-S. (1990). A new pentacyclic triterpene from *Ecdysanthera rosea*. J. Chin. Chem. Soc..

[177] Liu F., Wang Y.N., Li Y. (2019). Triterpenoids from the twigs and leaves of Rhododendron latoucheae by HPLC-MSSPE-NMR. Tetrahedron.

[178] Liang C.Q., Luo R.H., Yan J.M. (2014). Structure and bioactivity of triterpenoids from the stems of *Schisandra sphenanthera*. Arch. Pharm. Res..

[179] Song Q.Y., Jiang K., Zhao Q.Q. (2013). Eleven new highly oxygenated triterpenoids from the leaves and stems of *Schisandra chinensis*. Org. Biomol. Chem..

[180] Polturak G., Dippe M., Stephenson M.J., Chandra Misra R., Owen C., Ramirez-Gonzalez R.H., Haidoulis J.F., Schoonbeek H.-J., Chartrain L., Borrill P. (2022). Pathogen-induced biosynthetic pathways encode defense-related molecules in bread wheat. Proc. Natl. Acad. Sci. USA.

[181] Graziani E.I., Allen T.M., Andersen R.J. (1995). Lovenone, a cytotoxic degraded triterpenoid isolated from skin extracts of the North Sea dorid nudibranch *Adalaria loveni*. Tetrahedron Lett..

[182] Su H.G., Liang H.F., Hu G.L., Zhou L., Peng X.P., Bi H.C., Qiu M.H. (2022). Applanoids A—E as the first examples of C-15/C-20 michael adducts in Ganoderma triterpenoids and their PXR agonistic activity. Chin. J. Chem..

[183] Amagata T., Doi M., Ohta T., Minoura K., Numata A. (1998). Absolute stereostructures of novel cytotoxic metabolites, gymnastatins A-E, from a Gymnascella species separated from a *Halichondria* sponge. J. Chem. Soc. Perkin Trans. 1.

[184] Amagata T., Minoura K., Numata A. (1998). Gymnasterones, novel cytotoxic metabolite produced by a fungal strain from sponge. Tetrahedron Lett..

[185] Amagata T., Minoura K., Numata A. (2006). Gymnastatins F-H, cytostatic metabolites from the sponge-derived fungus *Gymnascella dankaliensis*. J. Nat. Prod..

[186] Nicoletti R., Bellavita R., Falanga A. (2023). The outstanding chemodiversity of marine-derived Talaromyces. Biomolecules.

[187] Harneti D., Supriadin A., Ulfah M., Safari A., Supratman U., Awang K., Hayashi H. (2014). Cytotoxic constituents from the bark of *Aglaia eximia* (Meliaceae). Phytochem. Lett..

